# Synthesis of α-amino amidines through molecular iodine-catalyzed three-component coupling of isocyanides, aldehydes and amines

**DOI:** 10.3762/bjoc.10.214

**Published:** 2014-09-02

**Authors:** Praveen Reddy Adiyala, D Chandrasekhar, Jeevak Sopanrao Kapure, Chada Narsimha Reddy, Ram Awatar Maurya

**Affiliations:** 1 Division of Medicinal Chemistry and Pharmacology, CSIR-Indian Institute of Chemical Technology, Hyderabad-500007, India

**Keywords:** α-amino amidines, iodine, isocyanide, multicomponent reaction, Ugi reaction

## Abstract

A facile and efficient synthetic protocol for the synthesis of α-amino amidines has been developed using a molecular iodine-catalyzed three-component coupling reaction of isocyanides, amines, and aldehydes. The presented strategy offers the advantages of mild reaction conditions, low environmental impact, clean and simple methodology, high atom economy, wide substrate scope and high yields.

## Introduction

Amidines are a class of organic compounds exhibiting a variety of biological activity that makes them potential candidates for drug development and discovery [1–5]. Simple amidines are generally synthesized from their corresponding nitriles either by the Pinner reaction [6] or by the thioimidate route [7]. Recently, much attention was given to the development of new routes for the synthesis of substituted amidines [8–11]. Even if these methods provide amidines in acceptable yields, they suffer from limitations such as limited structural diversity of the final products. Since multicomponent reactions (MCRs) are expected to provide a rich structural diversity, much attention was paid on the development of multicomponent-coupling strategies for the synthesis of amidines.

**Figure 1:**
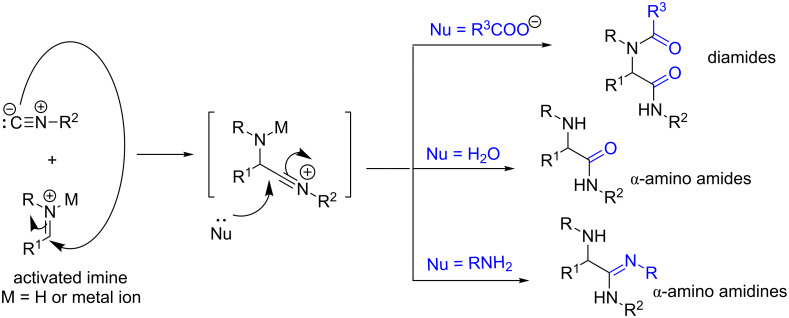
Synthesis of diamides, α-amino amides [13–14] and α-amino amidines [15–19] through Ugi and related MCRs.

The Ugi reaction is probably one of the best multicomponent reactions to provide huge structural diversification of the products [12]. Thus, several modifications of the Ugi reaction were explored recently. As depicted in Figure 1, diamides, α-amino amides, and α-amino amidines can be obtained depending on the nucleophile used. However, the reaction does not lead to acceptable product yields of products without using proper catalysts except when the nucleophile is carboxylate. For instance, among various catalysts screened, only phosphinic acid and boric acid were found suitable for conversion of substrates into products when water was used as a nucleophile for amide preparations [13–14]. In the direction of amidine synthesis using isocyanide MCRs, a few catalysts such as *p*-toluenesulfinic acid [15], metal triflates [16], bromodimethylsulfonium bromide [17], ZnO nanoparticles [18] and BF_3_·OEt_2_ [19] were reported with varying degrees of success. All these reported methods for the preparation of α-amino amidines have their own limitations such as long reaction times, high catalyst loading and use of expensive and hazardous metal catalysts. Therefore, the development of a mild, inexpensive and efficient catalytic protocol for the amidine synthesis is highly needed.

Iodine is expected to act as a Lewis acid or Brønsted acid in methanol [20]. Apart from oxidation, catalytic iodine provides mild and efficient ways for the formation of C–C and C–N bonds [20]. As a part of our ongoing interest towards the synthesis of new molecular libraries [21–24], we were interested in developing a one-pot MCR strategy for the synthesis of amidines.

## Results and Discussion

To check the feasibility of the iodine-catalyzed amidine synthesis through the modified Ugi reaction, we carried out a model reaction of *tert*-butyl isocyanide (1 mmol), benzaldehyde (1 mmol), and aniline (2 mmol) using 5 mol % of molecular iodine in methanol (Table 1). The reaction worked well at ambient temperature and led to good yields of **4a**. Among various solvents screened, methanol was found to be the best choice as solvent for the reaction. Furthermore, we observed that the catalyst loading could be reduced to 1 mol % without affecting the product yield. Further decreasing the amount of catalyst (0.5 mol %) still lead to a good yield of **4a**, albeit with a longer reaction time (Table 1, entry 5). It was interesting to notice a significant decrease in the product yield when the catalyst was overloaded (Table 1, entries 6 and 7). When the reaction was carried out without catalyst (iodine), no product was observed (Table 1, entry 11). This observation confirmed that catalytic iodine is necessary for the success of the reaction.

**Table 1:** Synthesis of α-amino amidine **4a** through a three-component coupling of benzaldehyde, aniline, and *tert*-butyl isocyanide.^a^

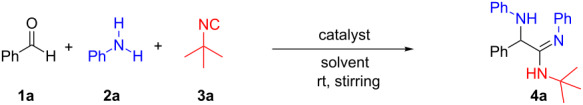				

Entry	Catalyst (I_2_)	Solvent	Time (h)	Yield of **4a** (%)^b^

1	5 mol %	methanol	2	90
2	2 mol %	methanol	2	91
3	1 mol %	methanol	2	90
4	0.5 mol %	methanol	2	78
5	0.5 mol %	methanol	6	88
6	10 mol %	methanol	2	85
7	20 mol %	methanol	2	70
8	1 mol %	ethanol	2	85
9	1 mol %	acetonitrile	2	78
10	1 mol %	THF	2	72
11	none	methanol	24	0

^a^Composition of reaction mixture: Benzaldehyde (1 mmol), aniline (2 mmol), *tert*-butyl isocyanide (1 mmol), solvent (5 mL), I_2_, rt, stirring. ^b^Isolated yields which are not optimized.

Next we studied the substrate compatibility of the reaction to generalize the scope of the α-amino amidine synthesis (Table 2). Aliphatic, aromatic and heteroaromatic aldehydes were used with similar success leading to high yields of amidines. It is worth to note here that aldehydes containing an alkyne moiety yielded the corresponding amidines with similar success (Table 2, entries 17 and 18). With aromatic amines, the reaction was good; with aliphatic amines (for instance benzylamine) the reaction was sluggish and the desired amidine was not obtained. The reaction worked well with a variety of isocyanides such as *tert*-butyl isocyanide, cyclohexyl isocyanide, and more importantly with functional groups bearing isocyanides such as ethyl isocyanoacetate and *p*-toluenesulfonylmethyl isocyanide (*p*-TosMIC) (Table 2, entries 19 and 20). Thus, the diversification of the α-amino amidine was achieved by varying the aldehyde, aromatic amine and isocyanide components of the reaction. The iodine-catalyzed protocol gave better yields (75–93%) of amidines than a recently reported *p*-toluenesulfinic acid [15] catalyzed protocol (52–71%). In contrary to the *p*-toluenesulfinic acid-catalyzed protocol, the formation of byproducts (α-amino amides) was suppressed in our iodine-catalyzed protocol which gave rise to better yields and cleaner products. When compared with other related reports [16–19], our iodine-catalyzed protocol gave similar yields of α-amino amidines. However, it should be emphasized that our protocol with low catalyst loading (1 mol %) makes it a cleaner and lower environmental impact methodology to access α-amino amidines.

**Table 2:** Scope of the α-amino amidine synthesis through three-component coupling of aldehyde, amine, and isocyanide.^a^

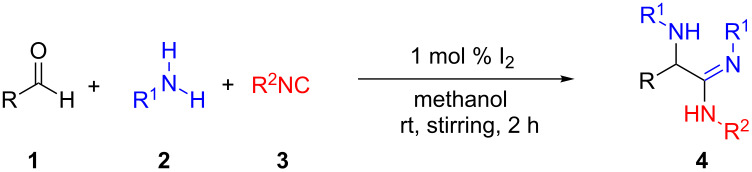					

Entry	R	R^1^	R^2^	Product	Yield of product (%)^b^

1	C_6_H_5_	C_6_H_5_	(CH_3_)_3_C	**4a**	90
2	C_6_H_5_	C_6_H_5_	*c*-C_6_H_11_	**4b**	93
3	2-Br-C_6_H_4_	C_6_H_5_	(CH_3_)_3_C	**4c**	85
4	*c*-C_6_H_11_	C_6_H_5_	*c*-C_6_H_11_	**4d**	86
5	*n*-C_8_H_17_	C_6_H_5_	*c*-C_6_H_11_	**4e**	88
6	4-Cl-C_6_H_4_	C_6_H_5_	*c*-C_6_H_11_	**4f**	88
7	2-Furyl	C_6_H_5_	(CH_3_)_3_C	**4g**	90
8	*c*-C_6_H_11_	C_6_H_5_	(CH_3_)_3_C	**4h**	90
9	*n*-C_8_H_17_	C_6_H_5_	(CH_3_)_3_C	**4i**	87
10	4-F-C_6_H_4_	C_6_H_5_	(CH_3_)_3_C	**4j**	88
11	4-Cl-C_6_H_4_	C_6_H_5_	(CH_3_)_3_C	**4k**	85
12	4-Cl-C_6_H_4_	4-CH_3_O-C_6_H_4_	(CH_3_)_3_C	**4l**	90
13	C_6_H_5_	4-CH_3_-C_6_H_4_	(CH_3_)_3_C	**4m**	86
14	4-F-C_6_H_4_	4-CH_3_-C_6_H_4_	(CH_3_)_3_C	**4n**	92
15	*n*-C_3_H_7_	4-CH_3_-C_6_H_4_	(CH_3_)_3_C	**4o**	91
16	*c*-C_6_H_11_	4-CH_3_-C_6_H_4_	(CH_3_)_3_C	**4p**	83
17	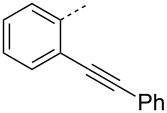	C_6_H_5_	(CH_3_)_3_C	**4q**	85
18	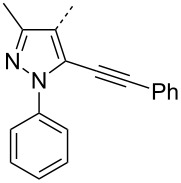	C_6_H_5_	(CH_3_)_3_C	**4r**	77
19	3,4,5-(MeO)_3_-C_6_H_2_	C_6_H_5_	EtOOCCH_2_	**4s**	75
20	3,4,5-(MeO)_3_-C_6_H_2_	C_6_H_5_	*p*-TolSO_2_CH_2_	**4t**	79

^a^Aldehyde (1 mmol), amine (2 mmol), isocyanide (1 mmol), iodine 1 mol %, methanol (5 mL), rt, stirring, 2 h. ^b^Isolated yield which is not optimized.

**Figure 2:**
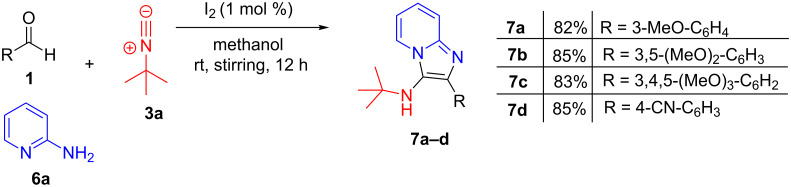
Synthesis of imidazolopyridines **7a–d** through a three-component coupling reaction of substituted benzaldehydes, 2-aminopyridine, and *tert*-butyl isocyanide using 1 mol % iodine as catalyst.

Then, we tried the three-component reaction using heteroaromatic amines such as 2-aminopyridine, 3-aminopyridine and 4-aminopyridine. The desired products (amidines) were not obtained with 3-aminopyridine and 4-aminopyridine. However, we found that iodine can efficiently catalyze the three-component coupling reaction of 2-aminopyridine, aldehyde and isocyanide (Groebke–Blackburn–Bienaymé reaction) (Figure 2) [25–27]. Recently, catalytic iodine (10 mol %) was found to give good yields of imidazolopyridine in a three-component reaction of 2-amino-5-chloropyridine, isocyanide, and aldehydes under reflux conditions [28]. However, we found that the similar reaction using 2-amonopyridine could be performed at ambient temperature using 1 mol % of iodine as catalyst to achieve a satisfactory yield of product (82–85%).

**Figure 3:**
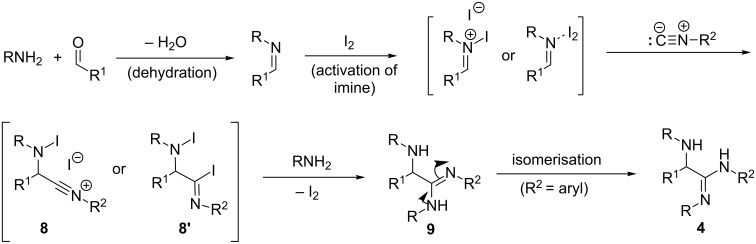
A plausible reaction mechanism for the iodine-catalyzed α-amino amidine synthesis.

A probable mechanistic pathway for the formation of α-amino amidines is outlined in Figure 3 which is analogous to the established mechanism reported in the literature [28–29]. Iodine can serve as a catalyst for the activation of imine. The attack of nucleophilic isocyanide on the activated imine leads to the formation of intermediate **8** or **8’**. Subsequently, another molecule of amine attacks the intermediate **8** or **8’** to give α-amino amidine **9** which undergoes further [1,3]-hydrogen shift to provide the α-amino amidines **4** [17].

## Conclusion

In conclusion, we have developed a simple and clean methodology for the synthesis of substituted α-amino amidines using a three-component coupling of isocyanide, aldehyde, and aromatic amines with molecular iodine as a catalyst. The current strategy provides elegant access to α-amino amidine and imidazolopyridines in high yield with significantly low catalyst loading.

## Experimental

A 25 mL round bottom flask was filled with aldehyde (1 mmol), amine (2 mmol)/2-aminopyridine (1 mmol), isocyanide (1 mmol) and MeOH (5 mL). Then, I_2_ (1 mol %) was added and the reaction mixture was stirred until the reaction was completed (TLC). The reaction mixture was evaporated to dryness using a rotary evaporator and the residue was purified by silica-gel column chromatography using a mixture of ethyl acetate/hexane as eluent in increasing polarity.

## Supporting Information

General information, general experimental procedure, characterization data of the synthesized compounds, and copies of ^1^H and ^13^C NMR spectra are given in Supporting Information File 1.

File 1 Experimental data.
