# Supplementary material for: Synthesis of α-amino amidines through molecular iodine-catalyzed three-component coupling of isocyanides, aldehydes and amines
Source: Beilstein J Org Chem. 2014 Sep 2;10:2065–70. doi: 10.3762/bjoc.10.214 (PMC4168875; doi:10.3762/bjoc.10.214)
Supplement: File 1 — Experimental data. [file Beilstein_J_Org_Chem-10-2065-s001.pdf]

Supporting Information

for

Synthesis of α-amino amidines through molecular iodine-catalyzed three-component coupling of isocyanides, aldehydes and amines

Praveen Reddy Adiyala, D. Chandrasekhar, Jeevak Sopanrao Kapure, Chada Narsimha Reddy and Ram Awatar Maurya*

Address: Division of Medicinal Chemistry and Pharmacology, CSIR-Indian Institute of Chemical Technology, Hyderabad-500007, India

Email: Ram Awatar Maurya - ramaurya@iict.res.in
* Corresponding author

Experimental data

Contents:

General information	Page S1
Typical experimental procedure for the amidine synthesis	Page S2
Characterization data for the synthesized compounds **4a-4t** & **7a-d**	Page S2–S7
References	Page S7
Copies of 1H and 13C spectra of the synthesized compounds **4a-4t** & **7a-d**	Page S8–S55

General information: All the reagents and chemicals were purchased from commercial sources and used without any further purification. Common laboratory solvents (LR grade) were purchased from domestic suppliers. Analytical thin layer chromatography was performed using E. Merck silica gel 60 F aluminium plates and visualized under UV 254 nm. NMR spectra were measured with Brucker 500, and 600 MHz instruments. Chemical shifts are reported in δ units, parts per million (ppm) downfield from TMS. Coupling constants (*J*) are in Hertz (Hz) and are unadjusted; therefore, due to limits in resolution, in

some cases there are small differences (<1 Hz) in the measured *J* value of the same coupling constant determined from different signals. Splitting patterns are designed as follows: s, singlet; d, doublet; t, triplet; dd, doublet of doublets; dt, doublet of triplets; tt, triplet of triplets; m, multiplet; br, broad. IR spectra were recorded on a Perkin–Elmer FT-IR RXI spectrophotometer and values reported in cm^{-1}. ESI-MS spectra were obtained on a LCQ Advantage Ion trap mass spectrometer (Finnigan thermo fischer scientific). All the compounds were characterized by ^{1}H NMR, ^{13}C NMR, IR, and ESI-MS/HRMS spectroscopy.

Typical experimental procedure for the amidine synthesis: In a 25 ml round bottom flask, aldehyde (1 mmol), amine (2 mmol)/2-aminopyridine (1 mmol), isocyanide (1 mmol), and MeOH (5 ml) were taken. To this reaction mixture I_2 (1 mol%) was added and the reaction mixture was stirred until completion of the reaction (TLC). Next the reaction mixture was evaporated to dryness using a rotary evaporator and the residue was purified by silica-gel column chromatography using a mixture of ethyl acetate-hexane as eluent in increasing polarity.

Characterization data for the synthesized compounds 4a-4q & 7a

***N-tert*-Butyl-*N*',2-diphenyl-2-(phenylamino)acetimidamide (4a):**[1,2,3] White solid (322 mg, 90%); R_f (EtOAc/Hexane: 1/20) 0.61; ESI-MS (*m/z*): 358 $[M+H]^+$; IR (KBr) ν_{max}: 3289 (NH), 1634 (C=N); ^{1}H NMR ($CDCl_3$, 600 MHz) δ: 1.45 (s, 9H, $[CH_3]_3$), 3.68 (bs, 1H, NH), 4.92-4.93 (m, 1H, CH), 5.87 (s, 1H, NH), 6.48-6.51 (m, 2H, ArH), 6.65-6.67 (m, 2H, ArH), 6.78-6.81 (m, 2H, ArH), 7.00-7.03 (m, 2H, ArH), 7.12-7.25 (m, 7H, ArH); ^{13}C NMR ($CDCl_3$, 150.9 MHz) δ: 28.3, 50.8, 59.8, 113.7, 119.3, 121.0, 122.2, 127.9, 128.1, 128.3, 128.7, 129.2, 139.8, 147.0, 150.5, 153.8; HRMS (ESI): calcd for $C_{24}H_{28}N_3$ $[M+H]^+$ 358.22832; found 358.22800.

***N*-Cyclohexyl-*N*',2-diphenyl-2-(phenylamino)acetimidamide (4b):**[1,2] White solid (357 mg, 93%); R_f (EtOAc/Hexane: 1/20) 0.62; ESI-MS (*m/z*): 384 $[M+H]^+$; IR (KBr) ν_{max}: 3238 (NH), 1621 (C=N); ^{1}H NMR ($CDCl_3$, 600 MHz) δ: 1.27-1.55 (m, 5H, all from CH_2), 1.56-1.81 (m, 3H, all from CH_2), 2.10-2.23 (m, 2H, all from CH_2), 3.83 (s, 1H, NH), 4.05-4.06 (m, 1H, CH), 5.07 (s, 1H, NH), 6.00 (d, J = 7.2 Hz, 1H, CH), 6.60 (d, J = 7.8 Hz, 2H, ArH), 6.76 (d, J = 7.8 Hz, 2H, ArH), 6.90-6.94 (m, 2H, ArH), 7.12-7.14 (m, 2H, ArH), 7.20-7.26 (m, 2H, ArH), 7.30-7.38 (m, 5H, ArH); ^{13}C NMR ($CDCl_3$, 150.9 MHz) δ: 24.7, 24.9, 25.8, 32.5, 33.0, 48.4, 59.7, 113.7, 119.3, 121.4, 122.5, 127.9, 128.2, 128.3, 128.7, 129.2, 139.5, 147.0, 150.5, 155.0; HRMS (ESI): calcd for $C_{26}H_{30}N_3$ $[M+H]^+$ 384.24397; found 384.24251.

2-(2-Bromophenyl)-*N-tert*-butyl-*N*'-phenyl-2-(phenylamino)acetimidamide (4c): White solid (371 mg, 85%); R_f (EtOAc/Hexane: 1/20) 0.56; ESI-MS (*m/z*): 436 $[M+H]^+$; IR (KBr) ν_{max}: 3301 (NH), 1636 (C=N); ^{1}H NMR ($CDCl_3$, 600 MHz) δ: 1.49 (s, 9H, $[CH_3]_3$), 3.57 (s, 1H, NH), 5.23 (d, J = 1.8Hz, 1H, CH), 5.95 (s, 1H, NH), 6.41 (d, J = 7.2 Hz, 2H, ArH), 6.67-6.69 (m, 2H, ArH), 6.77-6.84 (m, 2H, ArH), 6.98-7.00 (m, 2H, ArH), 7.11 (t, J = 7.8 Hz, 1H, ArH), 7.20-7.23 (m, 2H, ArH), 7.30-7.31 (m, 1H, ArH), 7.40 (d, J = 8.4 Hz, 1H, 1H, ArH), 7.65-7.66 (m, 1H, ArH); ^{13}C NMR ($CDCl_3$, 150.9 MHz) δ: 28.3, 51.0, 59.4, 113.7, 119.5, 121.2, 121.7, 125.4, 127.6, 128.5, 129.1, 129.2, 129.7, 133.0, 139.0, 147.3, 150.4, 153.7; HRMS (ESI): calcd for $C_{24}H_{27}BrN_3$ $[M+H]^+$ 436.13884; found 436.13801.

***N*,2-Dicyclohexyl-*N*'-phenyl-2-(phenylamino)acetimidamide (4d):**[1] White solid (335 mg, 86%); R_f (EtOAc/Hexane: 1/20) 0.67; ESI-MS (*m/z*): 390 $[M+H]^+$; IR (KBr) ν_{max}: 3312 (NH), 1631 (C=N); ^1H NMR ($CDCl_3$, 600 MHz) δ: 0.90-2.08 (m, 21H, 10CH_2 &CH), 3.63 (s, 1H, NH), 3.90 (s, 1H, CH), 4.25 (d, J = 5.4 Hz, 1H, CH), 5.55 (s, 1H, NH), 6.74 (d, J = 7.8 Hz, 2H, ArH), 6.82 (t, J = 7.2Hz, 1H, ArH), 6.90-6.96 (m, 3H, ArH), 7.22-7.29 (m, 4H, ArH); ^{13}C NMR ($CDCl_3$, 150.9 MHz) δ: 24.7, 24.9, 25.8, 26.2, 26.5, 26.6, 27.0, 30.7, 32.6, 33.1, 40.6, 48.3, 59.3, 113.9, 118.8, 121.2, 122.4, 128.7, 129.2, 147.8, 150.9, 156.0; HRMS (ESI): calcd for $C_{26}H_{36}N_3$ $[M+H]^+$ 390.29092; found 390.29003.

***N*-Cyclohexyl-*N*'-phenyl-2-(phenylamino)decanimidamide (4e):**[1] White solid (370 mg, 88%); R_f (EtOAc/Hexane: 1/20) 0.68; ESI-MS (*m/z*): 420 $[M+H]^+$; IR (KBr) ν_{max}: 3221 (NH), 1635 (C=N); ^1H NMR ($CDCl_3$, 600 MHz) δ: 0.93-0.95 (m, 3H, CH_3), 1.02-1.37 (m, 10H, 5CH_2), 1.42-1.48 (m, 7H, all from CH_2), 1.61-2.09 (m, 7H, all from CH_2), 3.60 (s, 1H, NH), 3.88 (s, 1H, CH), 4.05 (d, J = 8.4 Hz, 1H, CH), 5.58 (s, 1H, NH), 6.73 (d, J = 7.8 Hz, 2H, ArH), 6.83 (t, J = 7.8Hz, 1H, ArH), 6.93-6.98 (m, 3H, ArH), 7.25-7.30 (m, 4H, ArH); ^{13}C NMR ($CDCl_3$, 150.9 MHz) δ: 14.0, 22.6, 24.7, 24.9, 25.6, 25.8, 28.8, 29.0, 29.1, 29.6, 31.7, 32.5, 32.9, 34.3, 48.2, 54.2, 113.7, 118.9, 121.3, 122.5, 128.8, 129.2, 147.4, 150.9, 157.5; HRMS (ESI): calcd for $C_{28}H_{42}N_3$ $[M+H]^+$ 420.33787; found 420.33622.

2-(4-Chlorophenyl)-*N*-cyclohexyl-*N*'-phenyl-2-(phenylamino)acetimidamide (4f):[1] White solid (368 mg, 88%); R_f (EtOAc/Hexane: 1/20) 0.55; ESI-MS (*m/z*): 418 $[M+H]^+$; IR (KBr) ν_{max}: 3335 (NH), 1634 (C=N); ^1H NMR ($CDCl_3$, 600 MHz) δ: 1.12-1.52 (m, 5H, all from CH_2), 1.58-1.79 (m, 3H, all from CH_2), 2.09-2.19 (m, 2H, all from CH_2), 3.77 (s, 1H, NH), 4.01-4.01 (m, 1H, CH), 5.04 (s, 1H, NH), 5.94 (d, J = 7.6Hz, 1H, CH), 6.57 (d, J = 7.2 Hz, 2H, ArH), 6.74 (d, J = 7.8 Hz, 2H, ArH), 6.90-6.93 (m, 2H, ArH), 7.09-7.24 (m, 4H, ArH), 7.28-7.30 (m, 4H, ArH); ^{13}C NMR ($CDCl_3$, 150.9 MHz) δ: 24.7, 24.9, 25.7, 32.4, 33.0, 48.5, 59.0, 113.7, 119.5, 121.6, 122.5, 128.5, 128.8, 129.2, 129.3, 134.1, 137.9, 146.8, 150.3, 154.6; HRMS (ESI): calcd for $C_{26}H_{29}ClN_3$ $[M+H]^+$ 418.20500; found 418.20388.

***N*-*tert*-Butyl-2-(furan-2-yl)-*N*'-phenyl-2-(phenylamino)acetimidamide (4g):**[1] White solid (312 mg, 90%); R_f (EtOAc/Hexane: 1/20) 0.56; ESI-MS (*m/z*): 348 $[M+H]^+$; IR (KBr) ν_{max}: 3296 (NH), 1635 (C=N); ^1H NMR ($CDCl_3$, 600 MHz) δ: 1.50 (s, 9H, $[CH_3]_3$), 4.03 (bs, 1H, NH), 5.12 (s, 1H, CH), 5.77 (s, 1H, NH), 6.33 (d, J = 3.6Hz, 1H, ArH), 6.40-6.41 (m, 1H, ArH), 6.72-6.76 (m, 4H, ArH), 6.89-6.94 (m, 2H, ArH), 7.18-7.21 (m, 2H, ArH), 7.27-7.30 (m, 2H, ArH), 7.39-7.40 (m, 1H, ArH); ^{13}C NMR ($CDCl_3$, 150 MHz) δ: 28.3, 50.9, 53.4, 108.8, 110.5, 113.9, 119.5, 121.4, 121.9, 128.6, 129.2, 142.3, 146.6, 150.3, 151.9, 152.1; HRMS (ESI): calcd for $C_{22}H_{26}N_3O$ $[M+H]^+$ 348.20759; found 348.20698.

***N*-*tert*-Butyl-2-cyclohexyl-*N*'-phenyl-2-(phenylamino)acetimidamide (4h):**[1] White solid (328 mg, 90%); R_f (EtOAc/Hexane: 1/20) 0.63; ESI-MS (*m/z*): 364 $[M+H]^+$; IR (KBr) ν_{max}: 3362 (NH), 1637 (C=N); ^1H NMR ($CDCl_3$, 600 MHz) δ: 0.93-0.96 (m, 1H, one hydrogen from CH_2), 1.00-1.15 (m, 4H, 2CH_2), 1.27-1.29 (m, 1H, one hydrogen from CH_2), 1.32-1.33 (m, 2H, CH_2), 1.44 (s, 9H, $[CH_3]_3$), 1.67-1.71 (m, 2H, CH_2), 1.79-1.90 (m, 3H, CH_2 & CH), 3.62 (bs, 1H, NH), 3.98 (s, 1H, CH), 5.48 (s, 1H, NH), 6.75-6.76 (m, 2H, ArH), 6.84-6.94 (m, 4H, ArH), 7.25-7.28 (m, 4H, ArH); ^{13}C NMR ($CDCl_3$, 150.9 MHz) δ: 26.2, 26.5, 26.6, 27.2, 28.4, 30.7, 41.0, 50.6, 59.4, 113.9, 118.8, 120.7, 122.0, 128.7, 129.2, 147.8, 151.0, 154.8; HRMS (ESI): calcd for $C_{24}H_{34}N_3$ $[M+H]^+$ 364.27527; found 364.27495.

***N-tert*-Butyl-*N*'-phenyl-2-(phenylamino)decanimidamide (4i):** White solid (343 mg, 87%); R_f (EtOAc/Hexane: 1/20) 0.69; ESI-MS (*m/z*): 394 $[M+H]^+$; IR (KBr) ν_{max}: 3298 (NH), 1630 (C=N); ^{1}H NMR ($CDCl_3$, 600 MHz) δ: 0.94 (t, J = 7.2 Hz, 3H, CH_3), 1.10-1.36 (m, 12H, 6CH_2), 1.42 (s, 9H, $[CH_3]_3$), 1.54-1.62 (m, 1H, one hydrogen from CH_2), 1.75-1.82 (m, 1H, one hydrogen from CH_2), 3.65 (bs, 1H, NH), 3.98-4.00 (m, 1H, CH), 5.51 (s, 1H, NH), 6.73 (d, J = 8.4 Hz, 2H, ArH), 6.84-6.94 (m, 4H, ArH), 7.25-7.29 (m, 4H, ArH); ^{13}C NMR ($CDCl_3$, 150 MHz) δ: 14.1, 22.6, 25.7, 28.4, 28.9, 29.1, 29.2, 31.8, 34.5, 50.5, 54.5, 113.8, 118.9, 120.9, 122.1, 128.8, 129.2, 147.5, 151.1, 156.5; HRMS (ESI): calcd for $C_{26}H_{40}N_3$ $[M+H]^+$ 394.32222; found 394.32156.

***N-tert*-Butyl-2-(4-fluorophenyl)-*N*'-phenyl-2-(phenylamino)acetimidamide (4j):** White solid (330 mg, 88%); R_f (EtOAc/Hexane: 1/20) 0.56; ESI-MS (*m/z*): 376 $[M+H]^+$; IR (KBr) ν_{max}: 3276 (NH), 1628 (C=N); ^{1}H NMR ($CDCl_3$, 600 MHz) δ: 1.45 (s, 9H, $[CH_3]_3$), 3.65 (bs, 1H, NH), 4.91-4.93 (m, 1H, CH), 5.87 (s, 1H, NH), 6.47-6.50 (m, 2H, ArH), 6.67-6.70 (m, 2H, ArH), 6.81-6.85 (m, 2H, ArH), 6.93-6.96 (m, 2H, ArH), 7.02-7.10 (m, 4H, ArH), 7.21-7.23 (m, 2H, ArH); ^{13}C NMR ($CDCl_3$, 150 MHz) δ: 28.5, 51.0, 59.2, 113.9, 115.6, 115.8, 119.6, 121.3, 122.3, 128.6, 129.4, 129.9, 129.9, 129.9, 135.8, 147.0, 150.6, 150.6, 153.8, 161.8, 163.4; HRMS (ESI): calcd for $C_{24}H_{27}FN_3$ $[M+H]^+$ 376.21890; found 376.21821.

***N-tert*-Butyl-2-(4-chlorophenyl)-*N*'-phenyl-2-(phenylamino)acetimidamide (4k):** White solid (333 mg, 85%); R_f (EtOAc/Hexane: 1/20) 0.55; ESI-MS (*m/z*): 392 $[M+H]^+$; IR (KBr) ν_{max}: 3319 (NH), 1636 (C=N); ^{1}H NMR ($CDCl_3$, 600 MHz) δ: 1.44 (s, 9H, $[CH_3]_3$), 3.65 (bs, 1H, NH), 4.92 (s, 1H, CH), 5.84 (s, 1H, NH), 6.48 (d, J = 7.8Hz, 2H, ArH), 6.68 (d, J = 8.4 Hz, 2H, ArH), 6.82-6.87 (m, 2H, ArH), 7.04-7.07 (m, 4H, ArH), 7.22-7.25 (m, 4H, ArH); ^{13}C NMR ($CDCl_3$, 150 MHz) δ: 28.4, 51.0, 59.2, 113.9, 119.6, 121.3, 122.2, 128.6, 128.9, 129.4, 129.5, 134.2, 138.3, 146.9, 150.5, 153.5; HRMS (ESI): calcd for $C_{24}H_{27}ClN_3$ $[M+H]^+$ 392.18935; found 392.18843.

***N-tert*-Butyl-2-(4-chlorophenyl)-*N*'-(4-methoxyphenyl)-2-(4-methoxyphenylamino)acetimidamide (4l):**[3] White solid (407 mg, 90%); R_f (EtOAc/Hexane: 1/20) 0.51; ESI-MS (*m/z*): 452 $[M+H]^+$; IR (KBr) ν_{max}: 3319 (NH), 1630 (C=N); ^{1}H NMR ($CDCl_3$, 600 MHz) δ: 1.48 (s, 9H, $[CH_3]_3$), 3.48 (s, 1H, NH), 3.76 (s, 3H, OCH_3), 3.81 (s, 3H, OCH_3), 4.89 (s, 1H, CH), 5.95 (s, 1H, NH), 6.42 (d, J = 9.0 Hz, 2H, ArH), 6.66-6.68 (m, 4H, ArH), 6.85 (d, J = 9.0 Hz, 2H, ArH), 7.10 (d, J = 8.4 Hz, 2H, ArH), 7.28 (d, J = 8.4 Hz, 2H, ArH); ^{13}C NMR ($CDCl_3$, 150.9 MHz) δ: 28.4, 50.9, 55.6, 55.7, 59.7, 114.0, 114.8, 115.0, 122.9, 128.9, 129.4, 134.0, 138.5, 141.0, 143.8, 153.4, 154.5; HRMS (ESI): calcd for $C_{26}H_{31}ClN_3O_2$ $[M+H]^+$ 452.21048; found 452.21001.

***N-tert*-Butyl-2-phenyl-*N*'-p-tolyl-2-(*p*-tolylamino)acetimidamide (4m):** White solid (332 mg, 86%); R_f (EtOAc/Hexane: 1/20) 0.56; ESI-MS (*m/z*): 386 $[M+H]^+$; IR (KBr) ν_{max}: 3350 (NH), 1630 (C=N); ^{1}H NMR ($CDCl_3$, 600 MHz) δ: 1.51 (s, 9H, $[CH_3]_3$), 2.26 (s, 3H, CH_3), 2.32 (s, 3H, CH_3), 3.66 (bs, 1H, NH), 4.95 (s, 1H, CH), 5.94 (s, 1H, NH), 6.42 (d, J = 8.4 Hz, 2H, ArH), 6.65 (d, J = 8.4 Hz, 2H, ArH), 6.89 (d, J = 8.4 Hz, 2H, ArH), 7.08 (d, J = 8.4 Hz, 2H, ArH), 7.21-7.23 (m, 2H, ArH), 7.31-7.34 (m, 3H, ArH); ^{13}C NMR ($CDCl_3$, 150.9 MHz) δ: 20.5, 20.7, 28.4, 50.8, 60.1, 113.9, 122.0, 128.0, 128.1, 128.6, 128.7, 129.0, 129.7, 130.2, 140.2, 145.0, 148.0, 154.2; HRMS (ESI): calcd for $C_{26}H_{32}N_3$ $[M+H]^+$ 386.25962; found 386.25811.

***N-tert*-Butyl-2-(4-fluorophenyl)-*N*'-*p*-tolyl-2-(p-tolylamino)acetimidamide (4n):** White solid (372 mg, 92%); R_f (EtOAc/Hexane: 1/20) 0.55; ESI-MS (*m/z*): 404 $[M+H]^+$; IR (KBr) ν_{max}: 3285 (NH), 1634 (C=N); ^{1}H NMR ($CDCl_3$, 600 MHz) δ: 1.45 (s, 9H, $[CH_3]_3$), 2.25 (s, 3H, CH_3), 2.31 (s, 3H, CH_3), 3.55

(bs, 1H, NH), 4.92 (s, 1H, CH), 5.91 (s, 1H, NH), 6.39 (d, J = 7.8 Hz, 2H, ArH), 6.63 (d, J = 8.4 Hz, 2H, ArH), 6.88 (d, J = 8.4 Hz, 2H, ArH), 6.98-7.00 (m, 2H, ArH), 7.07 (d, J = 7.8 Hz, 2H, ArH), 7.15-7.17 (m, 2H, ArH); ^{13}C NMR ($CDCl_3$, 150 MHz) δ: 20.4, 20.7, 28.4, 50.8, 59.3, 113.9, 115.5, 115.6, 120.8, 122.0, 128.7, 129.0, 129.8, 130.3, 136.0, 144.8, 147.9, 154.1, 161.7, 163.3; HRMS (ESI): calcd for $C_{26}H_{31}FN_3$ $[M+H]^+$ 404.25020; found 404.24910.

***N-tert*-Butyl-*N*'-*p*-tolyl-2-(*p*-tolylamino)pentanimidamide (4o):** White solid (357 mg, 91%); R_f (EtOAc/Hexane: 1/20) 0.70; ESI-MS (*m/z*): 352 $[M+H]^+$; IR (KBr) ν_{max}: 3311 (NH), 1635 (C=N); ^{1}H NMR ($CDCl_3$, 600 MHz) δ: 0.77 (t, J = 7.2Hz, 3H, CH_3), 1.22-1.25 (m, 1H, one hydrogen from CH_2), 1.39-1.44 (m, 10H, one hydrogen from CH_2 & $[CH_3]_3$), 1.57-1.60 (m, 1H, one hydrogen from CH_2), 1.75-1.77 (m, 1H, one hydrogen from CH_2), 2.33 (s, 6H, $2CH_3$), 3.50 (bs, 1H, NH), 3.97-3.99 (m, 1H, CH), 5.56 (s, 1H, NH), 6.66 (d, J = 8.4Hz, 2H, ArH), 6.80 (d, J = 7.8Hz, 2H, ArH), 7.07 (d, J = 8.4 Hz, 4H, ArH); ^{13}C NMR ($CDCl_3$, 150.9 MHz) δ: 13.6, 19.0, 20.4, 20.7, 28.5, 36.9, 50.4, 54.5, 113.9, 122.0, 128.1, 129.4, 129.7, 130.0, 145.3, 148.4, 157.0; HRMS (ESI): calcd for $C_{23}H_{34}N_3$ $[M+H]^+$ 352.27527; found 352.27411.

***N-tert*-Butyl-2-cyclohexyl-*N*'-*p*-tolyl-2-(*p*-tolylamino)acetimidamide (4p):** White solid (325 mg, 83%); R_f (EtOAc/Hexane: 1/20) 0.60; ESI-MS (*m/z*): 392 $[M+H]^+$; IR (KBr) ν_{max}: 3290(NH), 1634 (C=N); ^{1}H NMR ($CDCl_3$, 600 MHz) δ: 1.05-1.17 (m, 4H, $2CH_2$), 1.36-1.37 (m, 2H, CH_2), 1.45 (s, 9H, $[CH_3]_3$), 1.71-1.91 (m, 5H, $2CH_2$ & CH), 2.34 (s, 6H, $2CH_3$), 3.51 (bs, 1H, NH), 3.95 (d, J = 3.6Hz, 1H, CH), 5.54 (s, 1H, NH), 6.69 (d, J = 7.8Hz, 2H, ArH), 6.79 (d, J = 7.2Hz, 2H, ArH), 7.07-7.10 (m, 4H, ArH); ^{13}C NMR ($CDCl_3$, 150.9 MHz) δ: 20.5, 20.8, 26.3, 26.6, 26.7, 27.2, 28.5, 30.7, 41.1, 50.6, 59.8, 114.1, 121.9, 128.0, 129.3, 129.7, 145.8, 148.4, 155.3; HRMS (ESI): calcd for $C_{26}H_{38}N_3$ $[M+H]^+$ 392.30657; found 392.30532.

***N-tert*-Butyl-*N*'-phenyl-2-(phenylamino)-2-(2-(phenylethynyl)phenyl)acetimidamide (4q):** White solid (389 mg, 85%); R_f (EtOAc/Hexane: 2/3) 0.55; ESI-MS (*m/z*): 458 $[M+H]^+$; IR (KBr) ν_{max}: 3281 (NH), 2227, 1631, 1609; ^{1}H NMR ($CDCl_3$, 500 MHz) δ: 1.52 (s, 9H, $[CH_3]_3$), 3.72 (bs, 1H, NH), 5.46 (s, 1H, CH), 6.11 (bs, 1H, NH), 6.44 (d, J = 8.2 Hz, 2H, ArH), 6.66-6.74 (m, 5H, ArH), 6.83 (t, J = 7.3Hz, 1H, ArH), 6.90 (t, J = 7.4Hz, 2H, ArH), 7.07 (t, J = 7.4Hz, 2H, ArH), 7.12-7.17 (m, 1H, ArH), 7.19 (t, J = 7.4Hz, 2H, ArH), 7.28 (dt, J = 1.2, 7.4Hz, 1H, ArH), 7.33 (dt, J = 1.4, 7.6Hz, 1H, ArH), 7.37 (dd, J = 1.2, 7.6Hz, 1H, ArH), 7.62 (d, J = 7.8Hz, 1H, ArH); ^{13}C NMR ($CDCl_3$, 125.8 MHz) δ: 28.4, 50.9, 58.5, 85.9, 94.5, 113.9, 115.0, 119.3, 121.1, 121.8, 122.5, 123.7, 127.4, 127.8, 127.9, 128.0, 128.4, 129.2, 131.3, 132.0, 141.5, 148.3, 150.5, 154.3; HRMS (ESI): calcd for $C_{32}H_{32}N_3$ $[M+H]^+$ 458.25962; found 458.25858.

***N-tert*-Butyl-2-(3-methyl-1-phenyl-5-(phenylethynyl)-1*H*-pyrazol-4-yl)-*N*'-phenyl-2-(phenylamino)acetimidamide (4r):** White solid (414 mg, 77%); R_f (EtOAc/Hexane: 2/3) 0.51; ESI-MS (*m/z*): 538 $[M+H]^+$; IR (KBr) ν_{max}: 3375 (NH), 1951, 1631, 1591; ^{1}H NMR ($CDCl_3$, 300 MHz) δ: 1.42 (s, 9H, $[CH_3]_3$), 2.13 (s, 3H, CH_3), 3.80 (bs, 1H, NH), 5.24 (s, 1H, CH), 6.16 (bs, 1H, NH), 6.58 (d, J = 8.3 Hz, 2H, ArH), 6.76 (d, J = 7.6 Hz, 2H, ArH), 6.85 (t, J = 7.6 Hz, 1H, ArH), 7.02 (t, J = 7.6 Hz, 2H, ArH), 7.10 (d, J = 7.6 Hz, 2H, ArH), 7.25-7.30 (m, 5H, ArH), 7.34 (d, J = 6.8 Hz, 1H, ArH), 7.42-7.47 (m, 3H, ArH), 7.66 (d, J = 7.6 Hz, 2H, ArH); ^{13}C NMR ($CDCl_3$, 75.5 MHz) δ: 12.3, 28.2, 51.9, 53.9, 99.5, 113.8, 113.9, 115.1, 119.4, 119.6, 121.0, 121.6, 121.7, 122.7, 123.2, 125.1, 127.4, 128.3, 128.7, 128.9, 129.1, 129.3, 131.3, 147.5, 148.0, 152.5; HRMS (ESI): calcd for $C_{36}H_{36}N_5$ $[M+H]^+$ 538.29707; found 538.29565.

Ethyl 2-(*N*'-phenyl-2-(phenylamino)-2-(3,4,5-trimethoxyphenyl)acetimidamido)acetate (4s): White solid (357 mg, 75%); R_f (EtOAc/Hexane: 4/1) 0.40; ESI-MS (*m/z*): 478 $[M+H]^+$; IR (KBr) ν_{max}: 3400 (NH), 1745, 1633, 1591; ^1H NMR ($CDCl_3$, 300 MHz) δ: 1.26 (t, J = 6.8 Hz, 3H, CH_3), 3.76 (s, 6H, $2OCH_3$), 3.84 (s, 3H, OCH_3), 3.89-3.98 (m, 3H, CH_2 + NH), 4.16 (q, J = 6.8 Hz, 2H, OCH_2), 4.41-4.47 (bs, 1H, NH), 4.93 (s, 1H, CH), 6.42 (s, 2H, ArH), 6.53 (d, J = 7.6 Hz, 2H, ArH), 6.69 (d, J = 8.3 Hz, 2H, ArH), 6.83-6.91 (m, 2H, ArH), 7.07 (t, J = 8.3 Hz, 2H, ArH), 7.22-7.27 (m, 2H, ArH); ^{13}C NMR ($CDCl_3$, 75.5 MHz) δ: 14.2, 42.7, 56.1, 60.0, 60.6, 61.0, 105.0, 113.8, 119.5, 121.9, 122.4, 128.5, 129.4, 134.5, 137.8, 147.0, 149.7, 153.3, 156.4, 170.7; HRMS (ESI): calcd for $C_{27}H_{32}N_3O_5$ $[M+H]^+$ 478.23420; found 478.23266.

***N*'-Phenyl-2-(phenylamino)-*N*-(tosylmethyl)-2-(3,4,5-trimethoxyphenyl)acetimidamide (4t):** White solid (442 mg, 79%); R_f (EtOAc/Hexane: 4/1) 0.33; ESI-MS (*m/z*): 560 $[M+H]^+$; IR (KBr) ν_{max}: 3377 (NH), 1651, 1593, 1508; ^1H NMR ($CDCl_3$, 300 MHz) δ: 2.40 (s, 3H, CH_3), 3.78 (s, 6H, $2OCH_3$), 3.87 (s, 3H, OCH_3), 3.91 (s, 2H, CH_2), 4.51-4.65 (m, 1H, NH), 4.84 (s, 1H, CH), 5.33-5.41 (m, 1H, NH), 6.20 (d, J = 7.6 Hz, 2H, ArH), 6.32 (s, 2H, ArH), 6.63 (d, J = 7.7 Hz, 2H, ArH), 6.86-6.92 (m, 3H, ArH), 7.06 (t, J = 7.6 Hz, 2H, ArH), 7.18-7.27 (m, 3H, ArH), 7.67 (d, J = 8.1 Hz, 2H, ArH); ^{13}C NMR ($CDCl_3$, 125.8 MHz) δ: 21.6, 56.2, 59.5, 60.8, 61.6, 104.8, 113.9, 119.8, 121.6, 122.2, 128.5, 128.6, 129.4, 129.5, 133.7, 135.5, 137.9, 144.7, 146.6, 148.5, 153.3, 154.5; HRMS (ESI): calcd for $C_{31}H_{34}N_3O_5S$ $[M+H]^+$ 560.22192; found 560.22070.

***N-tert*-Butyl-2-(3-methoxyphenyl)imidazo[1,2-*a*]pyridin-3-amine (7a):** White solid (242 mg, 82%); R_f (EtOAc/Hexane: 1/1) 0.55; ESI-MS (*m/z*): 296 $[M+H]^+$; IR (KBr) ν_{max}: 3354 (NH), 1632, 1590; ^1H NMR ($CDCl_3$, 300 MHz) δ: 0.99 (s, 9H $[CH_3]_3$), 3.82 (s, 3H OCH_3), 6.64 (t, J = 6.8 Hz, 1H, ArH), 6.80 (dd, J = 2.2, 8.1 Hz, 1H, ArH), 7.01 (t, J = 6.8 Hz, 1H, ArH), 7.23 (t, J = 8.1 Hz, 1H, ArH), 7.48-7.55 (m, 3H, ArH), 8.13 (d, J = 6.8 Hz, 1H, ArH); ^{13}C NMR ($CDCl_3$, 75.5 MHz) δ: 29.9, 54.8, 55.9, 111.0, 113.0, 113.2, 116.6, 120.3, 123.1, 123.3, 123.8, 128.7, 135.9, 138.6, 141.4, 159.2; HRMS (ESI): calcd for $C_{18}H_{22}N_3O$ $[M+H]^+$ 296.17629; found 296.17520.

***N-tert*-Butyl-2-(3,5-dimethoxyphenyl)imidazo[1,2-*a*]pyridin-3-amine (7b):** White solid (276 mg, 85%); R_f (EtOAc/Hexane: 1/1) 0.33; ESI-MS (*m/z*): 326 $[M+H]^+$; IR (KBr) ν_{max}: 3351 (NH), 1609, 1592; ^1H NMR ($CDCl_3$, 500 MHz) δ: 1.06 (s, 9H $[CH_3]_3$), 3.86 (s, 6H $2OCH_3$), 6.40-6.44 (m, 1H, ArH), 6.76 (t, J = 6.8 Hz, 1H, ArH), 7.10 (d, J = 2.1Hz, 2H, ArH), 7.11-7.14 (m, 1H, ArH), 7.55 (d, J = 9.0 Hz, 1H, ArH), 8.21 (d, J = 6.7 Hz, 1H, ArH); ^{13}C NMR ($CDCl_3$, 75.5 MHz) δ: 30.2, 55.4, 56.4, 100.0, 106.2, 111.4, 117.1, 123.4, 123.6, 124.2, 136.8, 138.9, 148.7, 160.6; HRMS (ESI): calcd for $C_{19}H_{24}N_3O_2$ $[M+H]^+$ 326.18685; found 326.18605.

***N-tert*-Butyl-2-(3,4,5-trimethoxyphenyl)imidazo[1,2-*a*]pyridin-3-amine (7c):** White solid (294 mg, 85%); R_f (EtOAc/Hexane: 1/1) 0.25; ESI-MS (*m/z*): 356 $[M+H]^+$; IR (KBr) ν_{max}: 3360 (NH), 1634, 1603; ^1H NMR ($CDCl_3$, 500 MHz) δ: 1.09 (s, 9H $[CH_3]_3$), 3.05 (bs, 1H, NH), 3.89 (s, 3H OCH_3), 3.95 (s, 6H $2OCH_3$), 6.77-6.80 (m, 1H, ArH), 7.15 (t, J = 6.8 Hz, 1H, ArH), 7.26 (s, 2H, ArH), 7.54 (d, J = 9.0 Hz, 1H, ArH), 8.17 (d, J = 7.6 Hz, 1H, ArH); ^{13}C NMR ($CDCl_3$, 75.5 MHz) δ: 30.4, 56.2, 56.3, 60.9, 105.5, 111.4, 117.2, 123.1, 123.2, 124.0, 130.7, 137.6, 139.3, 141.8, 153.0; HRMS (ESI): calcd for $C_{20}H_{26}N_3O_3$ $[M+H]^+$ 356.19742; found 356.19604.

4-(3-(*tert*-Butylamino)imidazo[1,2-*a*]pyridin-2-yl)benzonitrile (7d): White solid (246 mg, 85%); R_f (EtOAc/Hexane: 2/3) 0.48; ESI-MS (*m/z*): 291 $[M+H]^+$; IR (KBr) ν_{max}: 3358 (NH), 2215 (CN), 1638, 1500; ^{1}H NMR ($CDCl_3$, 500 MHz) δ: 1.07 (s, 9H $[CH_3]_3$), 3.05 (bs, 1H, NH), 6.80 (t, J = 7.5 Hz, 1H, ArH), 7.17 (dd, J = 1.0, 6.8 Hz, 1H, ArH), 7.54 (d, J = 9.1 Hz, 1H, ArH), 7.68 (d, J = 8.5 Hz, 2H, ArH), 8.17-8.20 (m, 3H, ArH); ^{13}C NMR ($CDCl_3$, 75.5 MHz) δ: 30.4, 56.7, 110.5, 111.9, 117.6, 119.1, 123.3, 124.4, 124.8, 128.3, 131.9, 137.4, 139.8, 142.3; HRMS (ESI): calcd for $C_{18}H_{19}N_4$ $[M+H]^+$ 291.16097; found 291.16032.

References:

1. Sharma, S.; Maurya, R. A.; Min, K.-I.; Jeong, G.-Y.; Kim, D.-P. *Angew. Chem. Int. Ed*. **2013**, *52*, 7564-7568.
2. Khan, A. T.; Basha R, S.; Lal, M.; Mir, M. H. *RSC Adv*, **2012**, *2*, 5506–5509.
3. Kumar, A.; Saxena, D.; Gupta, M. K. *Green Chem*. **2013**, 15, 2699-2703.

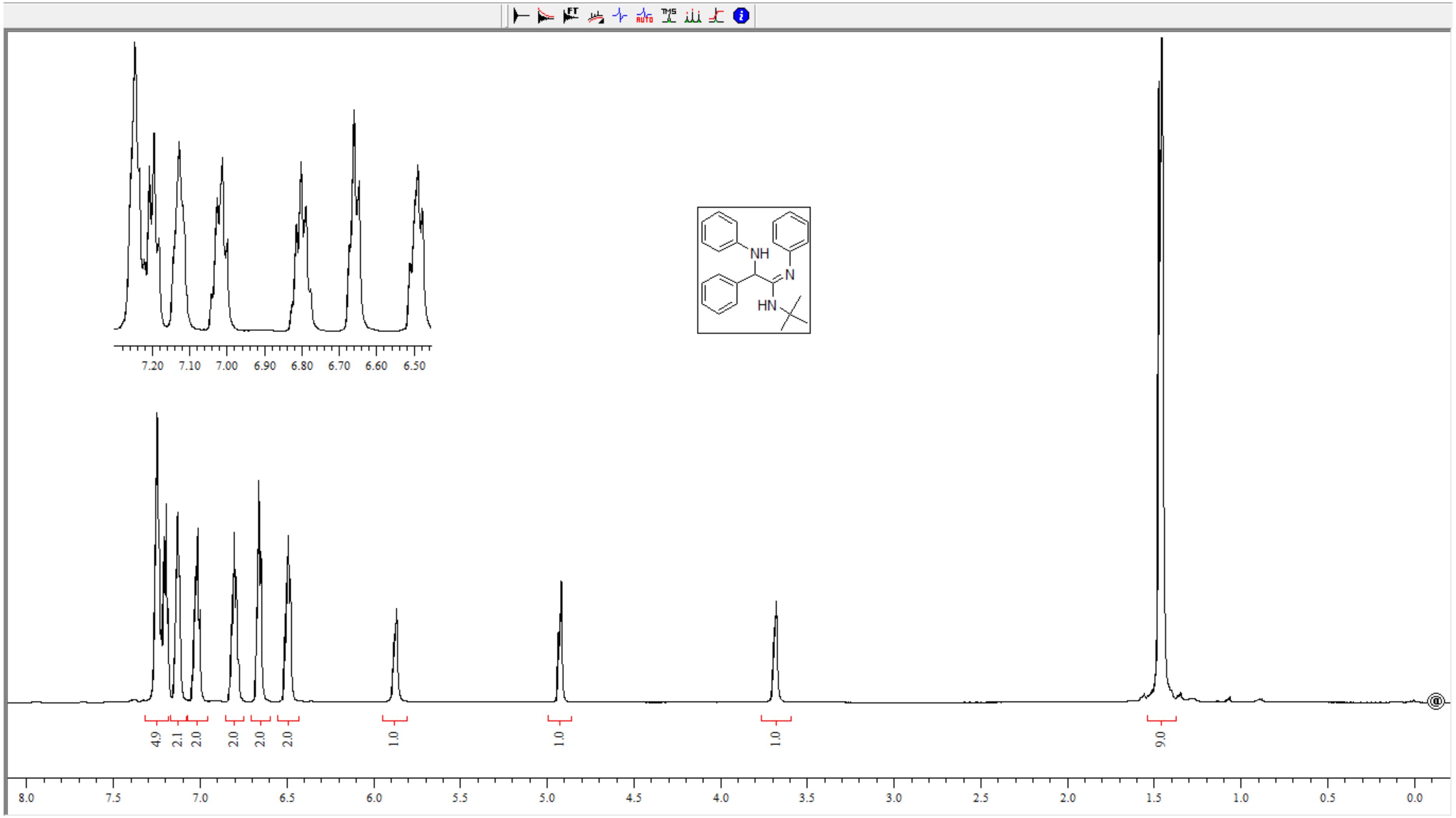

^{1}H NMR of compound **4a**

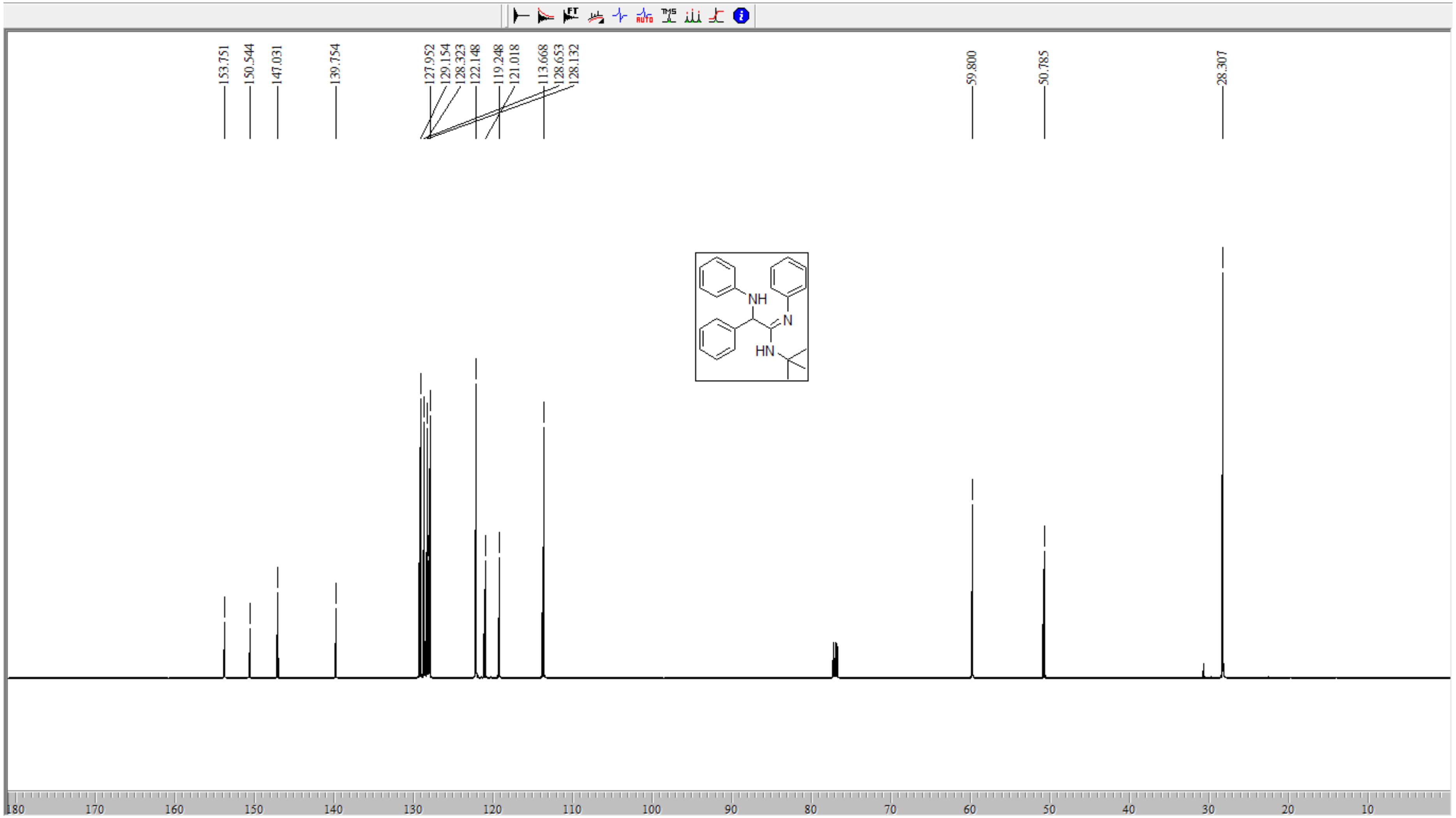

^{13}C NMR of compound **4a**

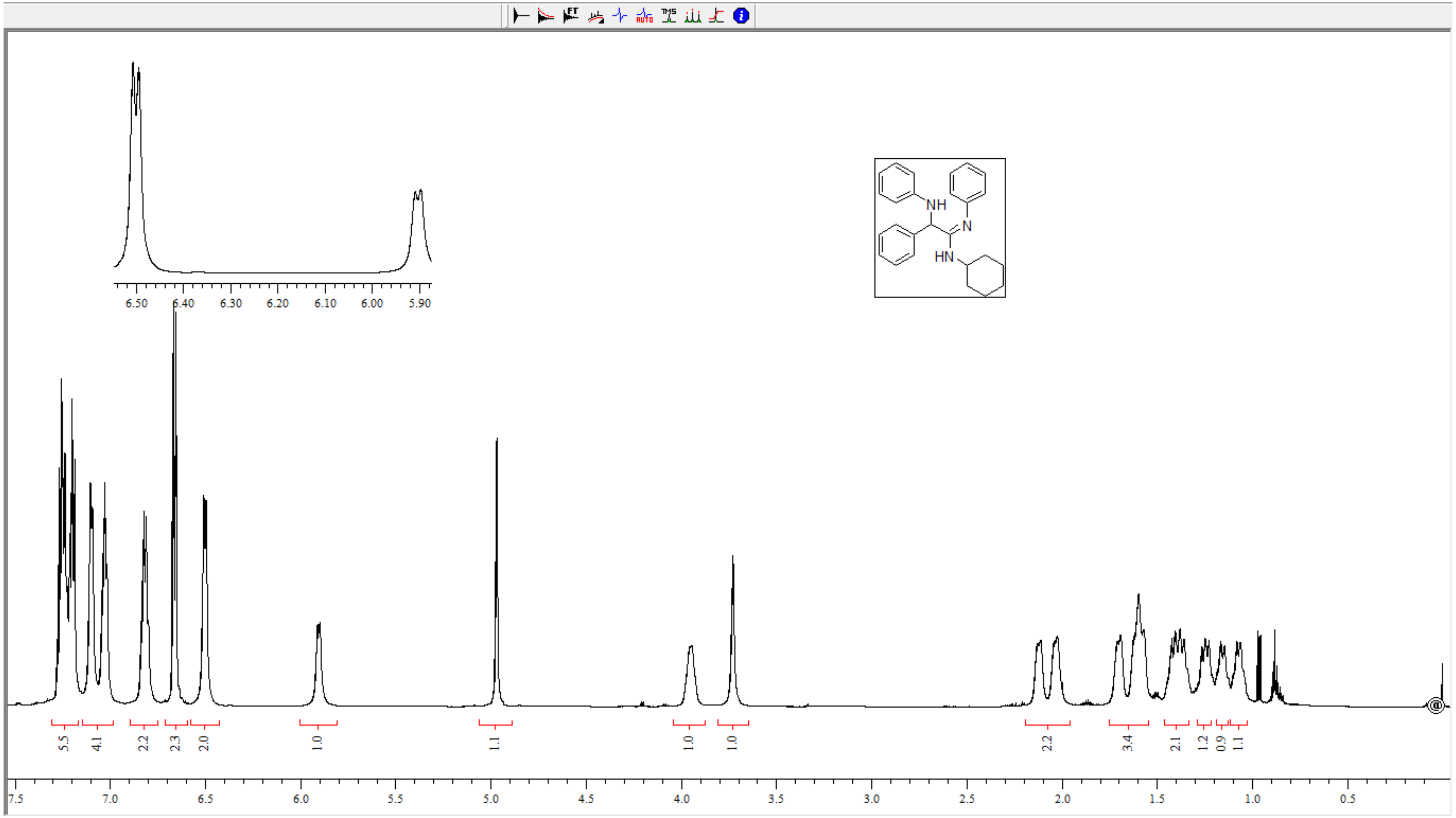

^{1}H NMR of compound **4b**

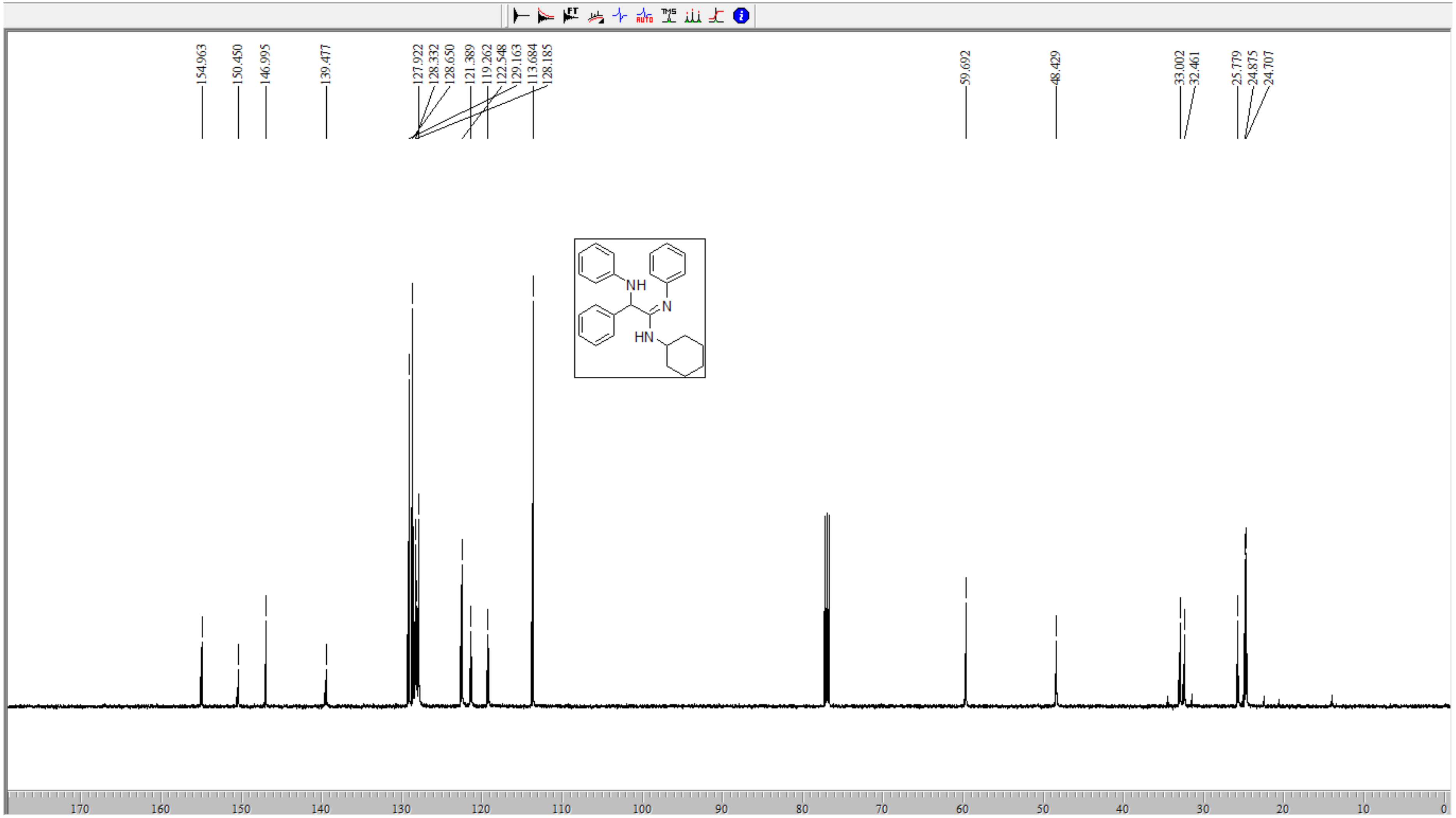

^{13}C NMR of compound **4b**

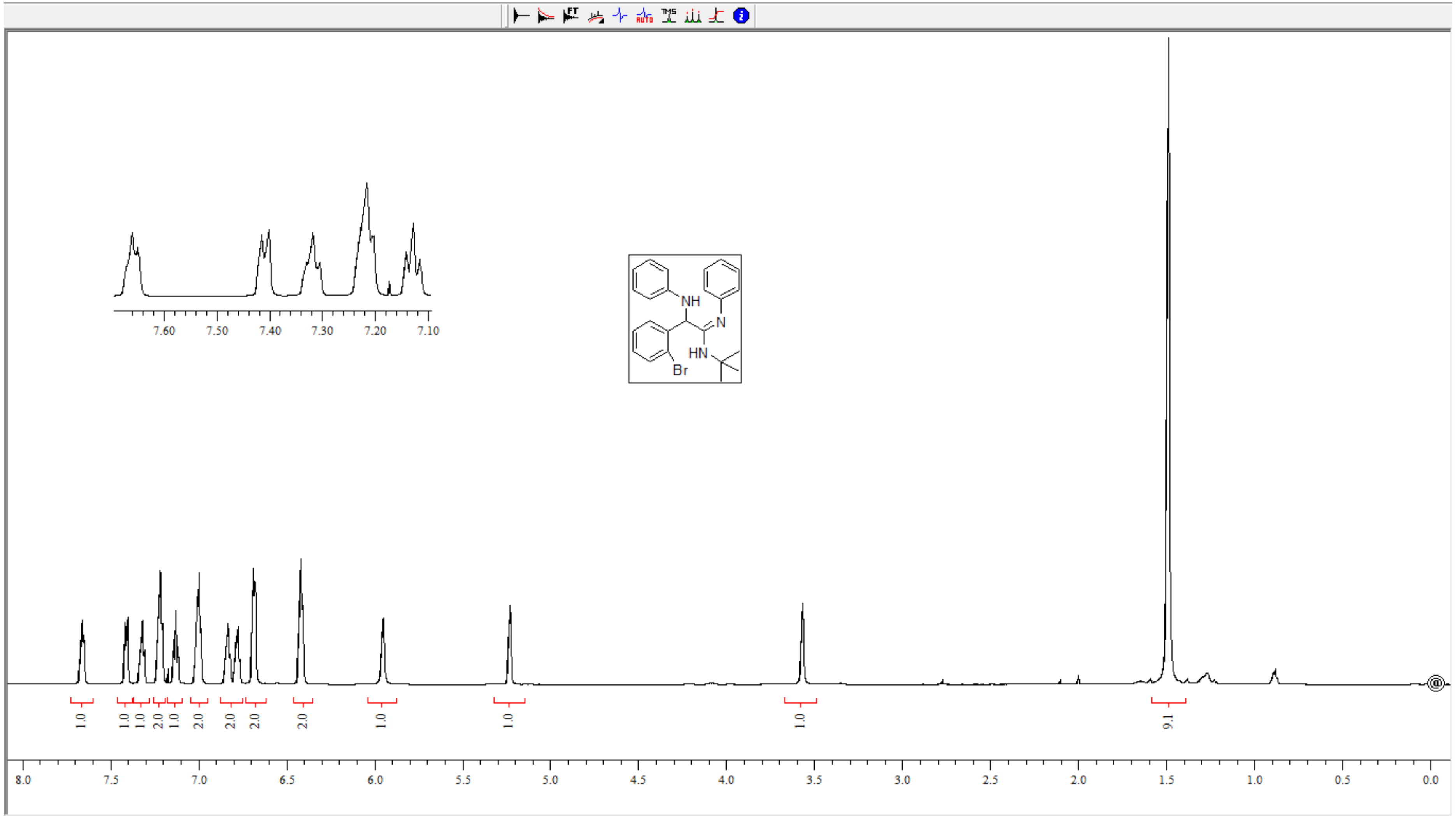

^{1}H NMR of compound **4c**

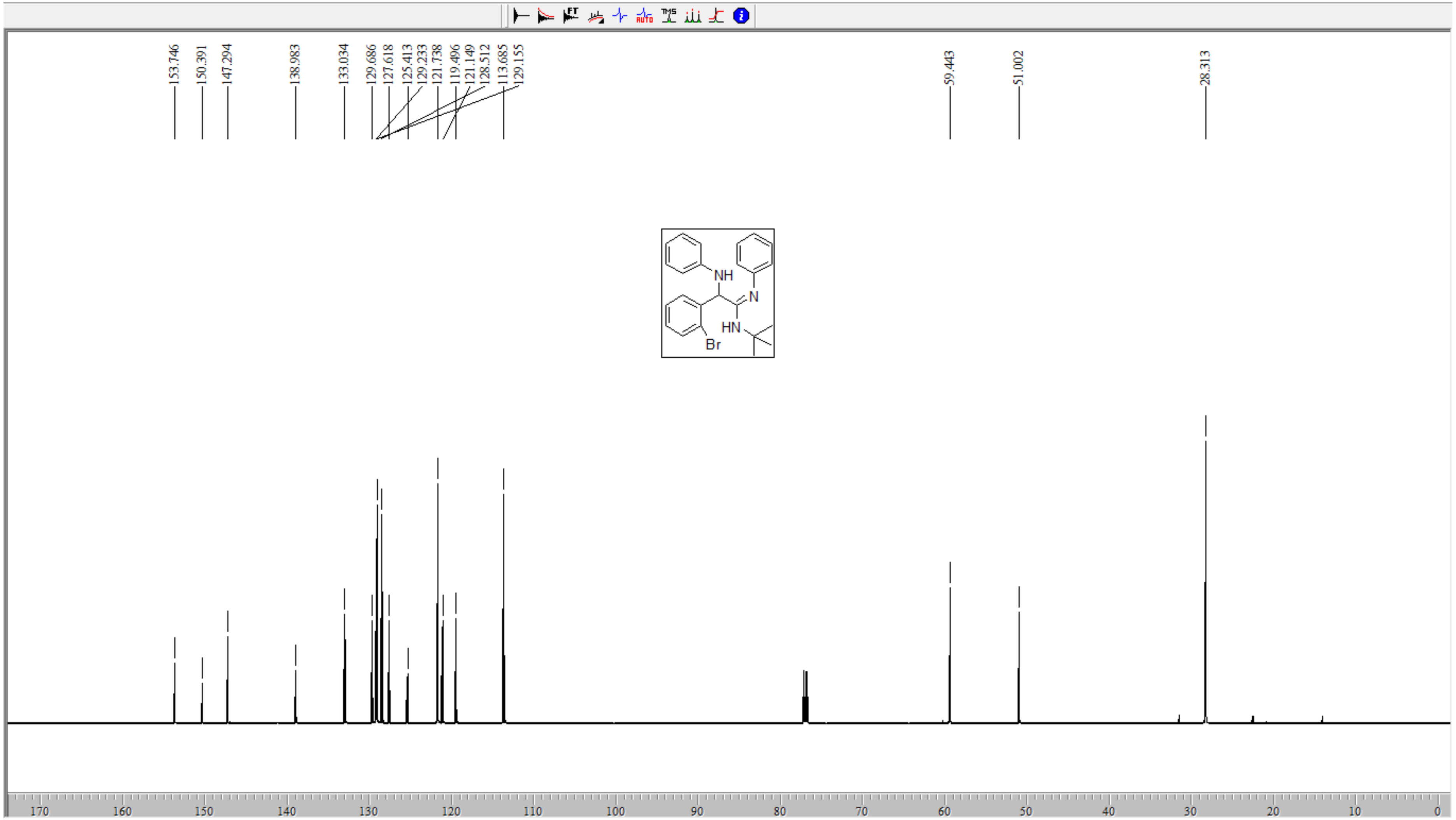

^{13}C NMR of compound **4c**

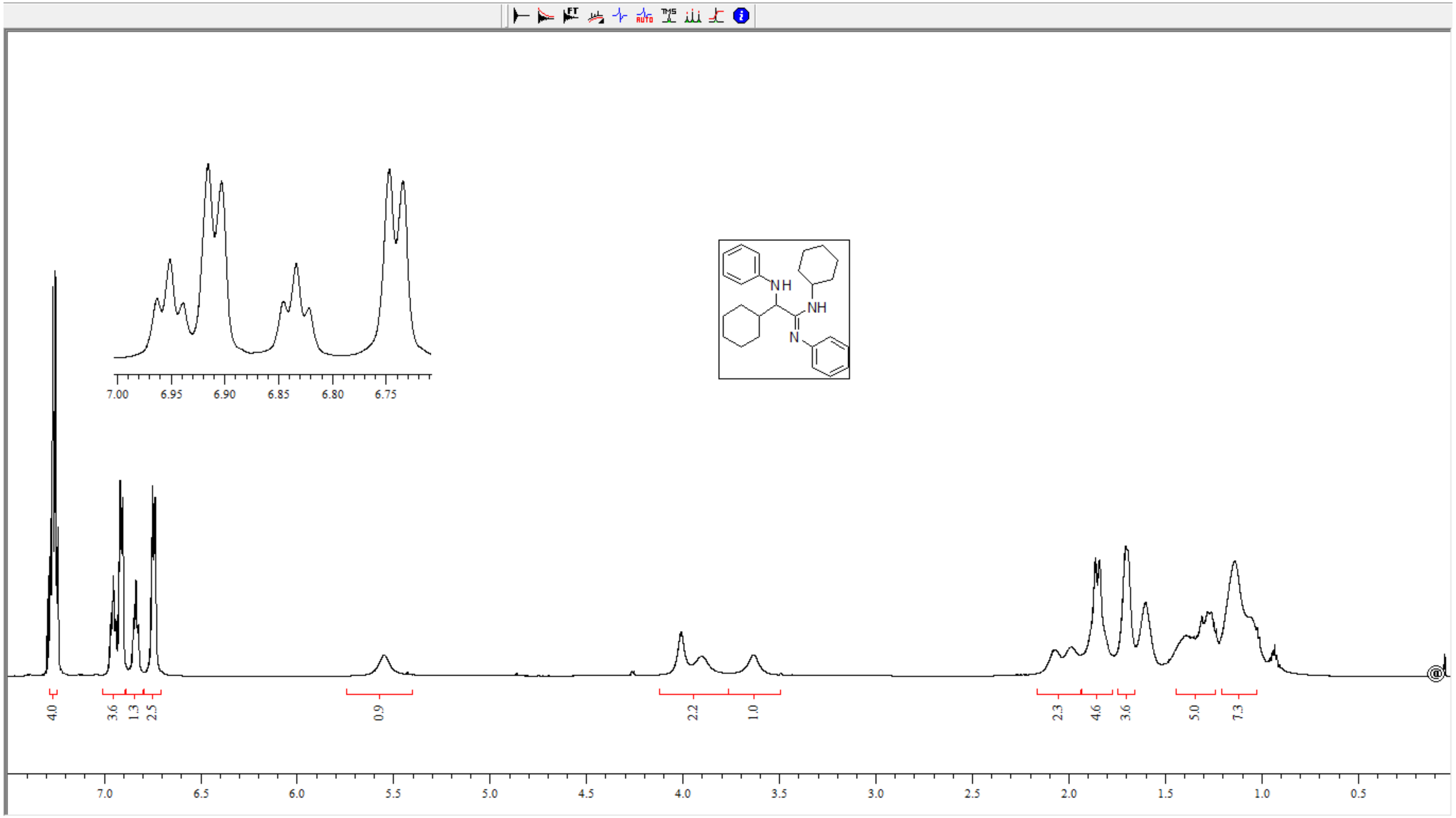

^{1}H NMR of compound **4d**

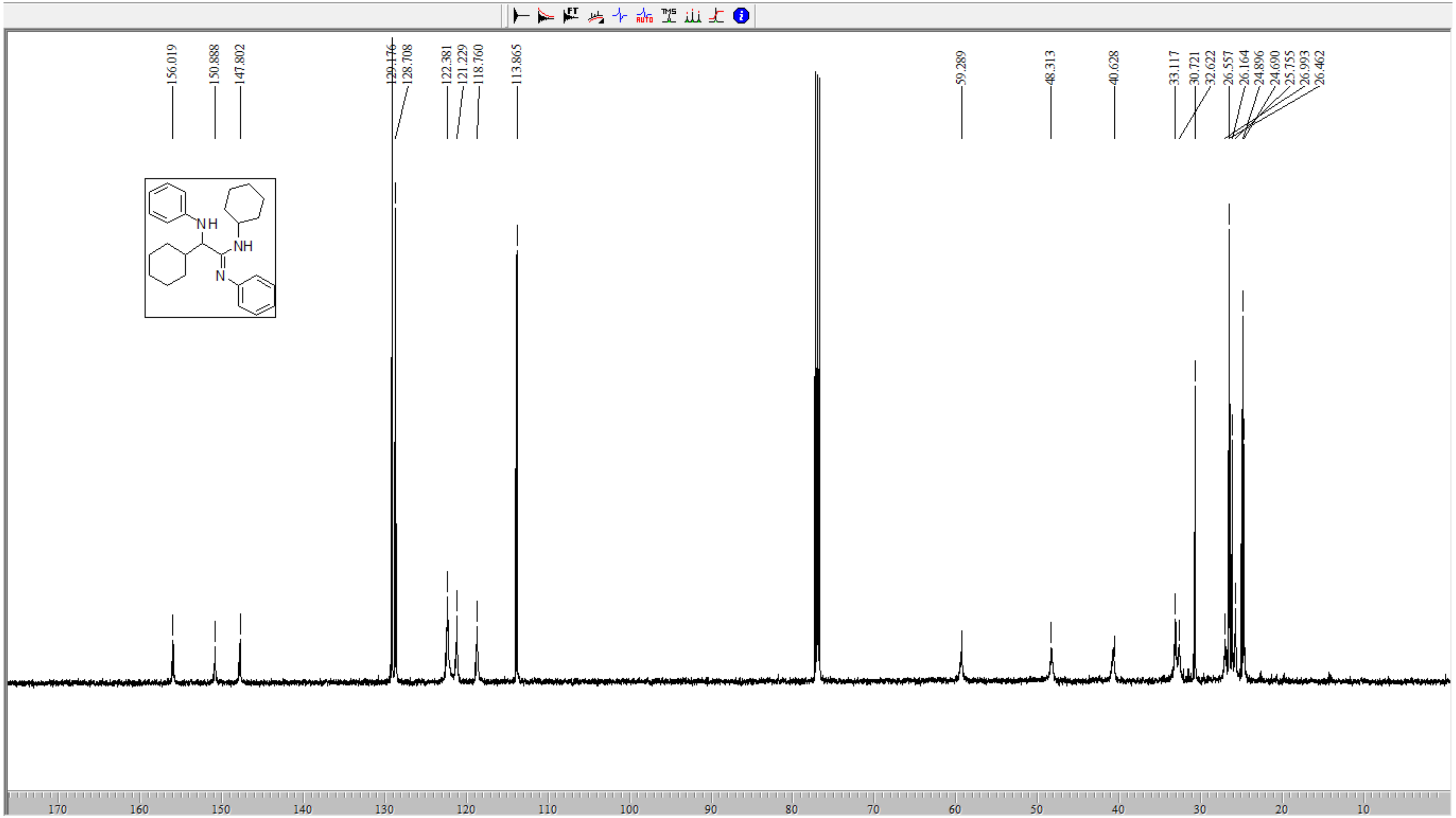

^{13}C NMR of compound **4d**

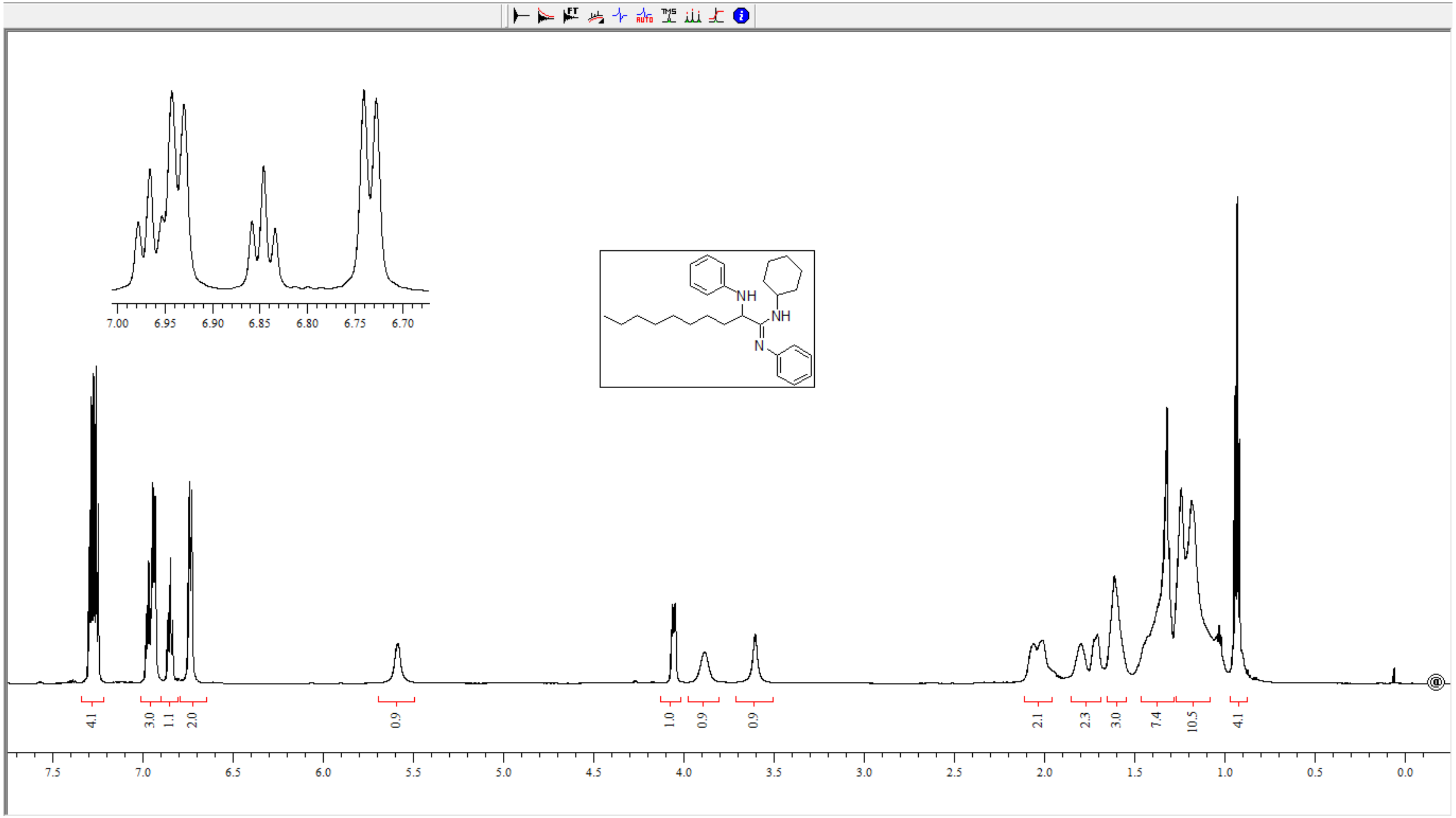

^{1}H NMR of compound **4e**

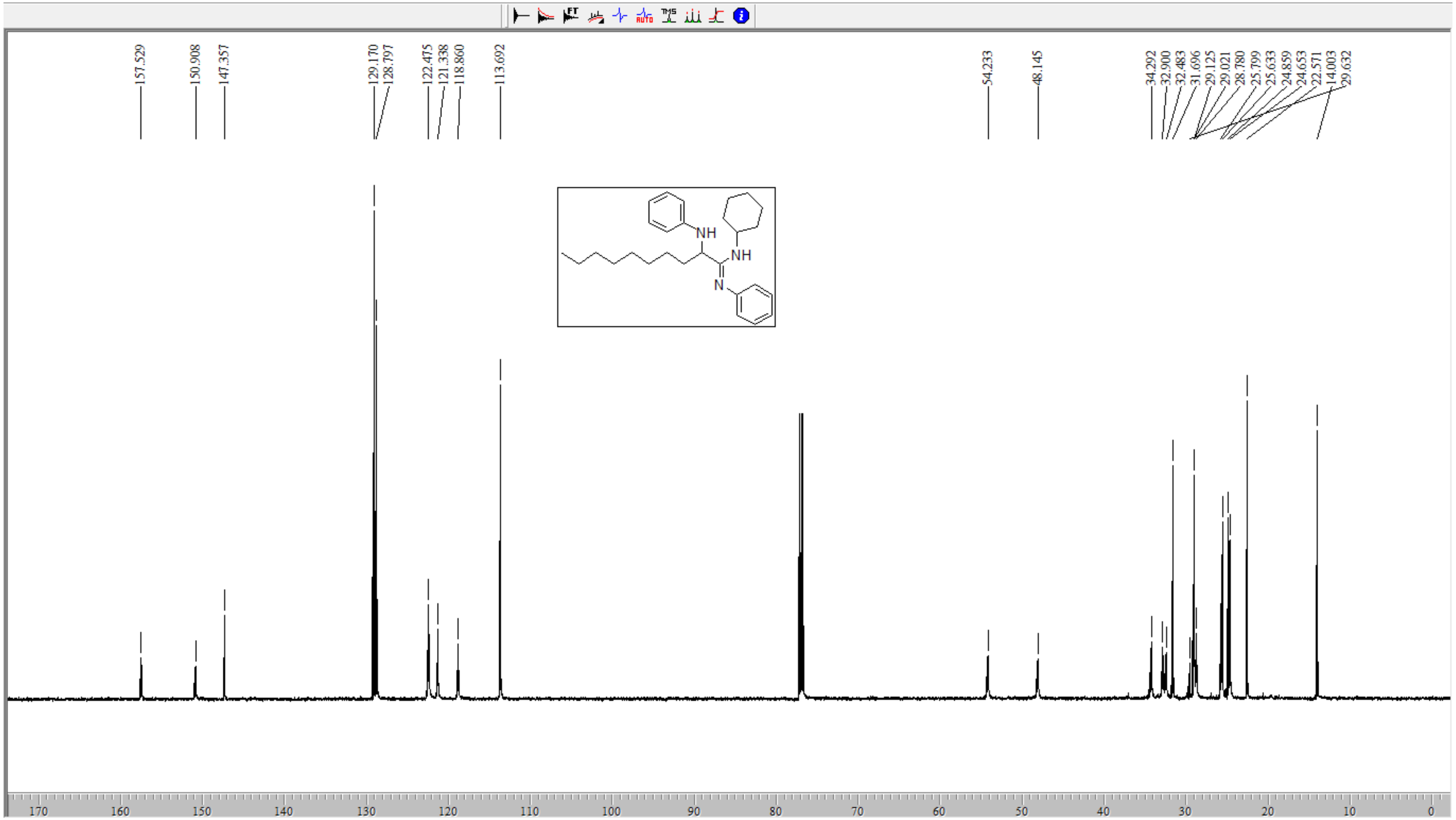

^{13}C NMR of compound **4e**

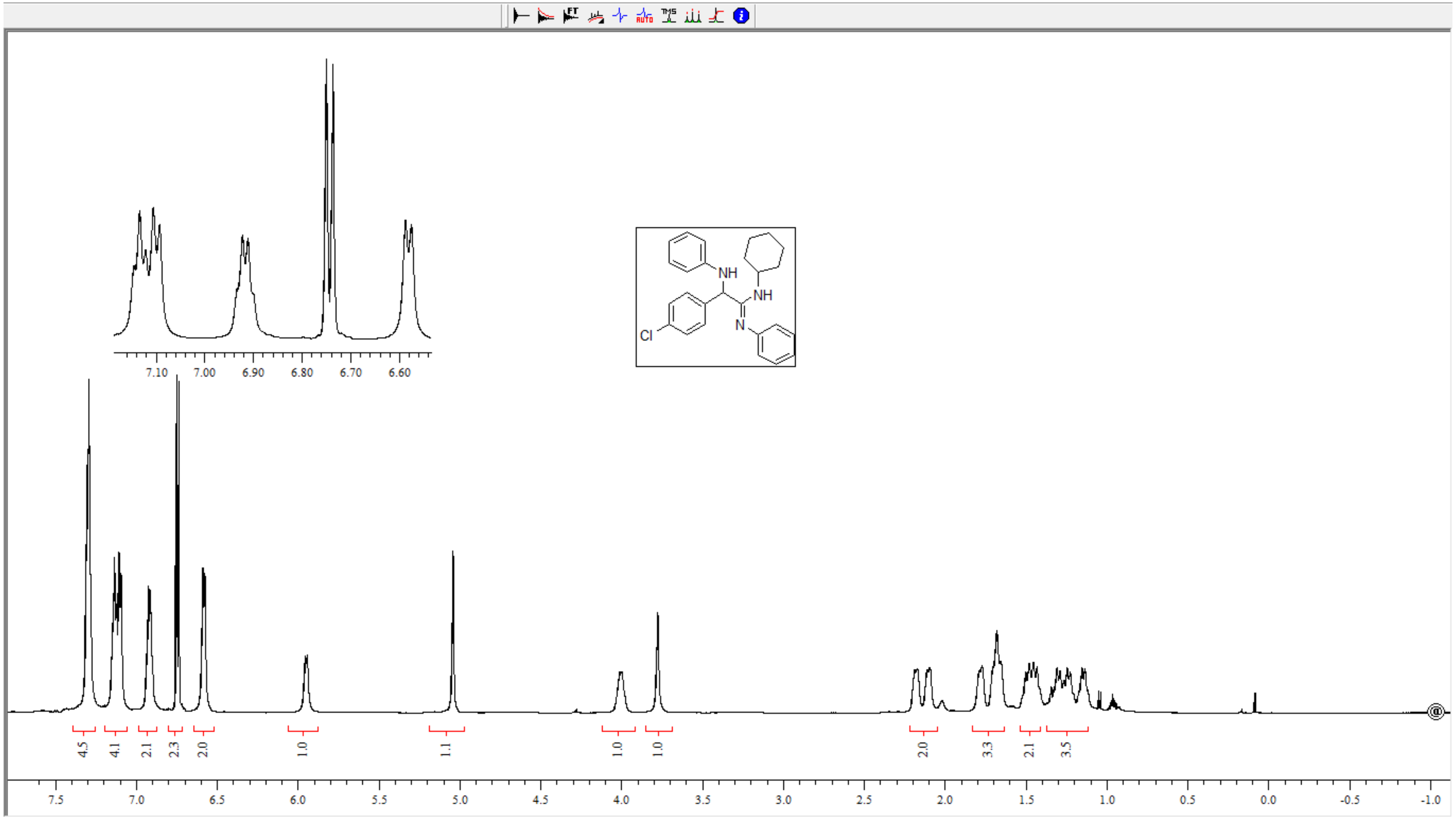

^{1}H NMR of compound **4f**

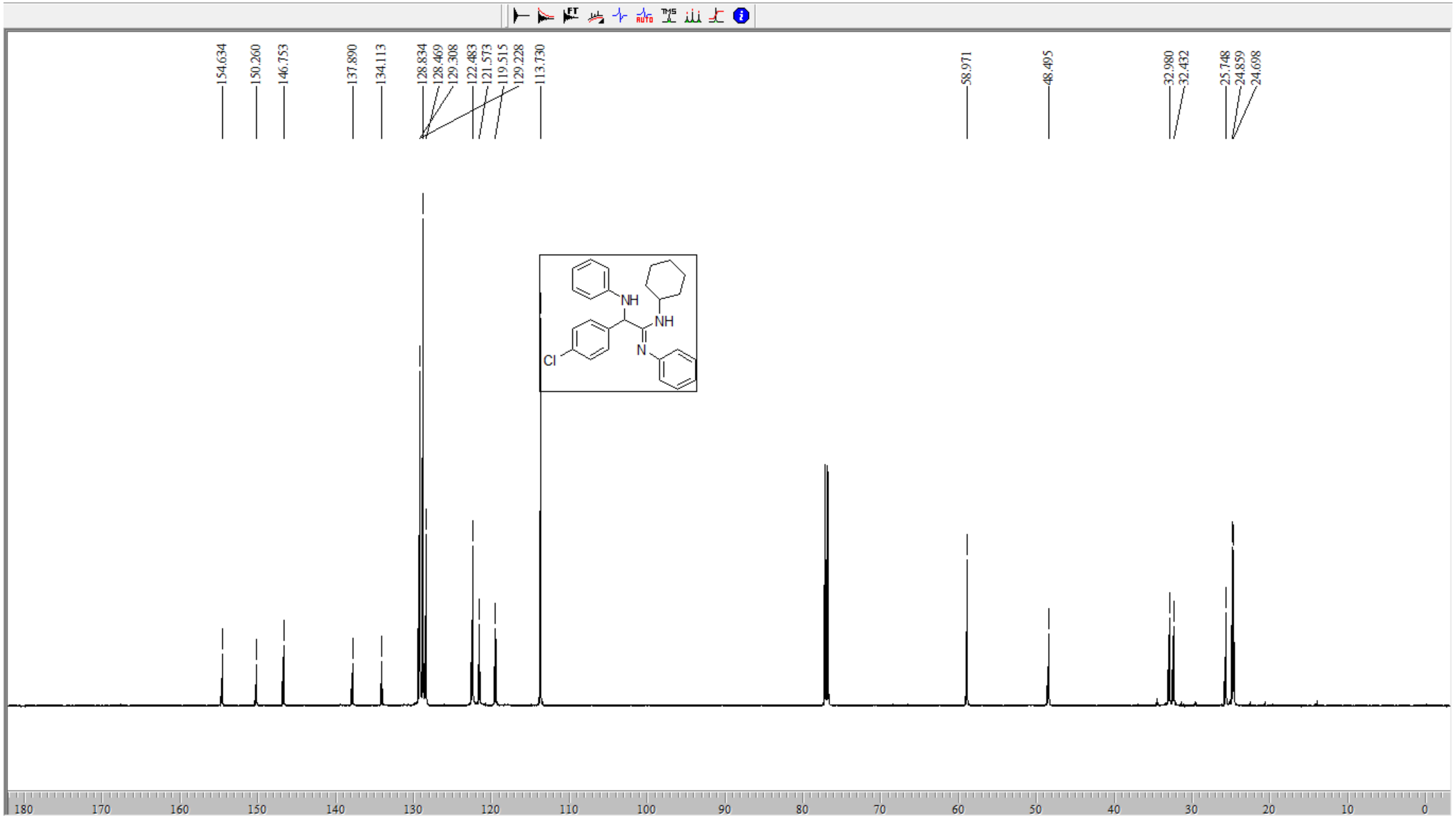

^{13}C NMR of compound **4f**

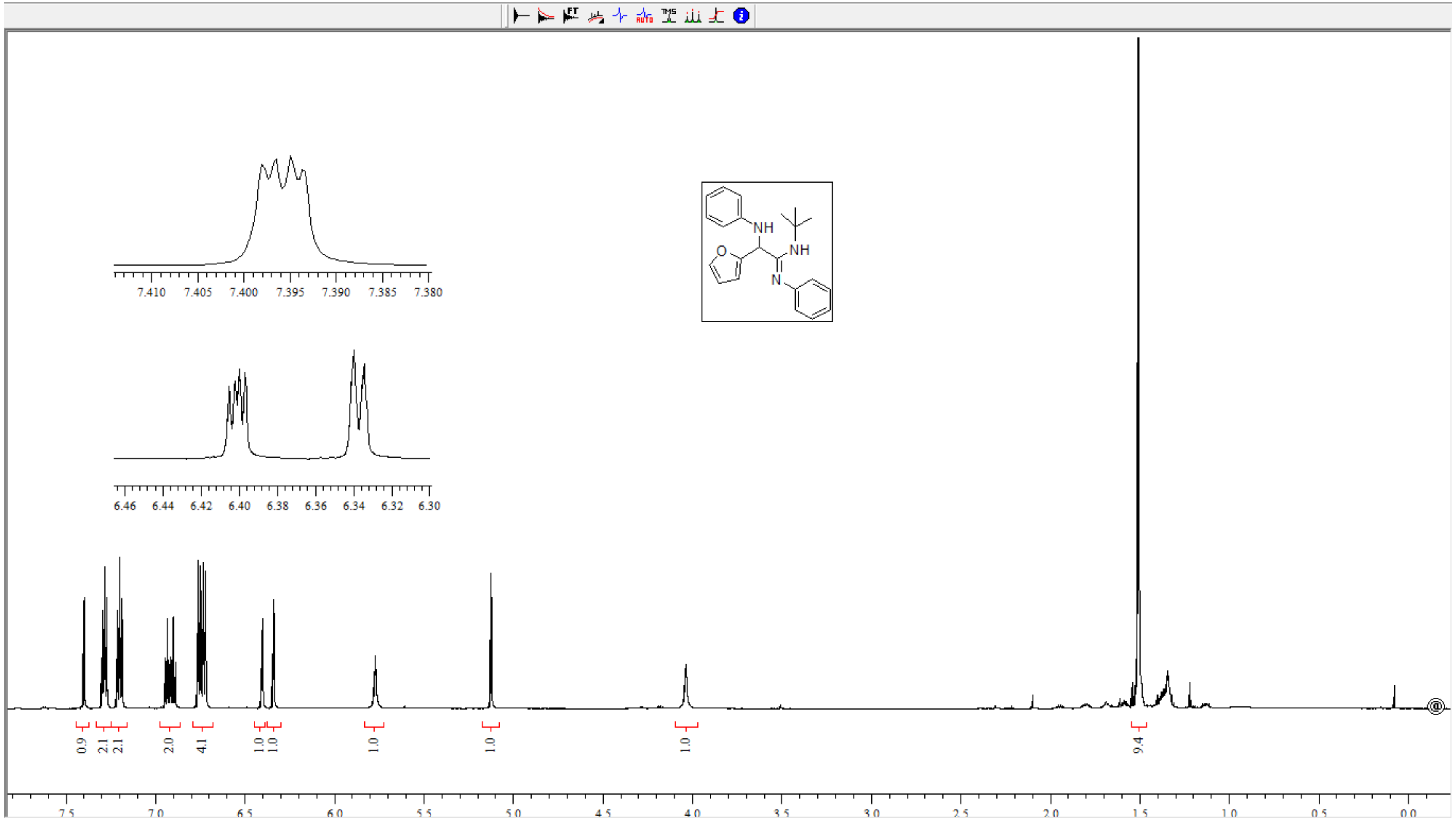

^{1}H NMR of compound **4g**

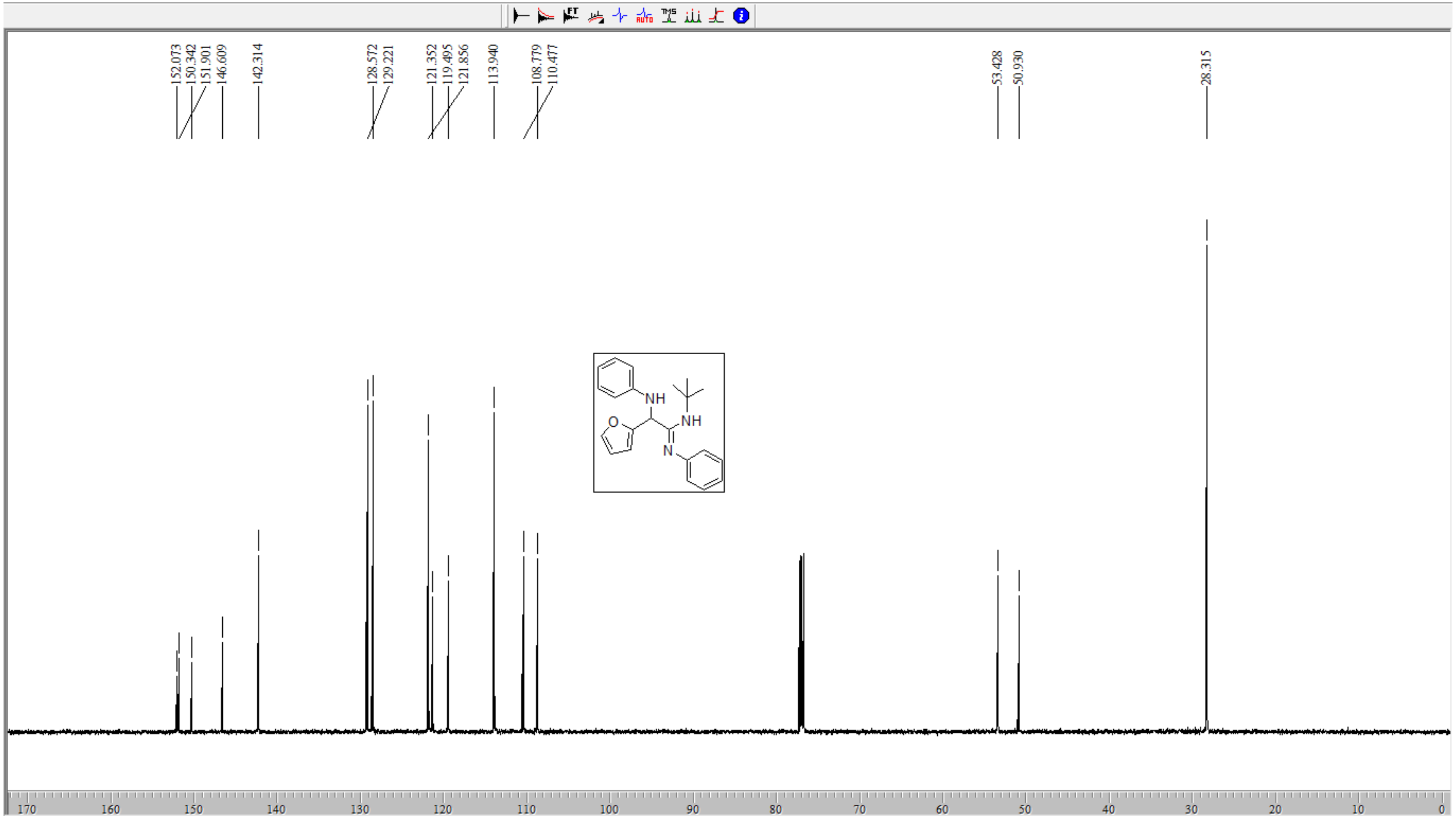

^{13}C NMR of compound **4g**

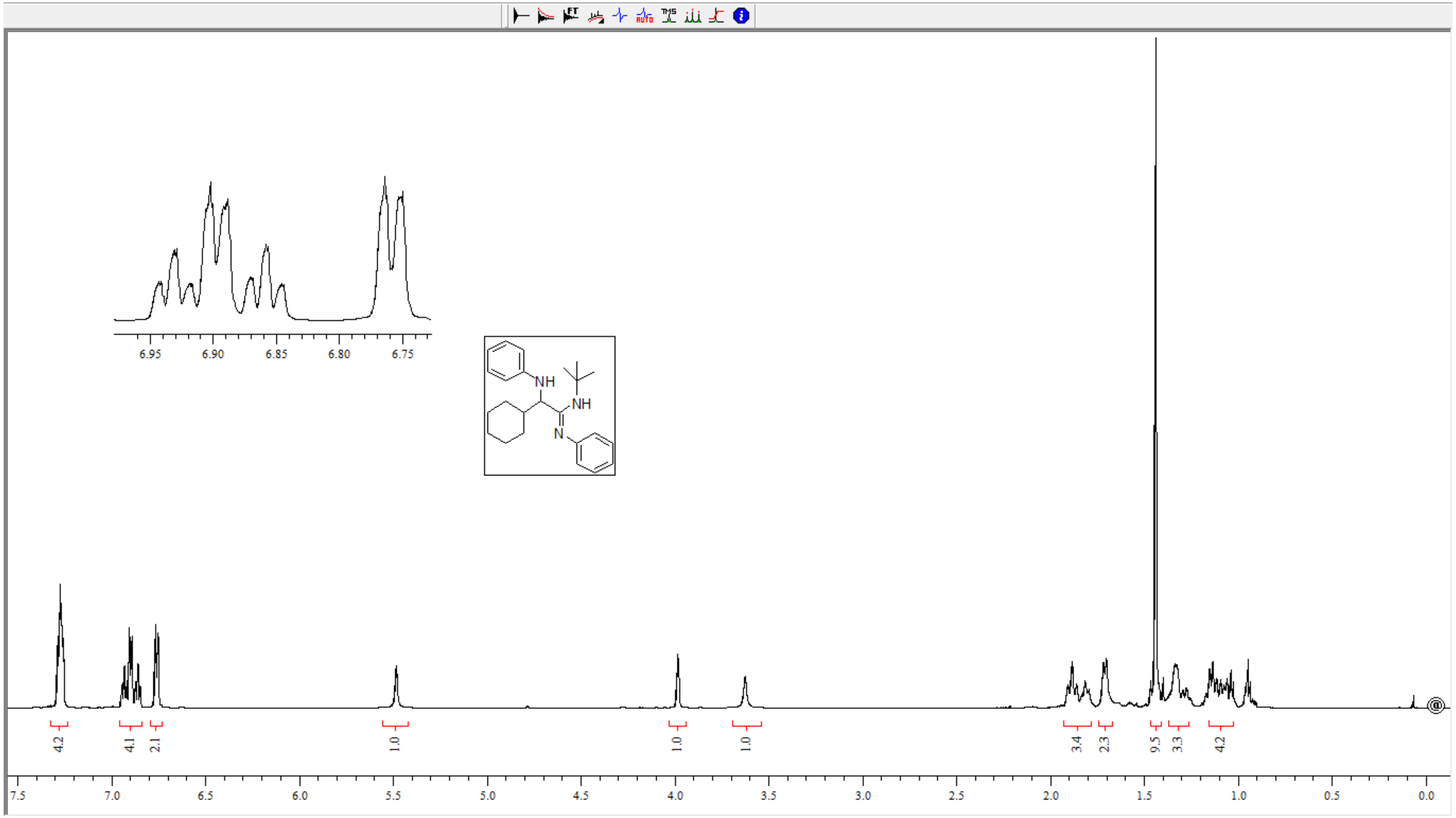

^{1}H NMR of compound **4h**

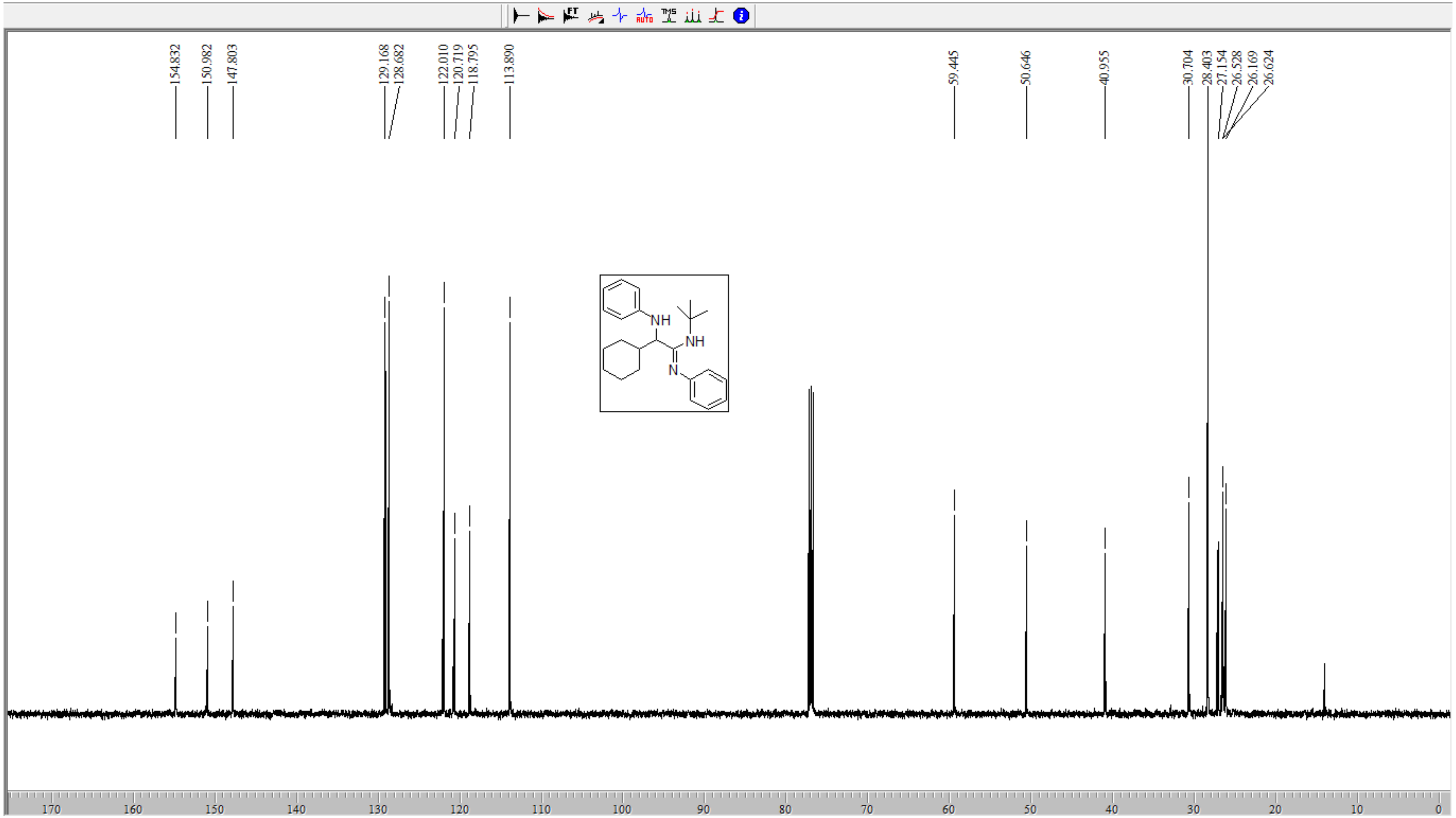

^{13}C NMR of compound **4h**

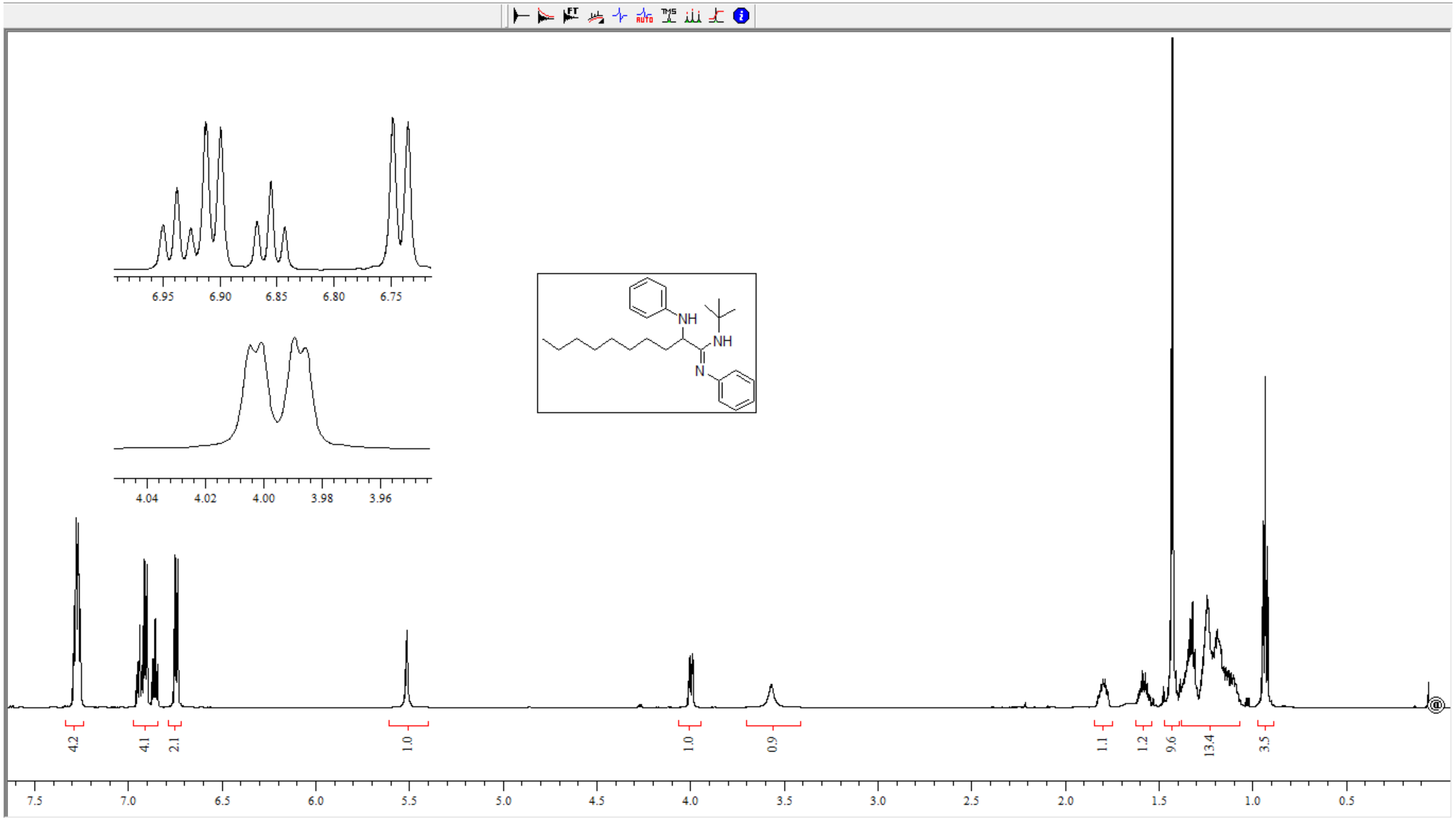

^{1}H NMR of compound **4i**

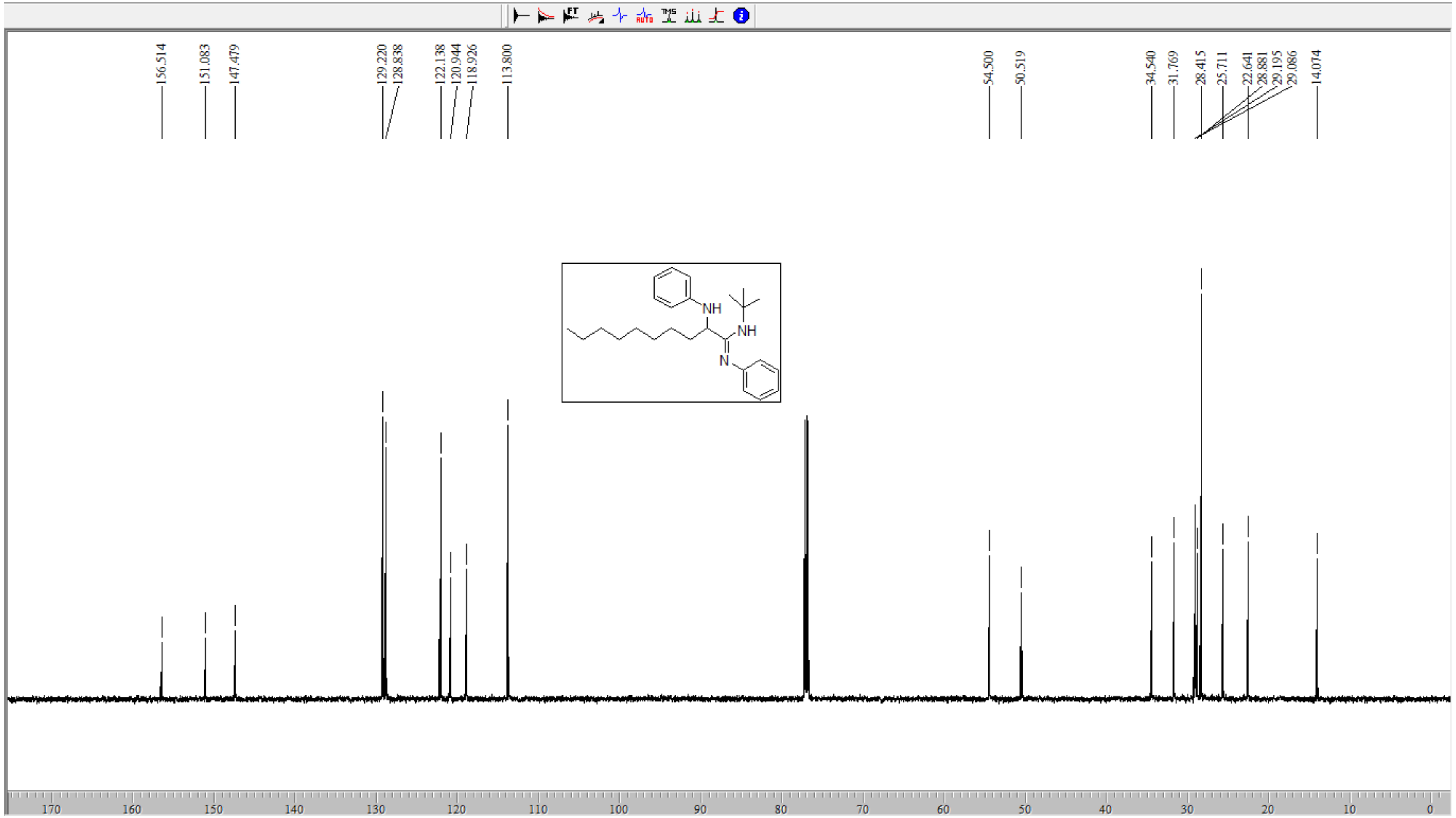

^{13}C NMR of compound **4i**

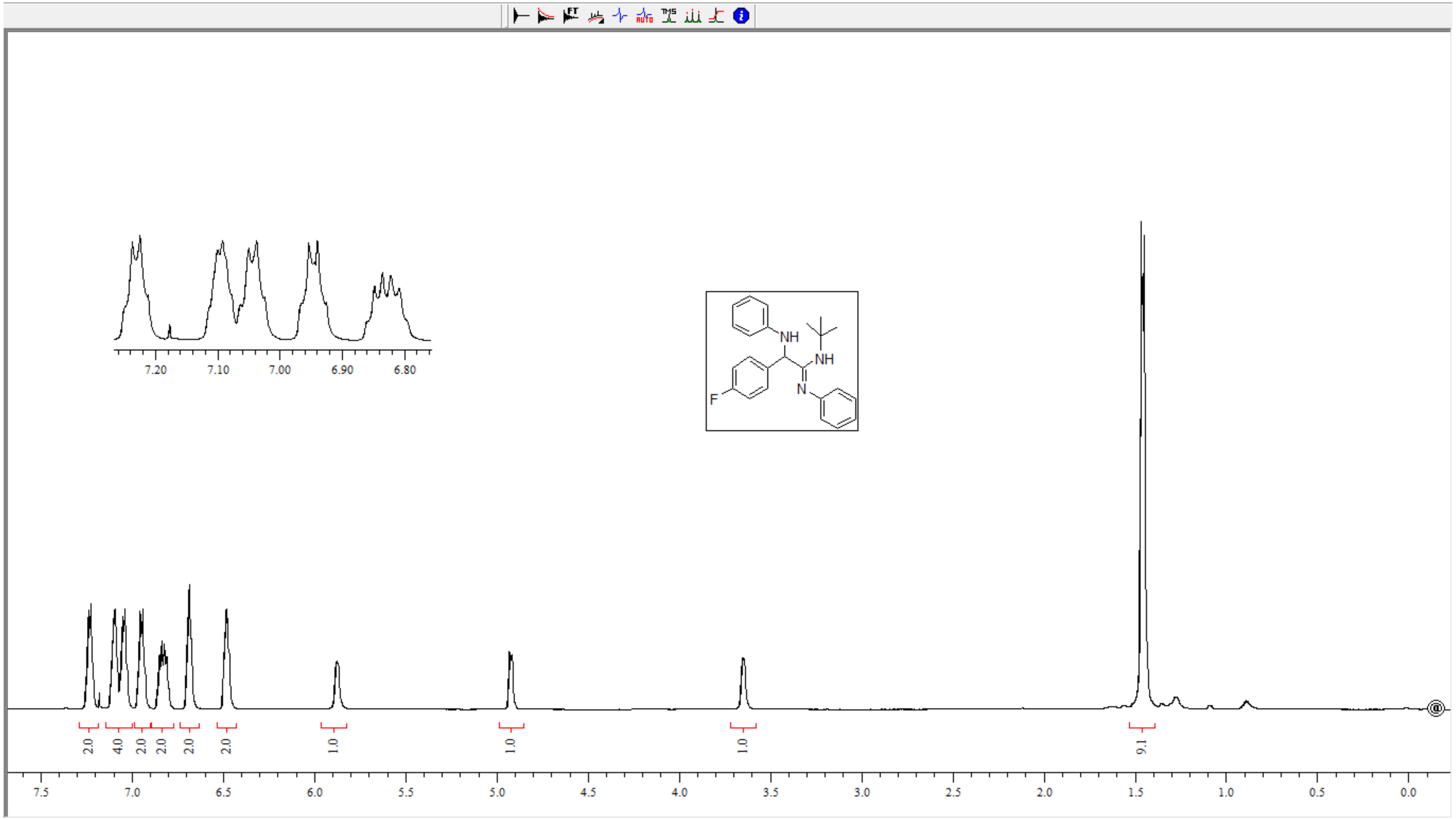

^{1}H NMR of compound **4j**

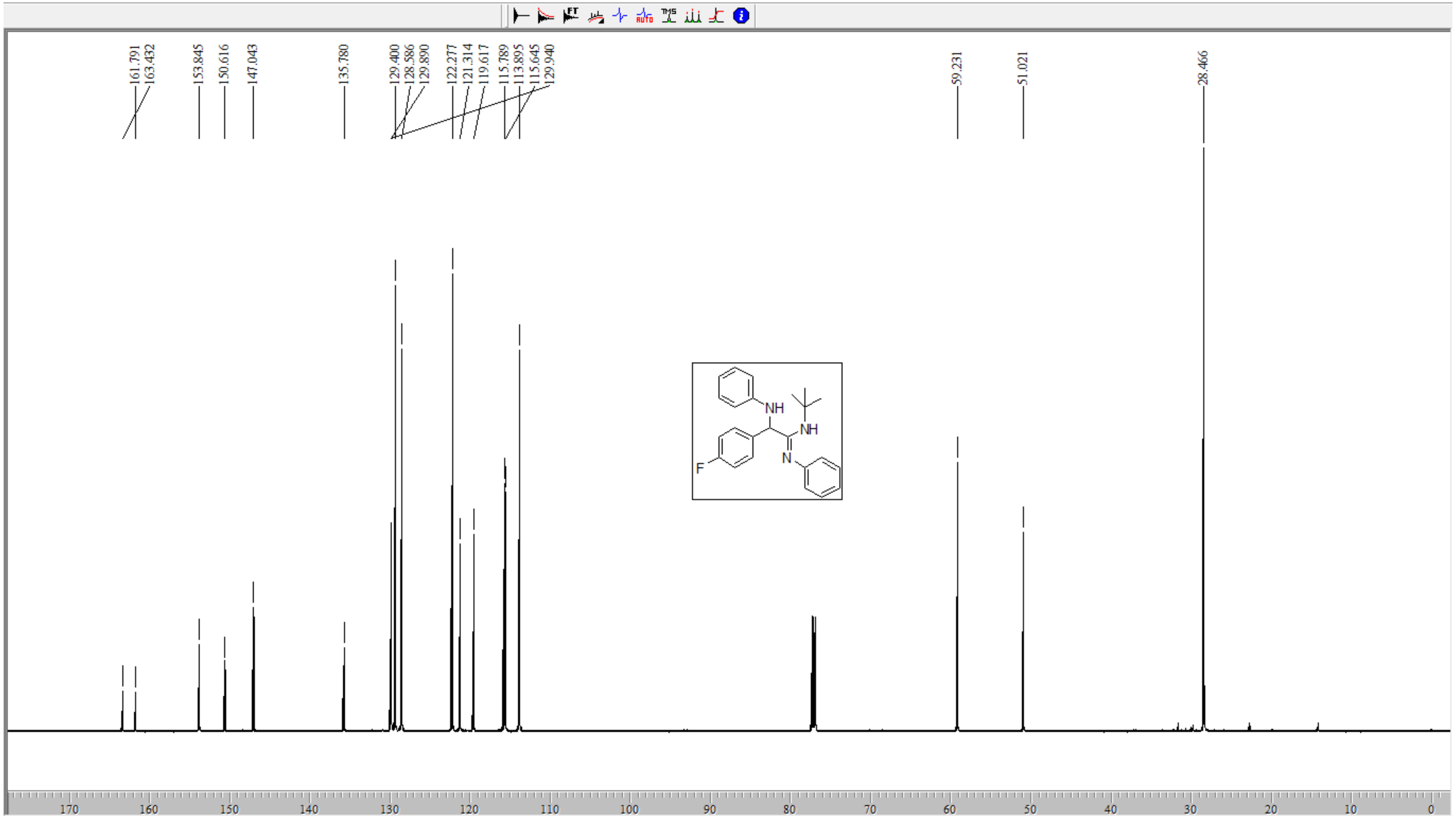

^{13}C NMR of compound **4j**

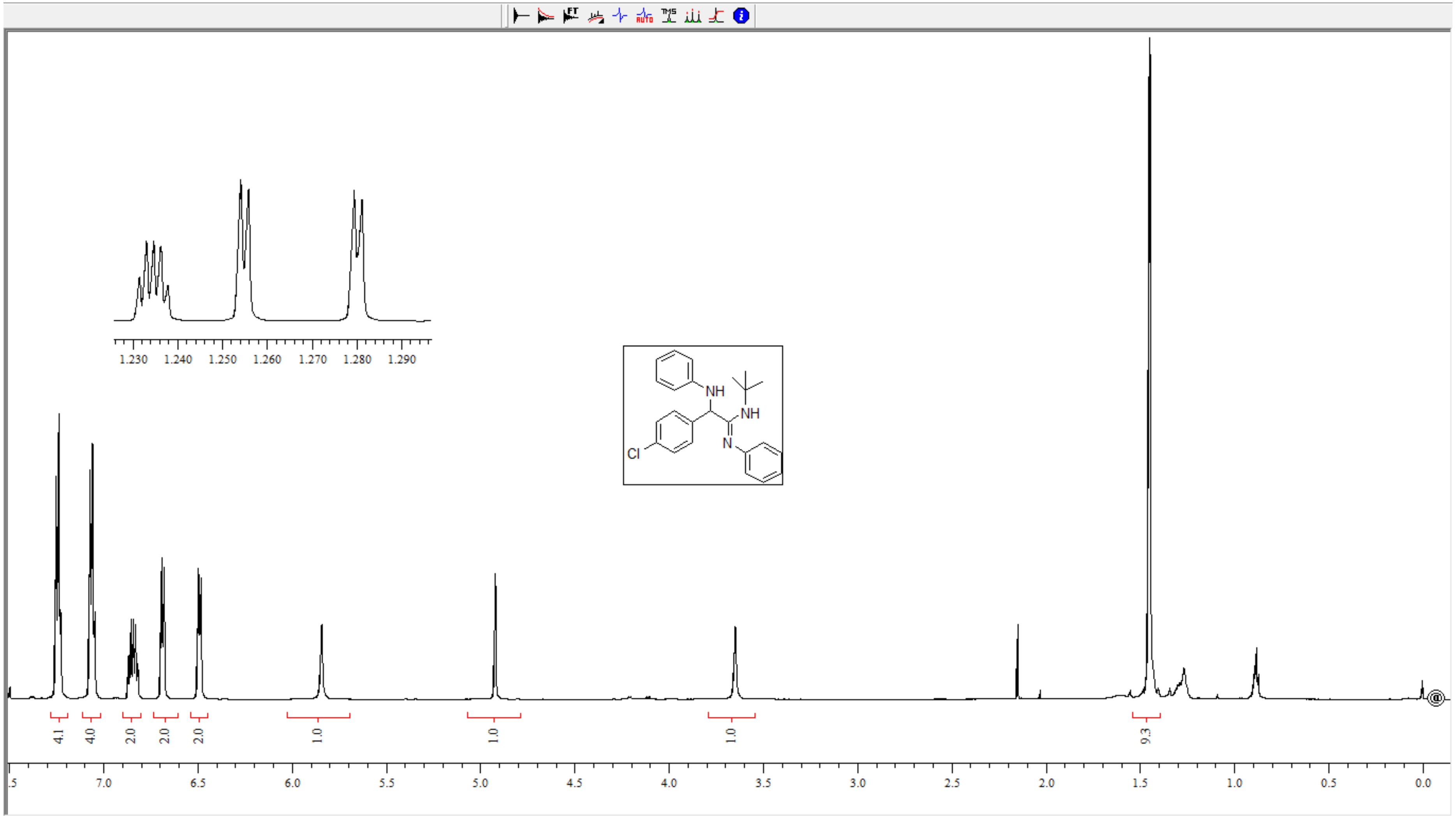

^{1}H NMR of compound **4k**

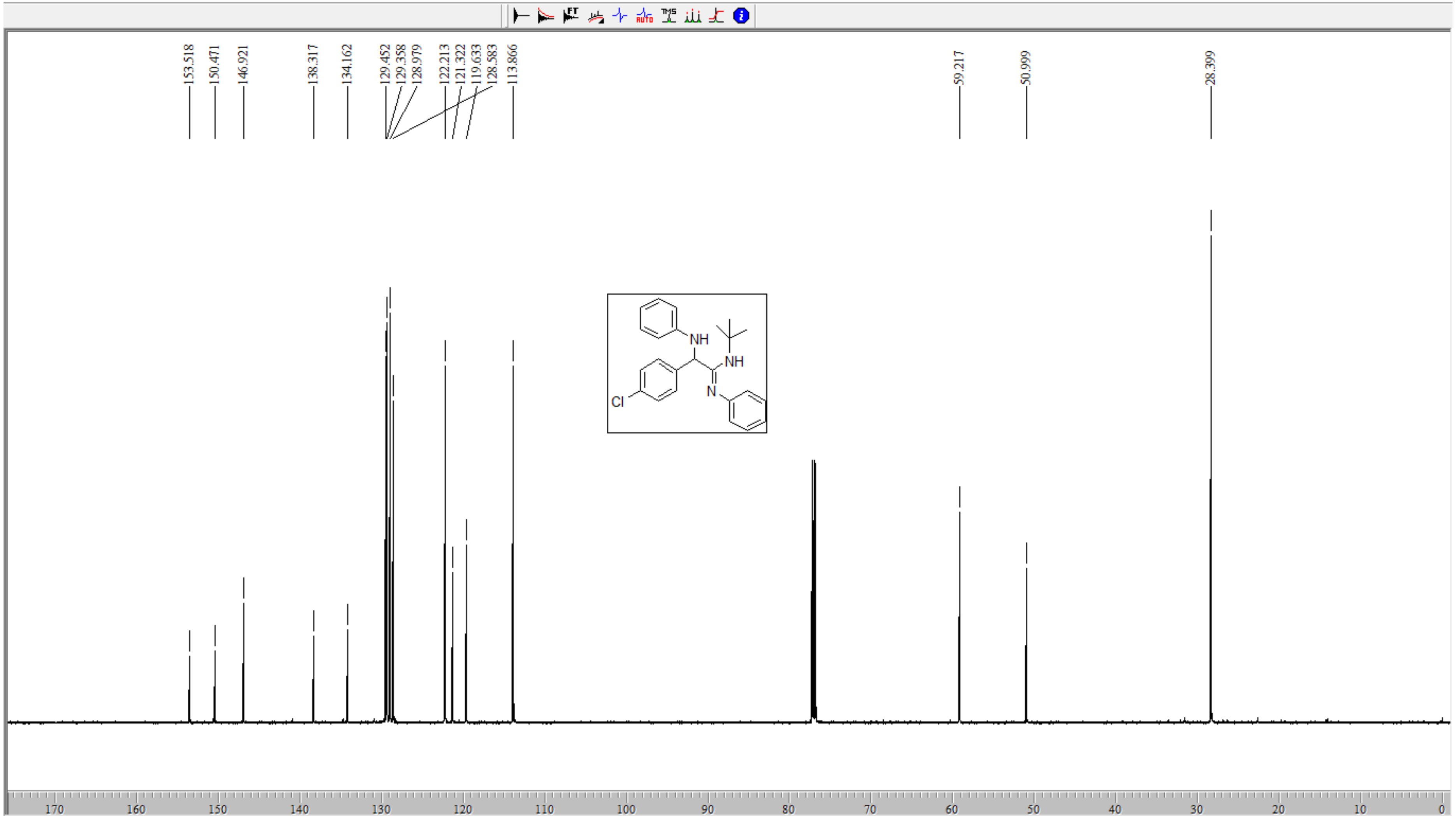

^{13}C NMR of compound **4k**

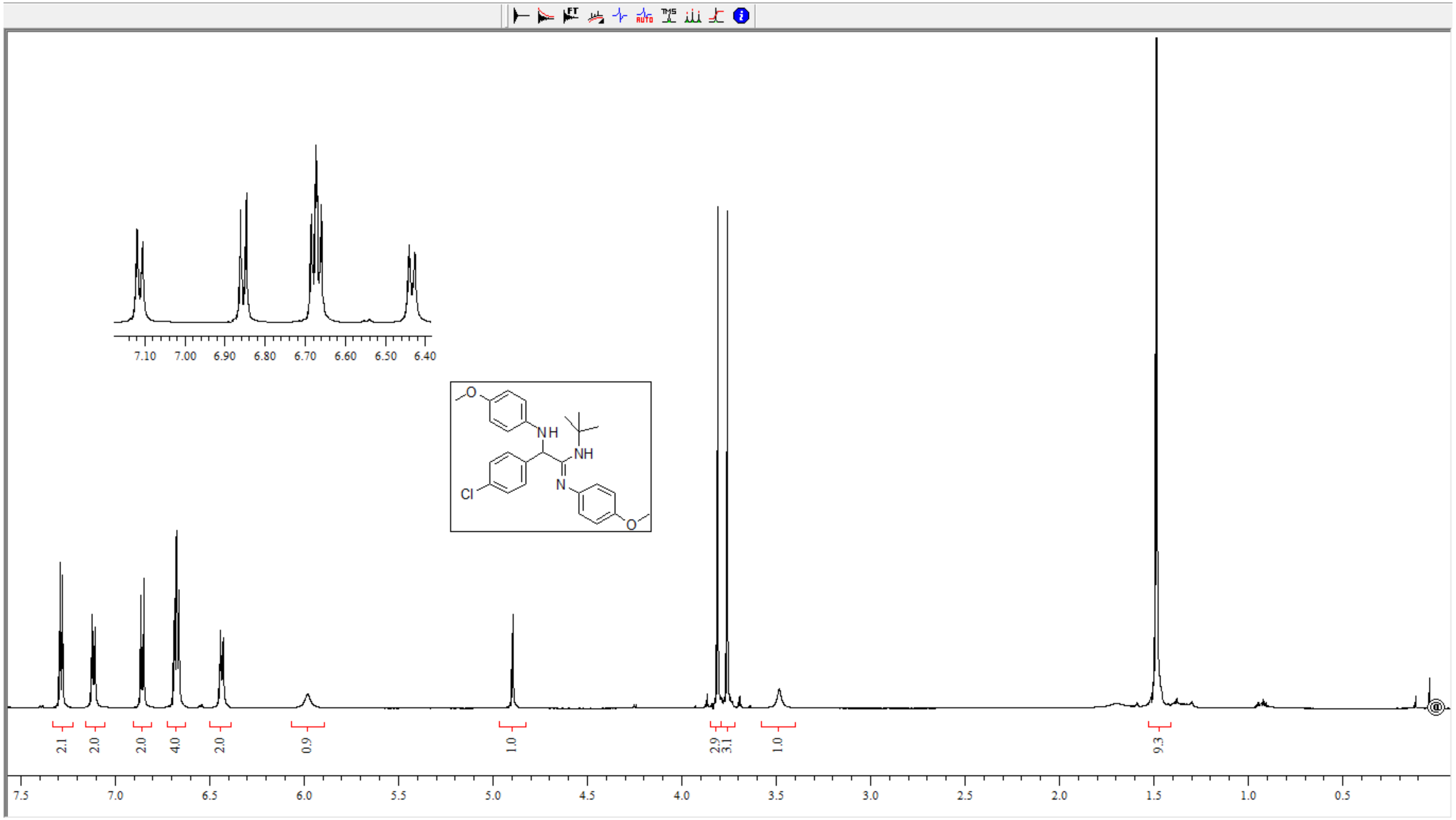

^{1}H NMR of compound **4l**

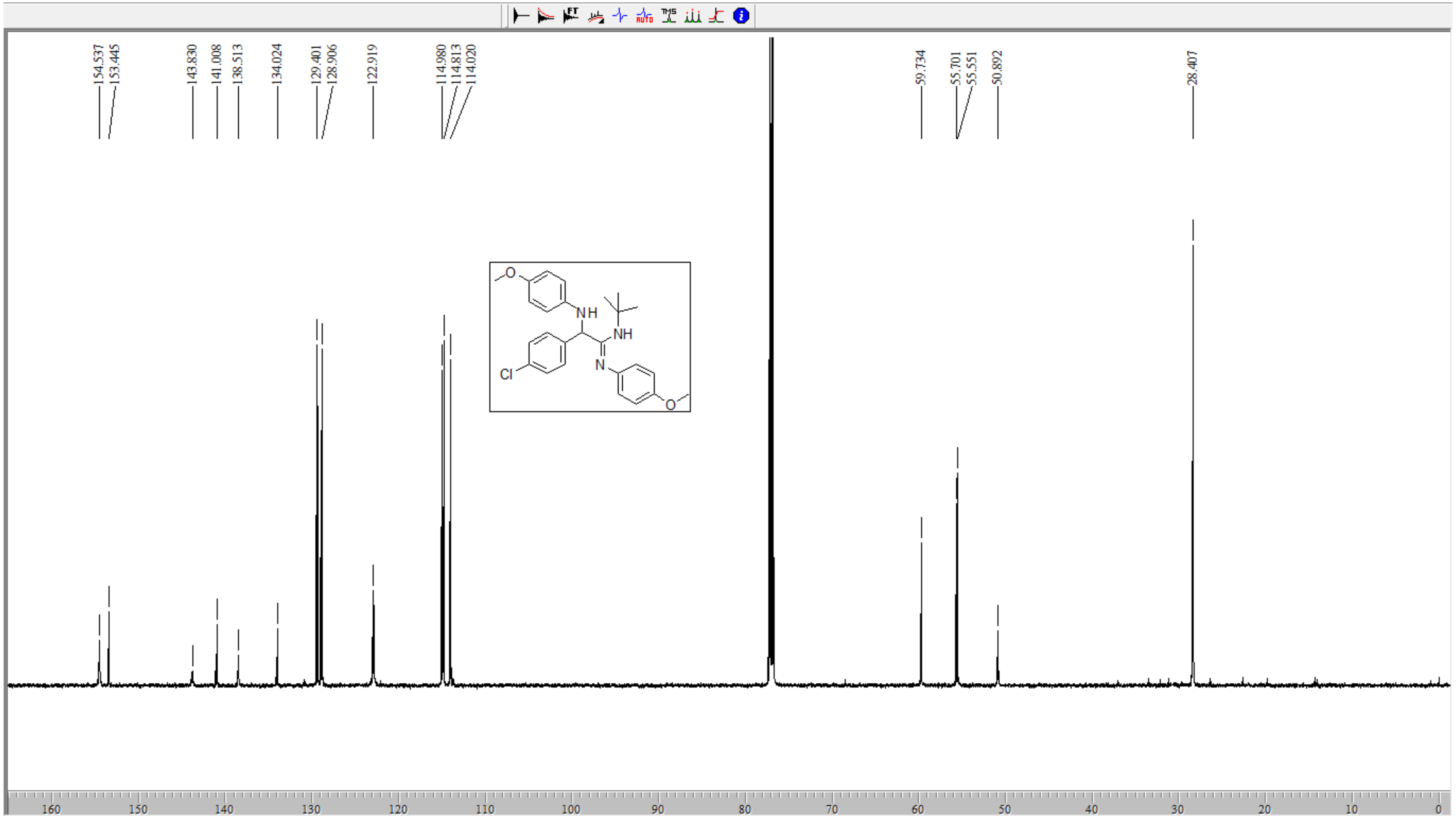

^{13}C NMR of compound **4l**

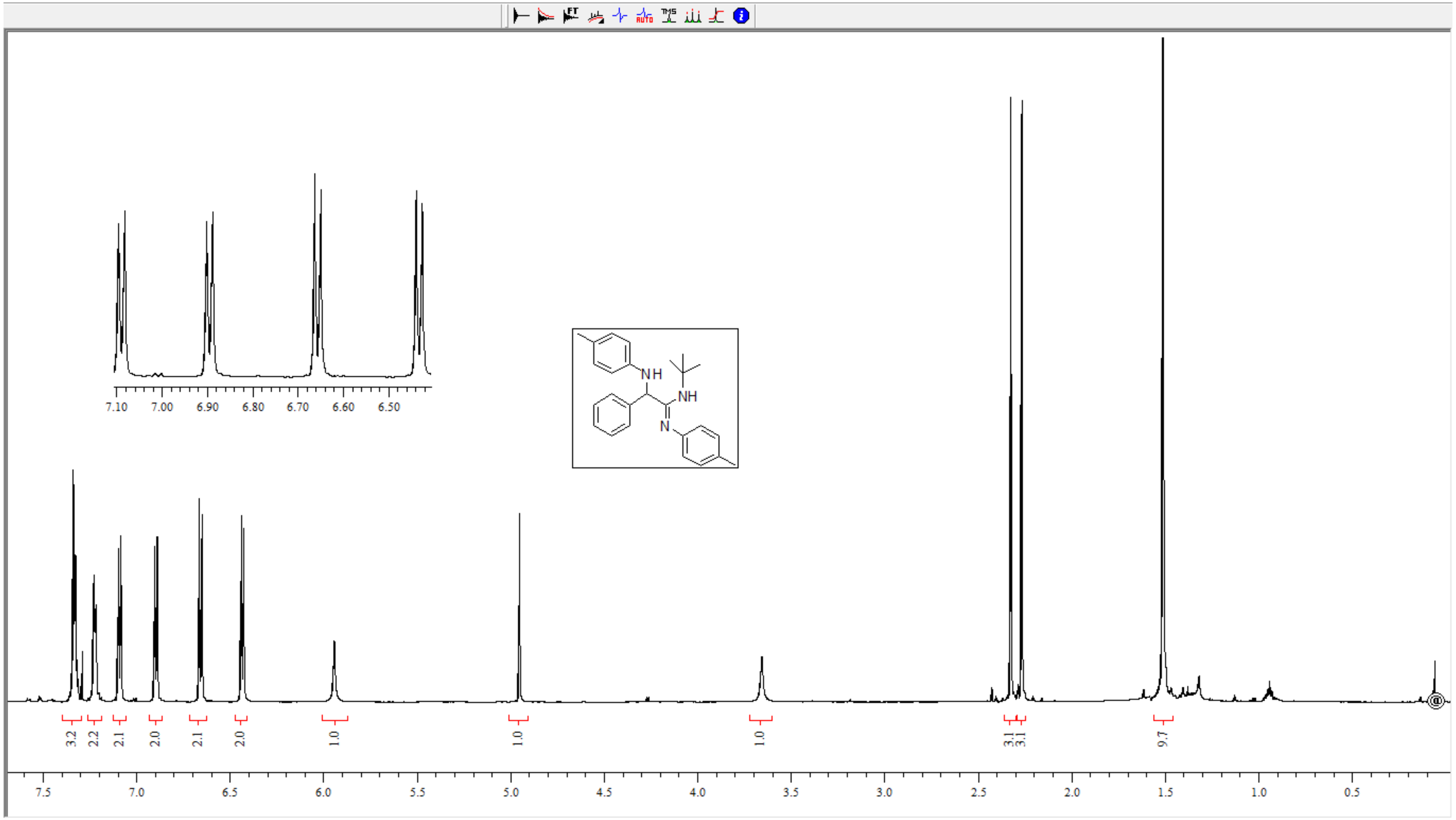

^{1}H NMR of compound **4m**

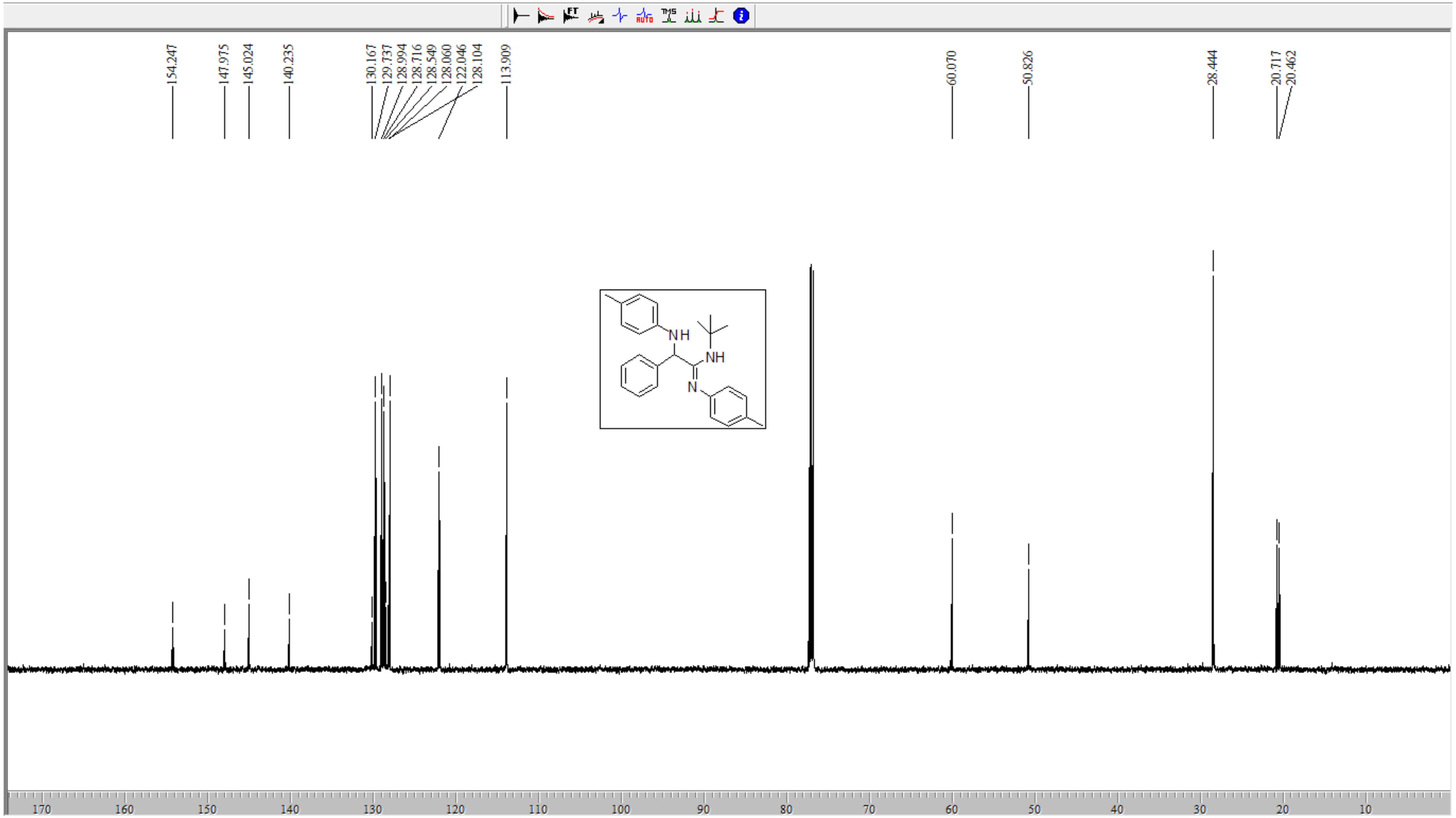

^{13}C NMR of compound **4m**

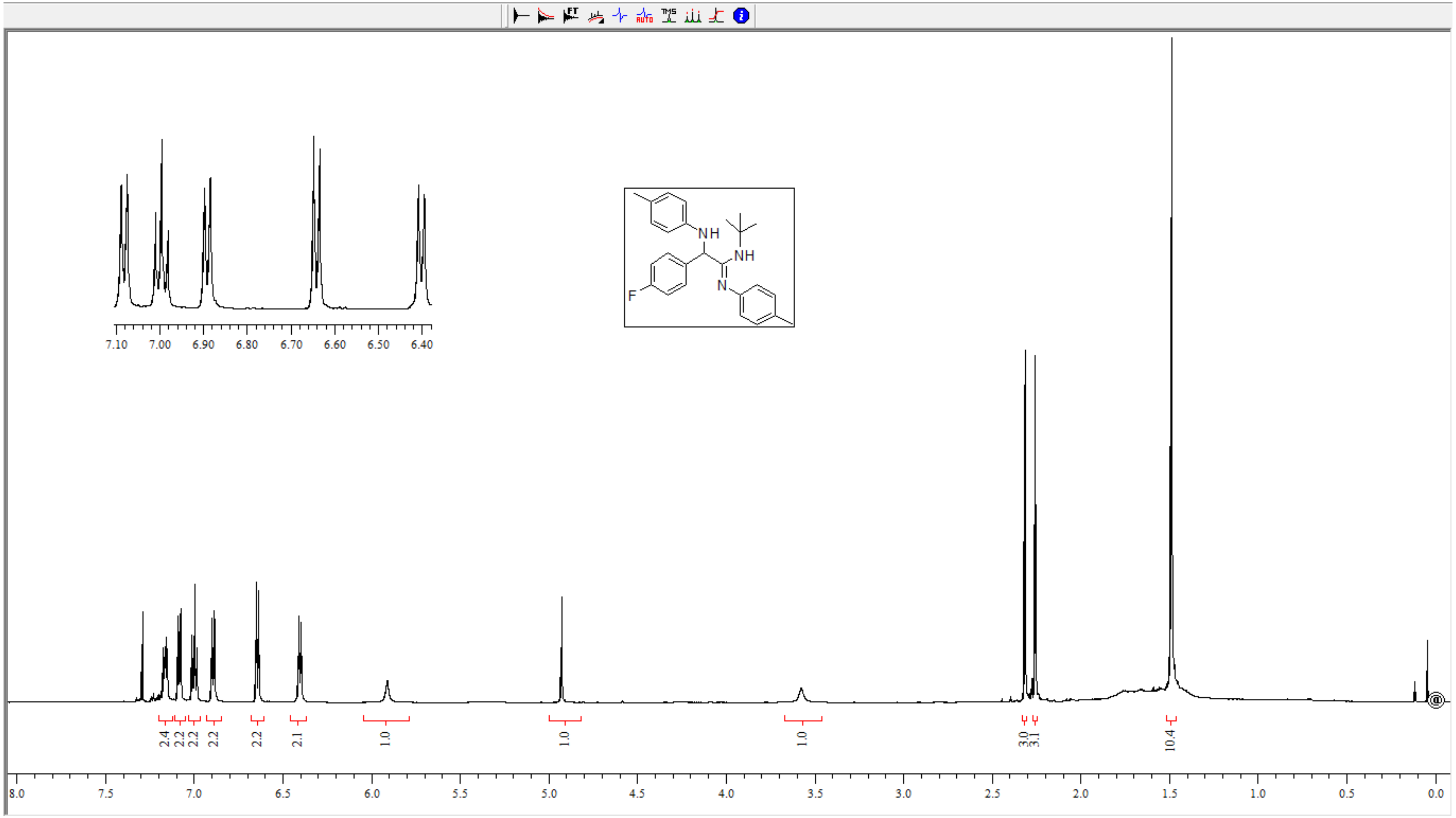

^{1}H NMR of compound **4n**

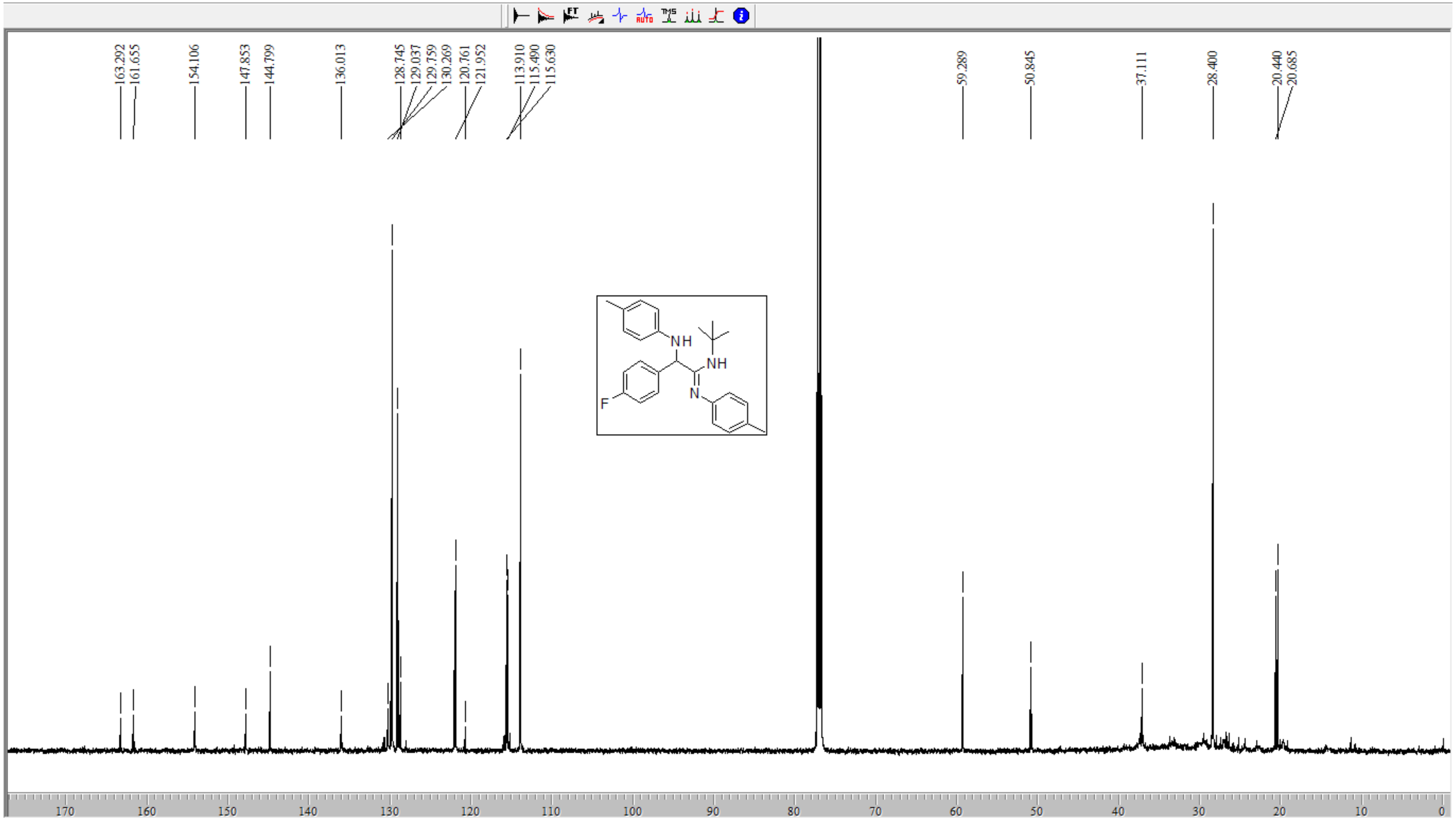

^{13}C NMR of compound **4n**

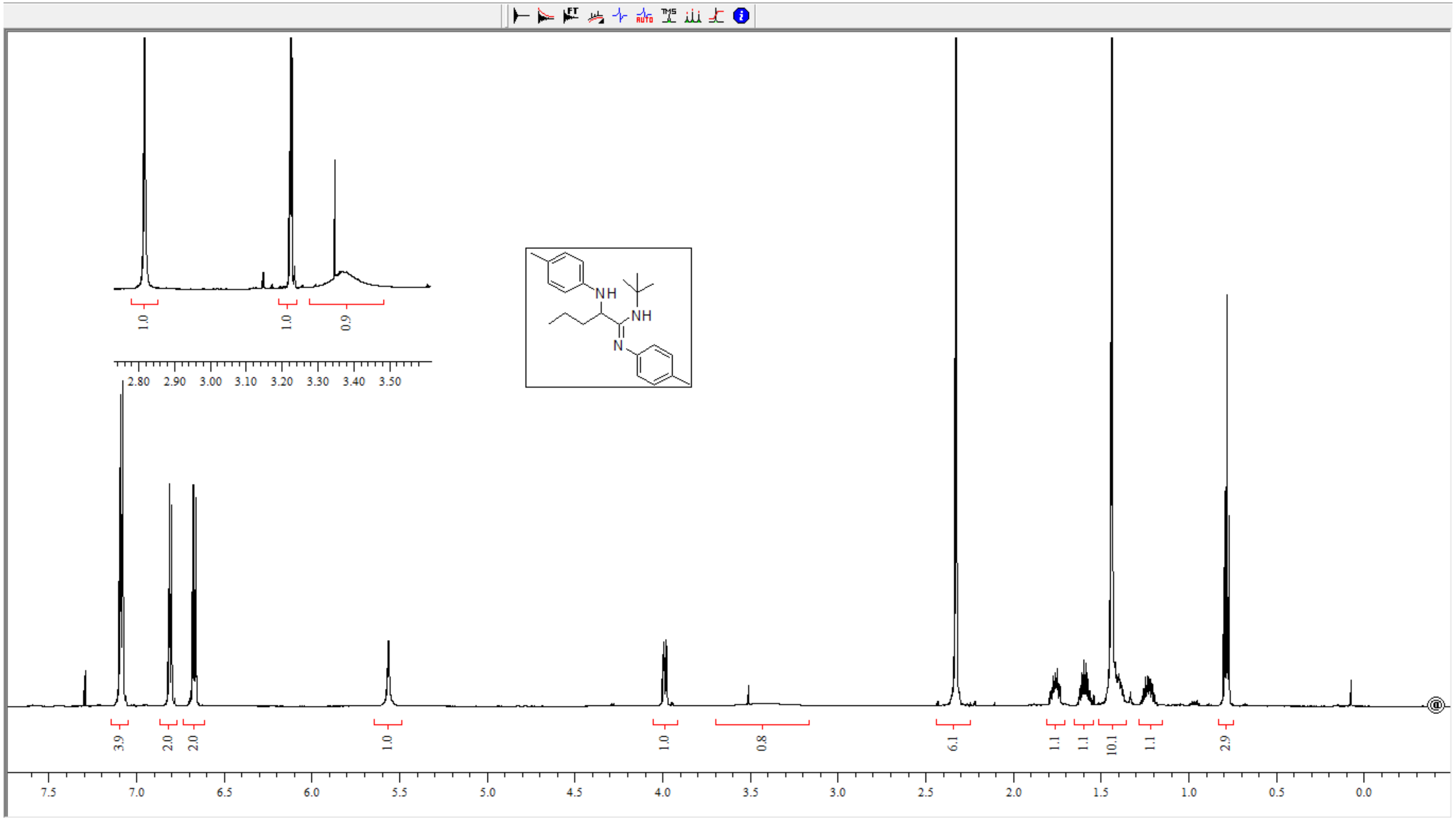

^{1}H NMR of compound **4o**

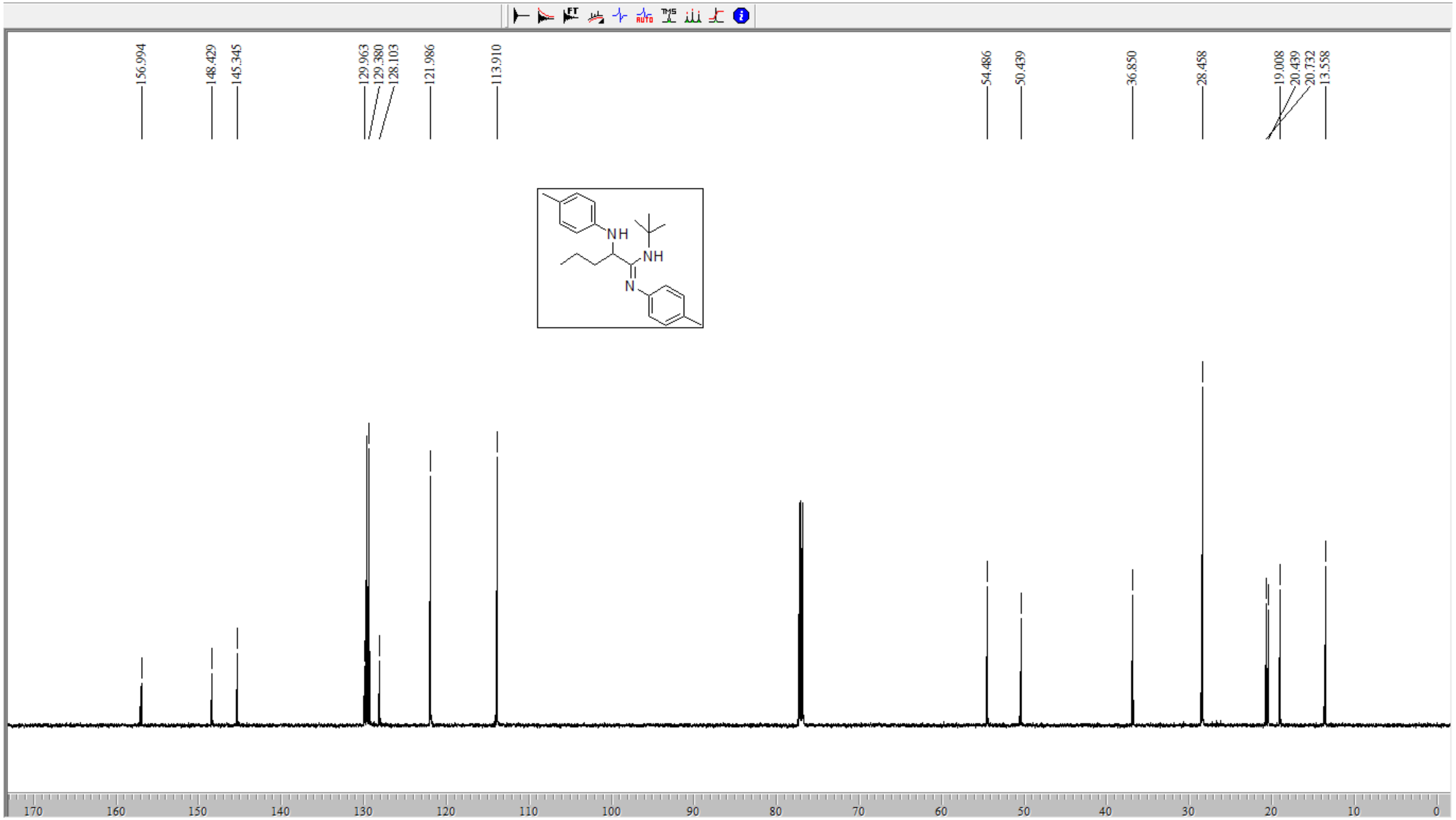

^{13}C NMR of compound **4o**

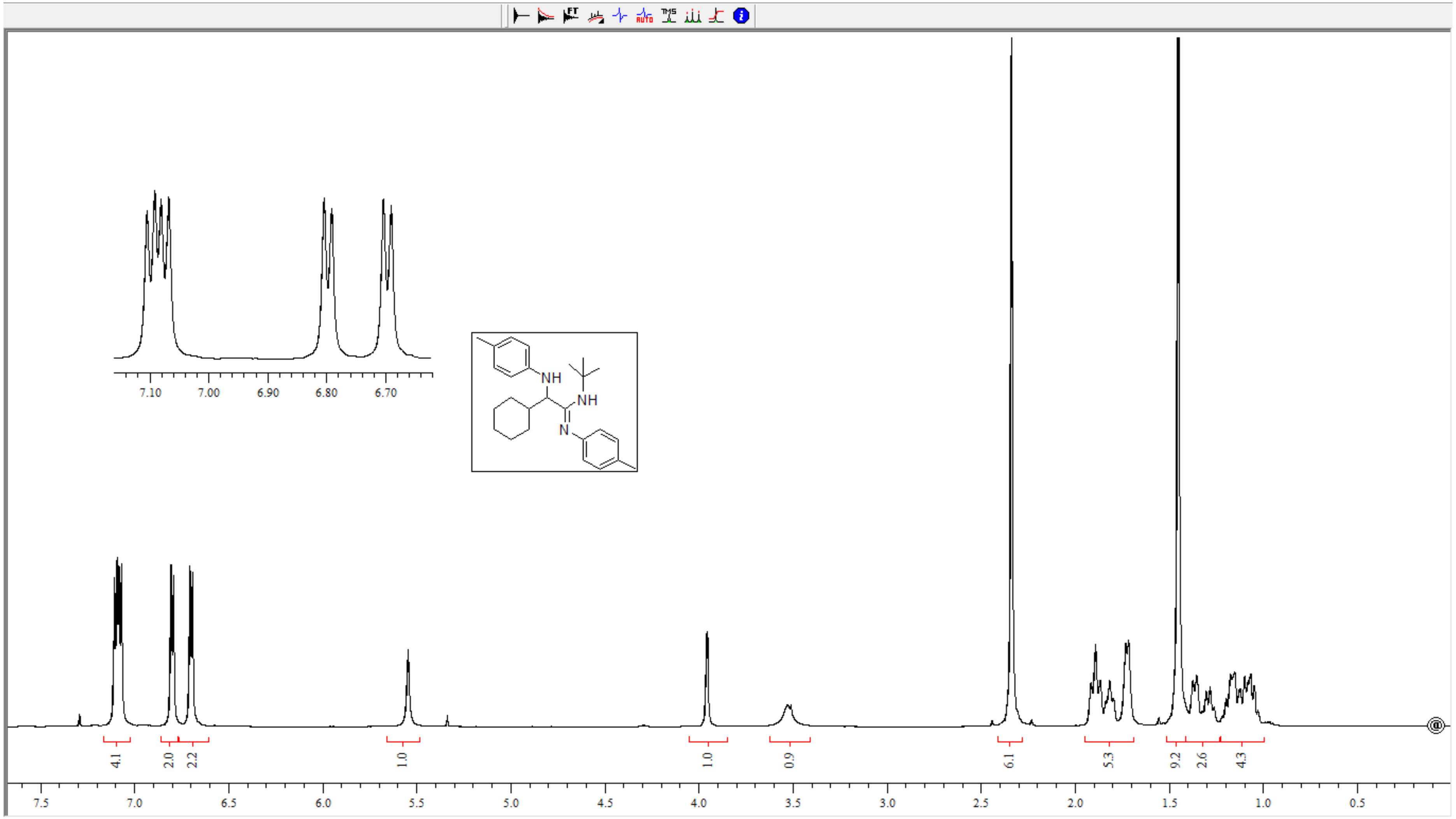

^{1}H NMR of compound **4p**

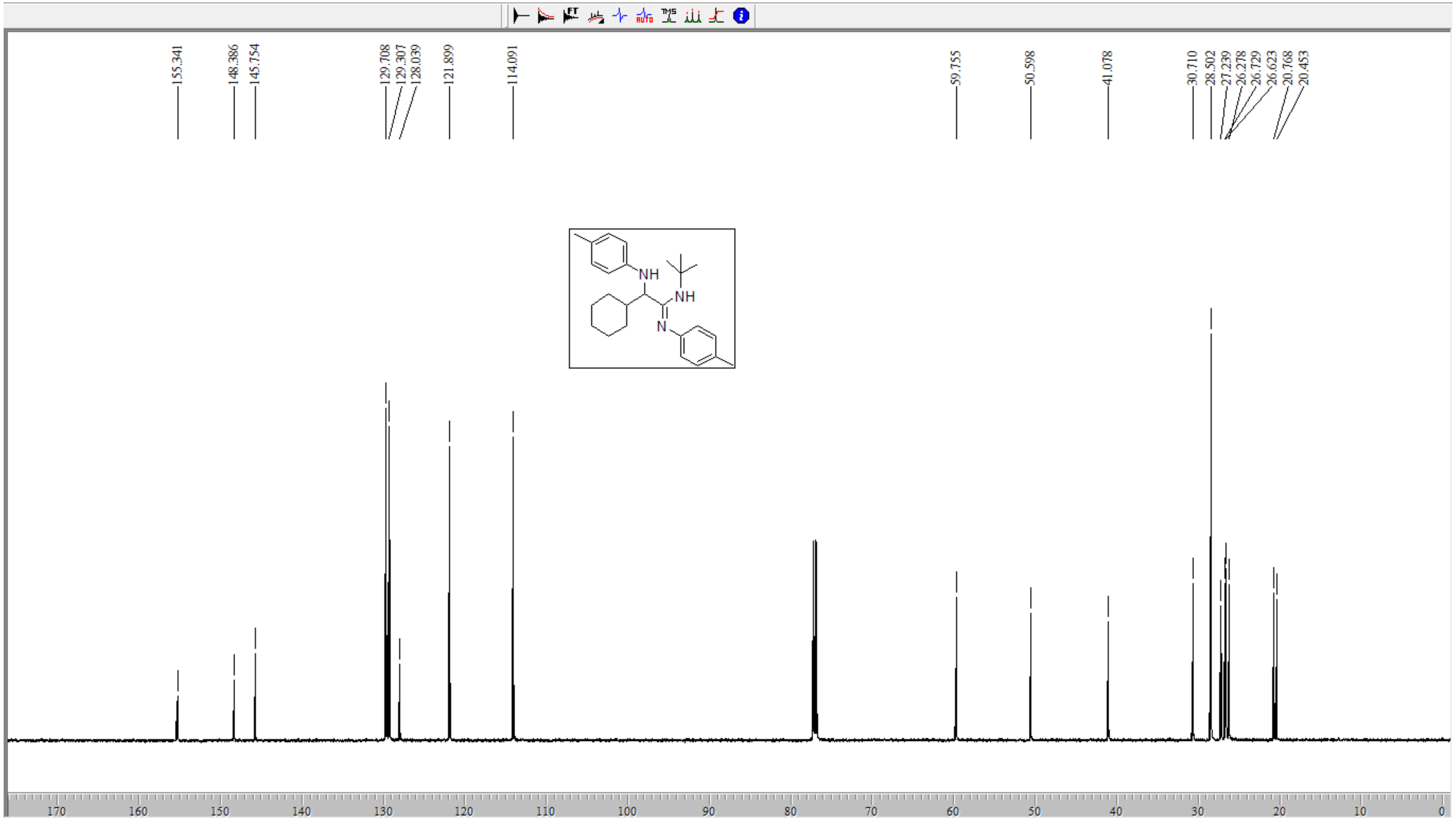

^{13}C NMR of compound **4p**

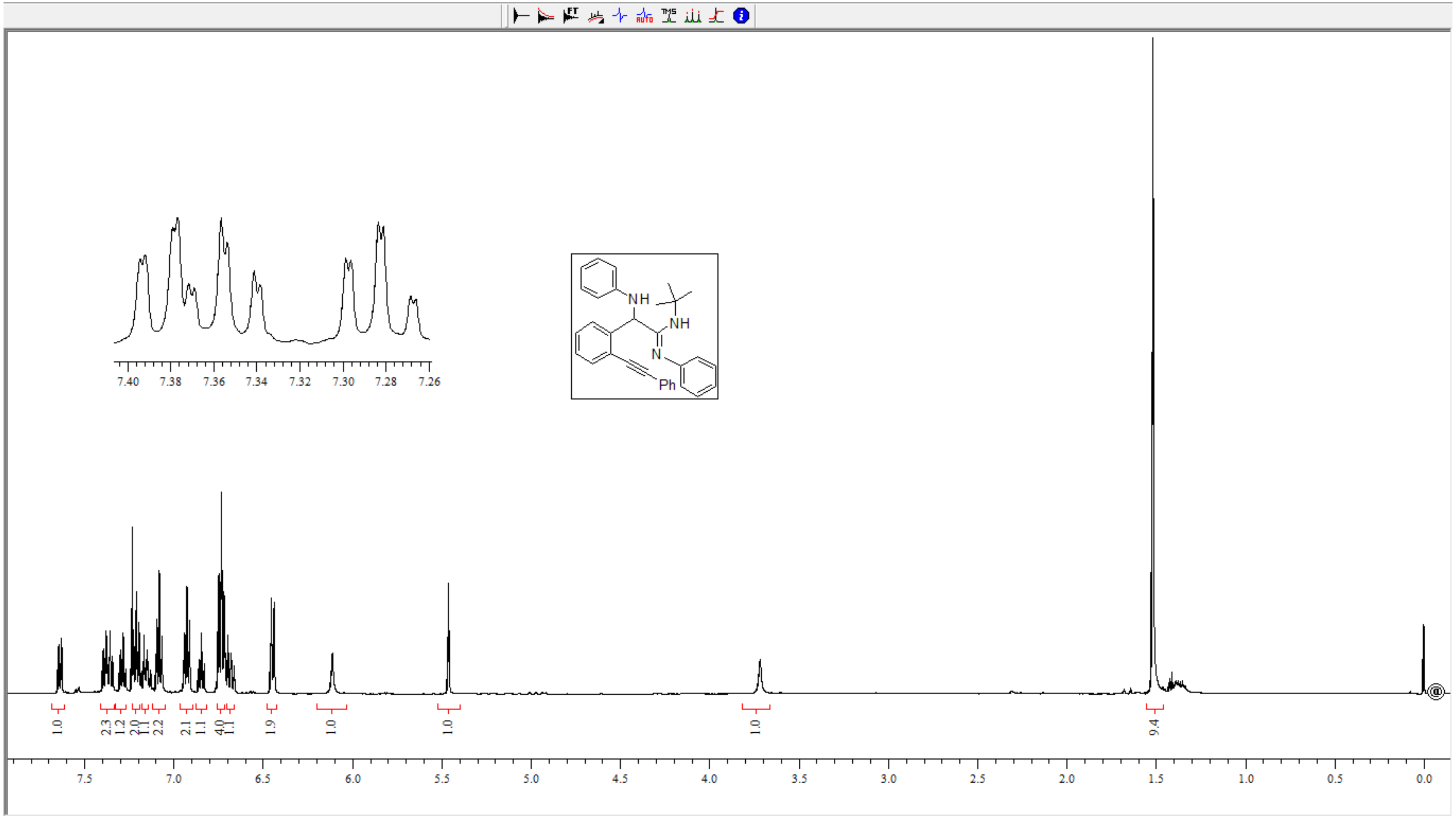

^{1}H NMR of compound **4q**

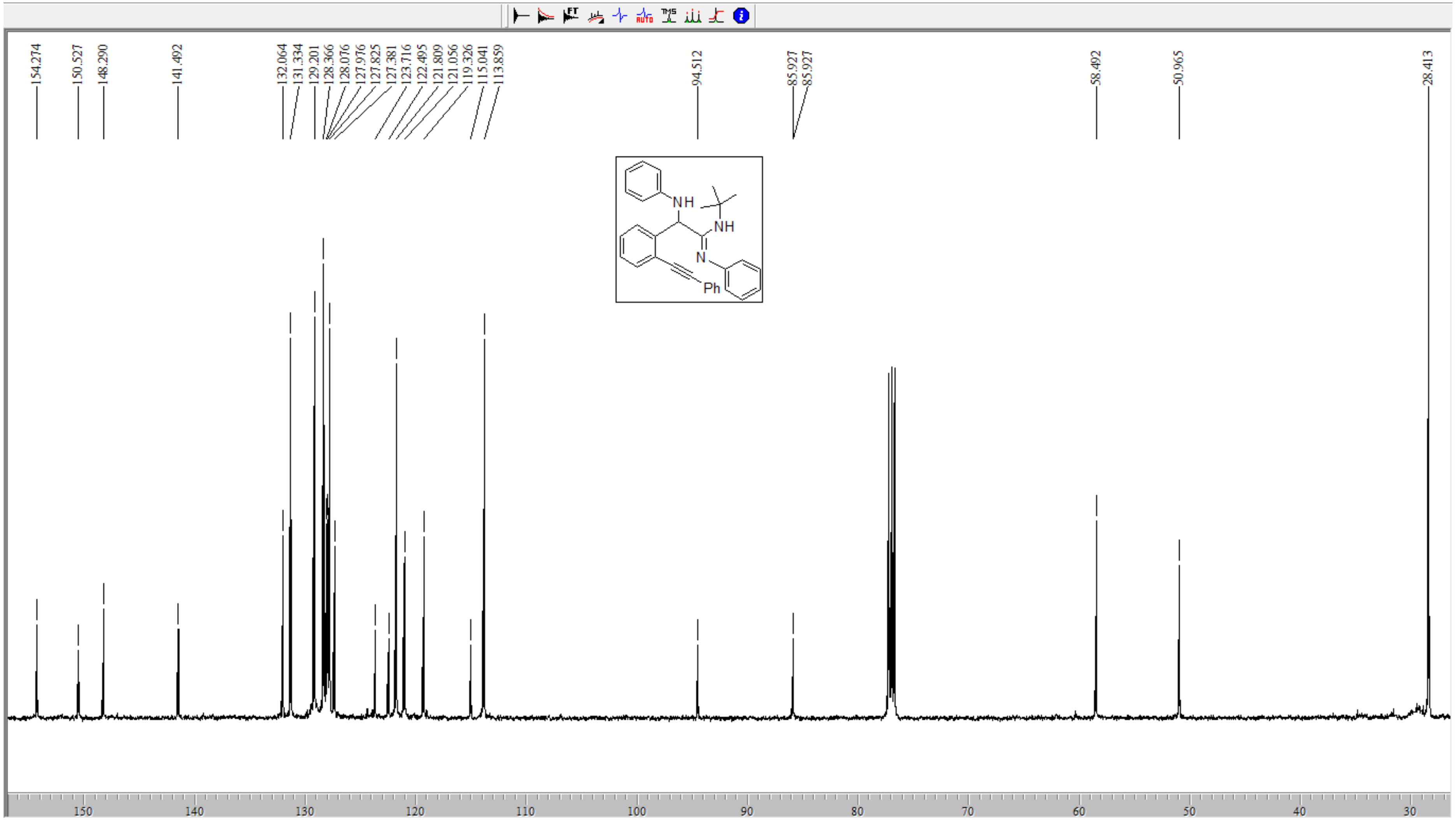

^{13}C NMR of compound **4q**

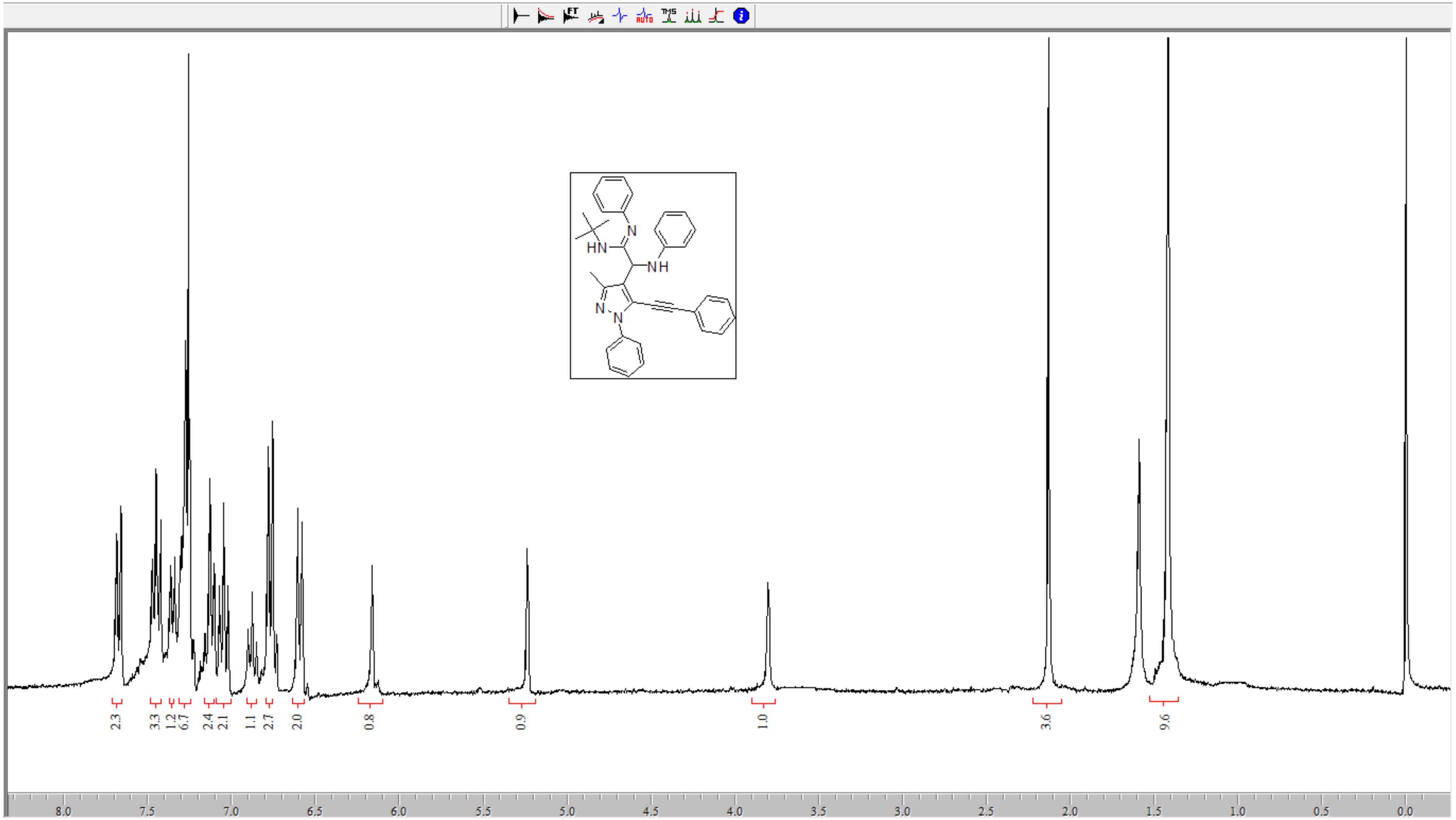

^{1}H NMR of compound **4r**

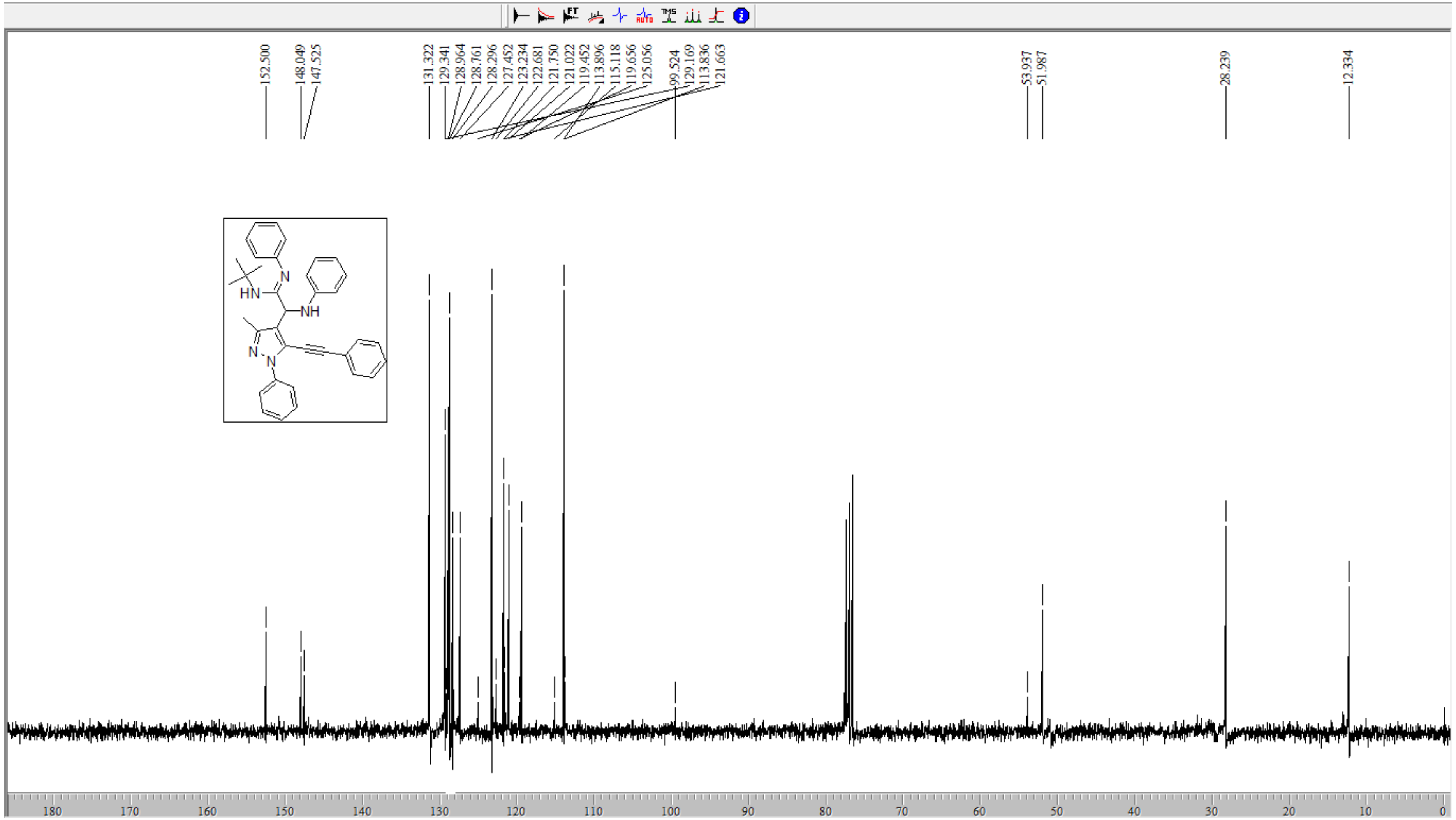

^{13}C NMR of compound **4r**

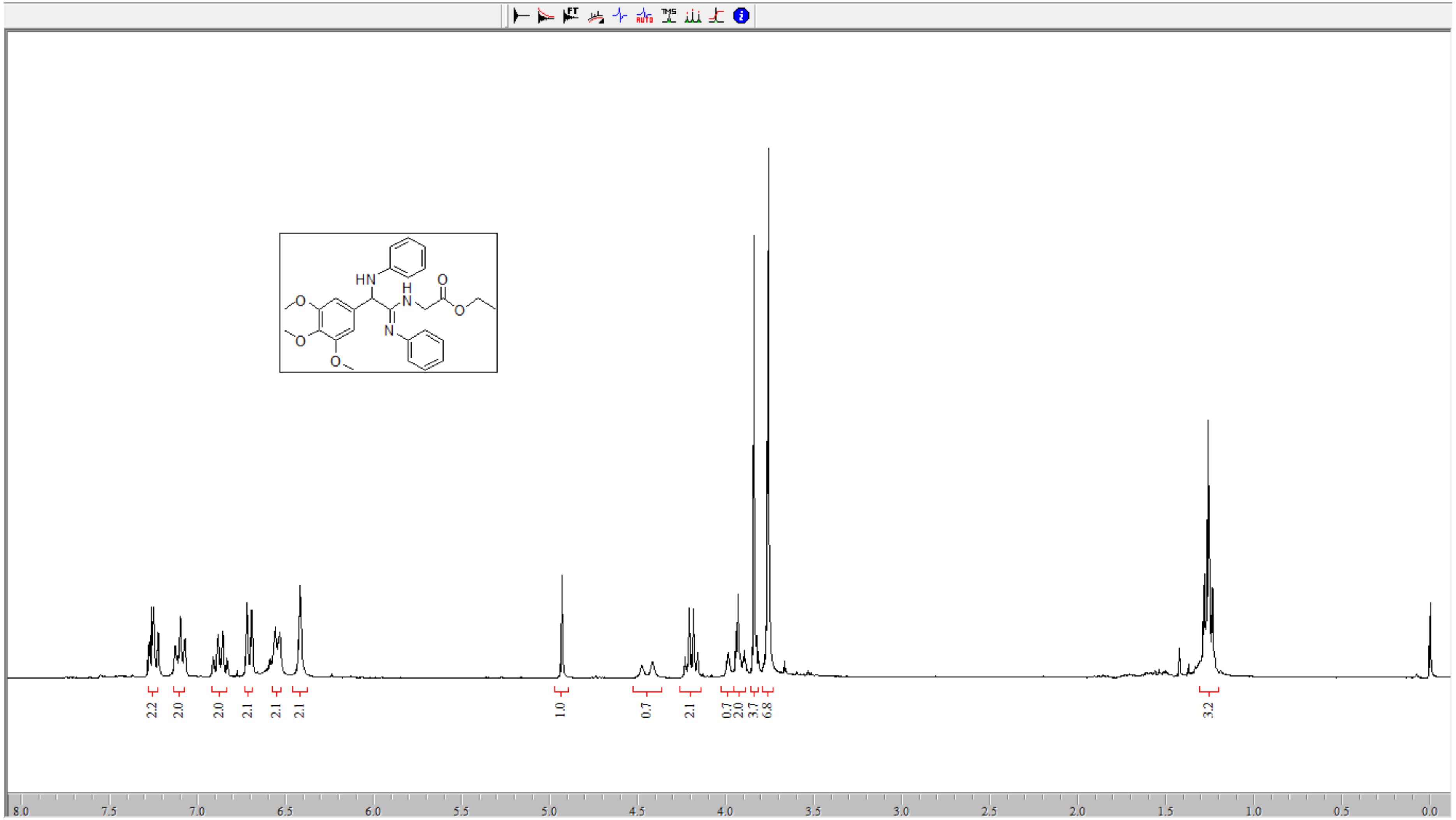

^{1}H NMR of compound **4s**

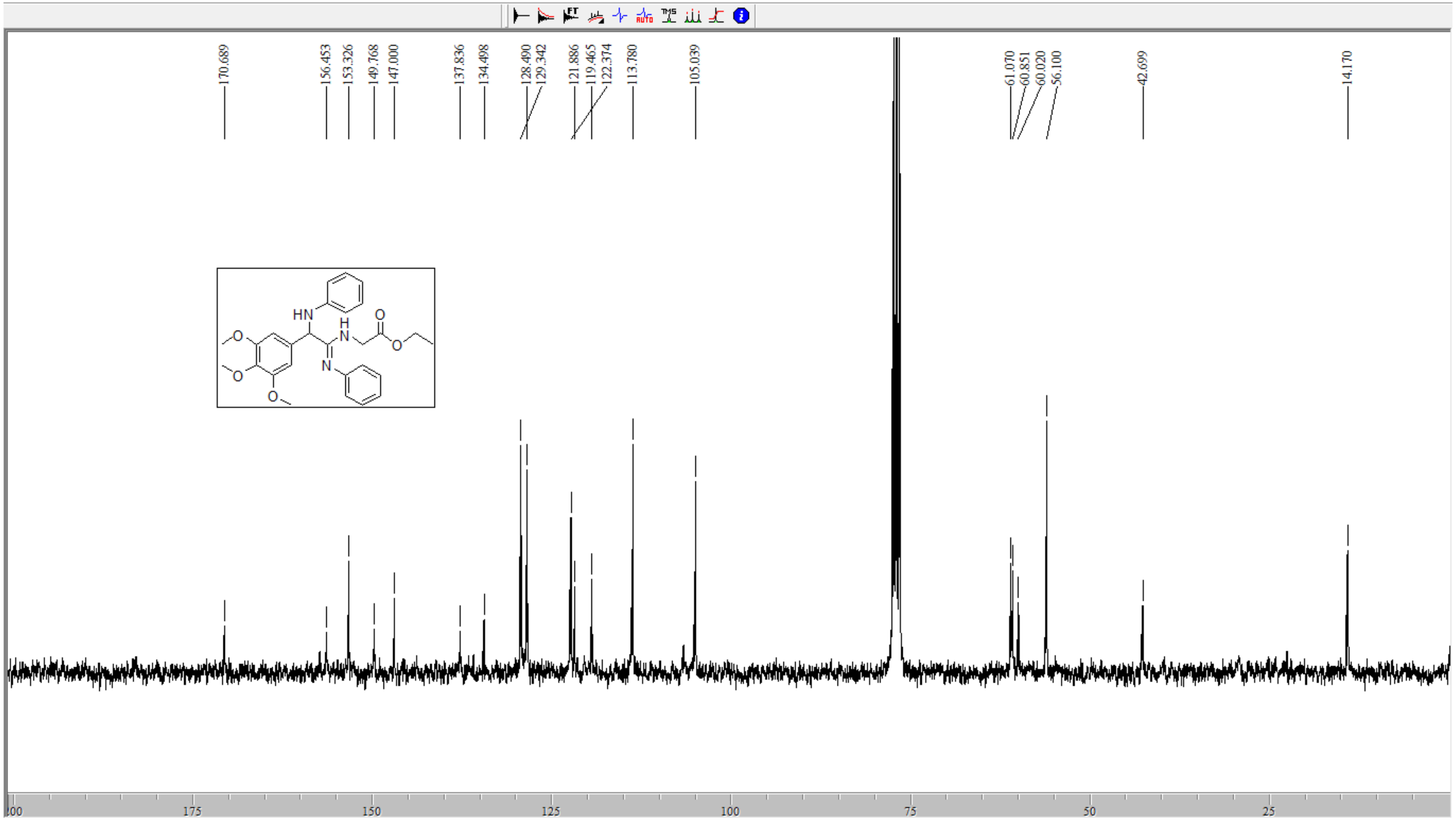

^{13}C NMR of compound **4s**

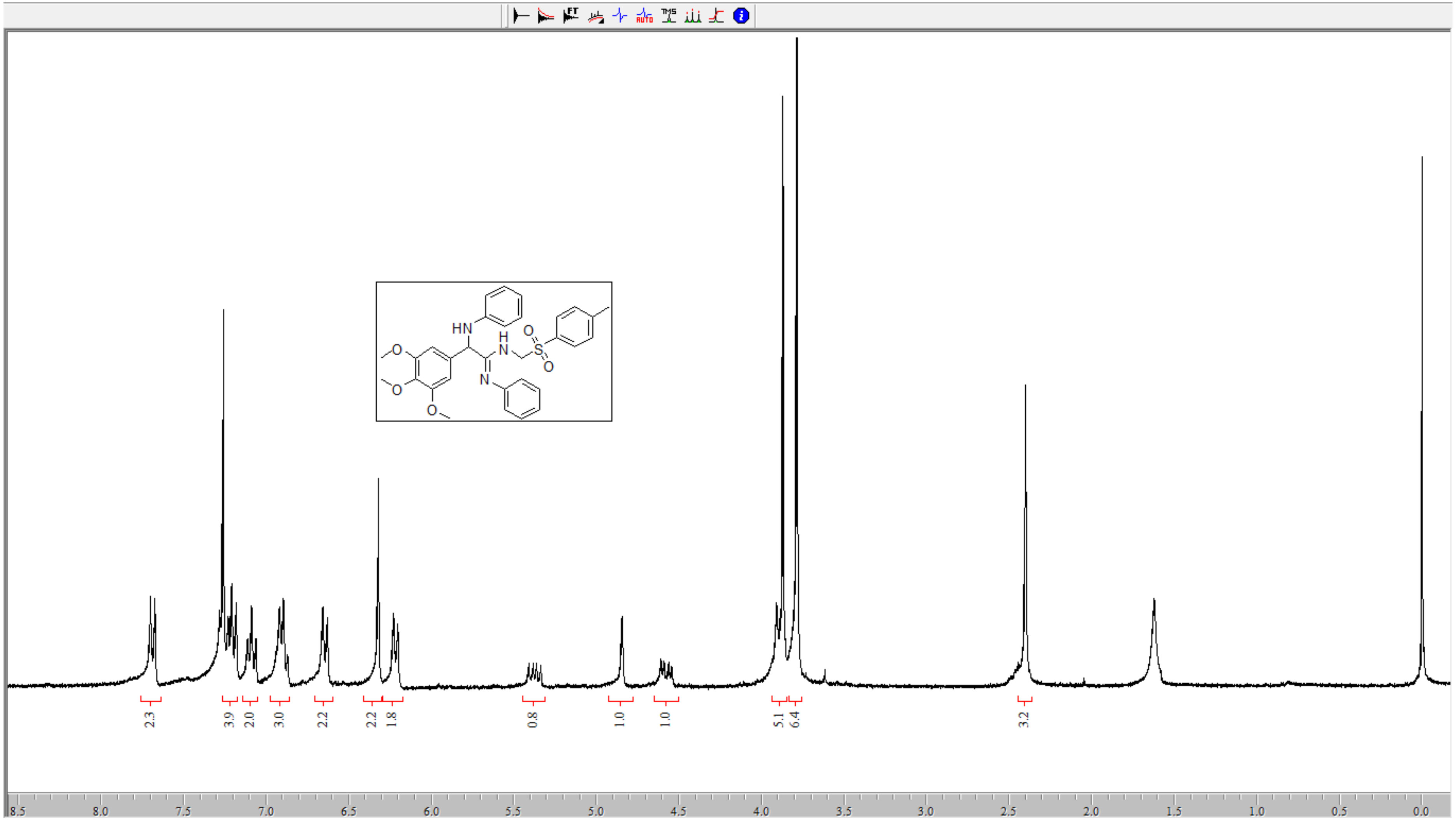

^{1}H NMR of compound **4t**

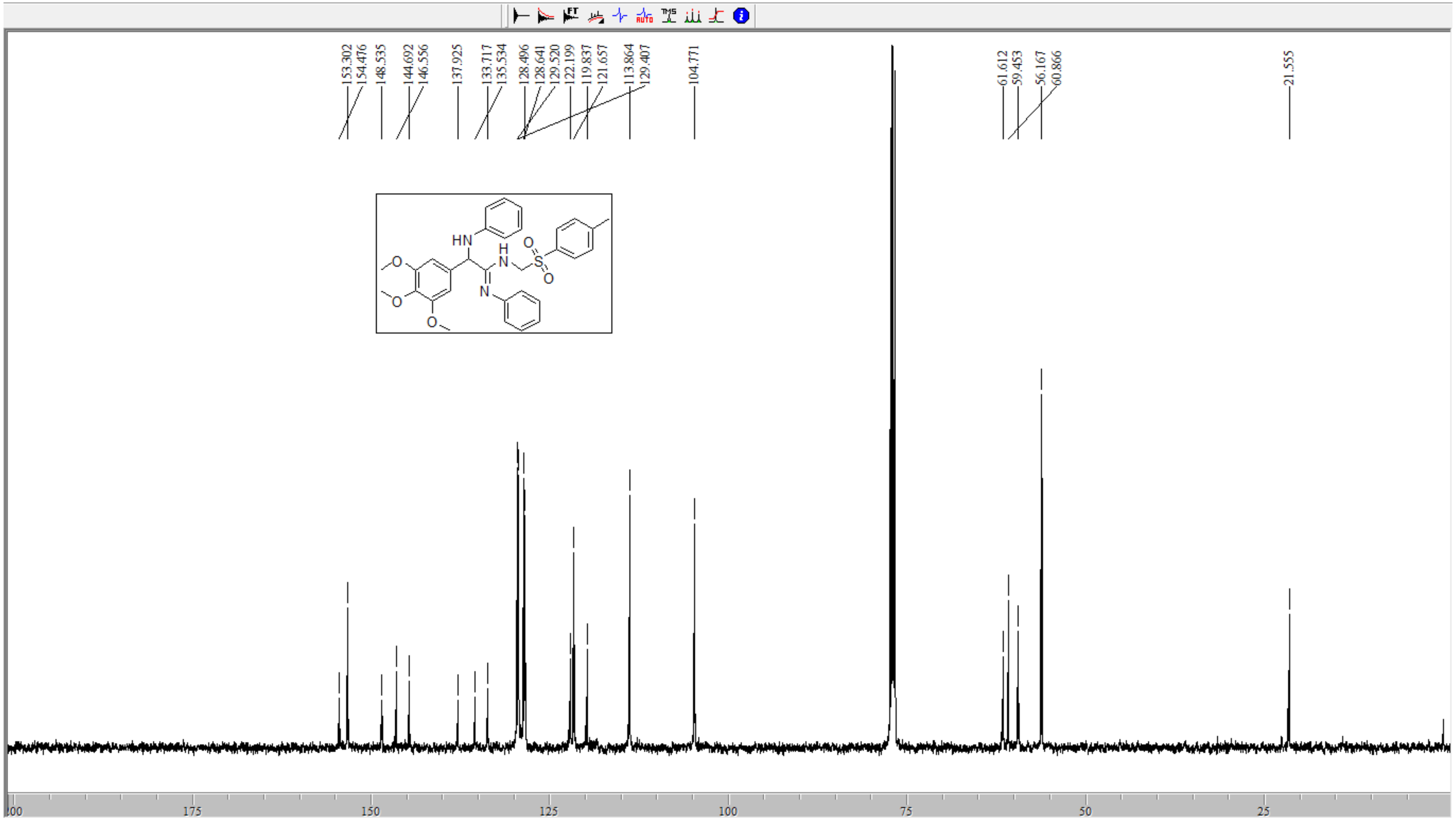

^{13}C NMR of compound **4t**

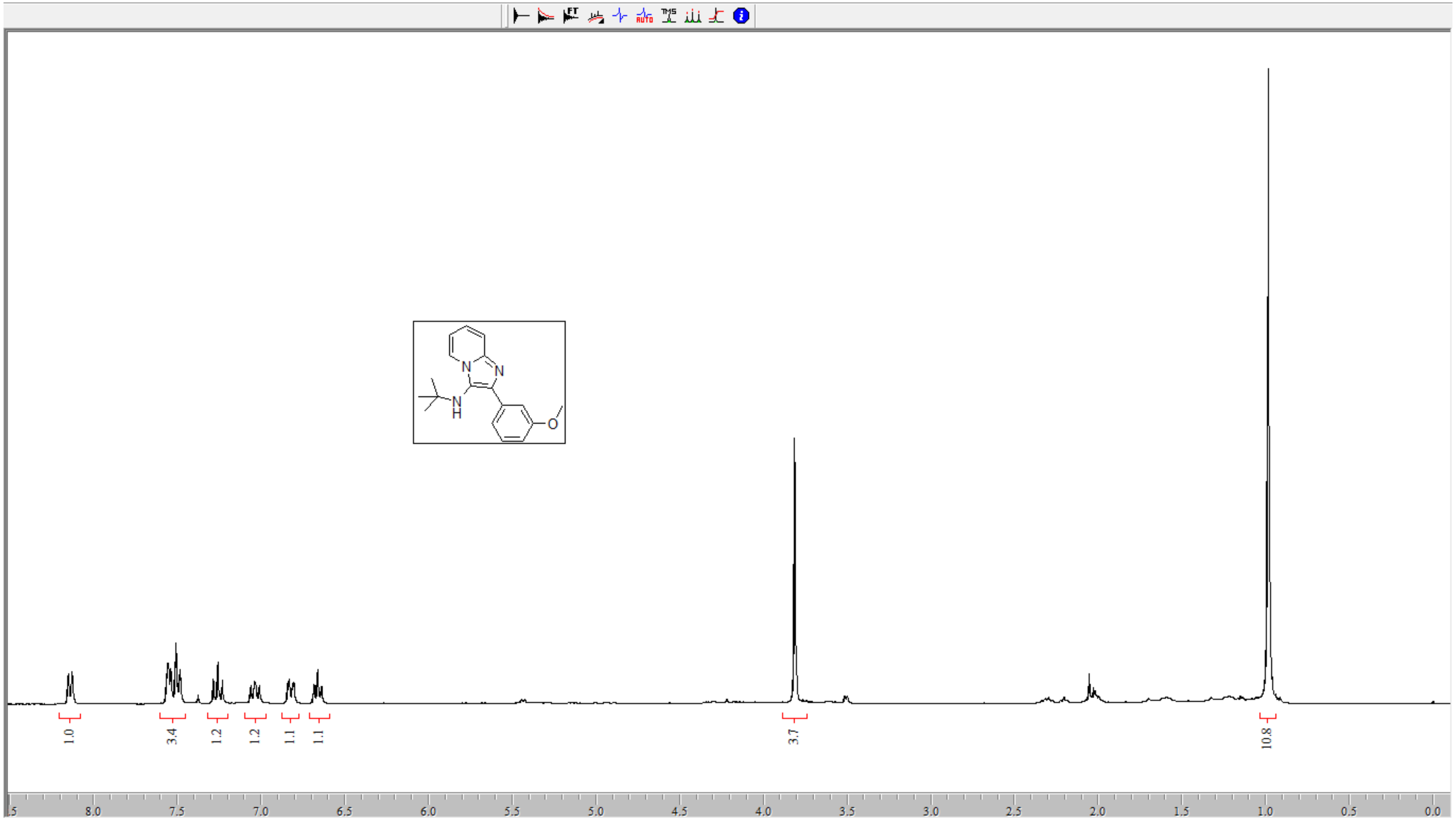

^{1}H NMR of compound **7a**

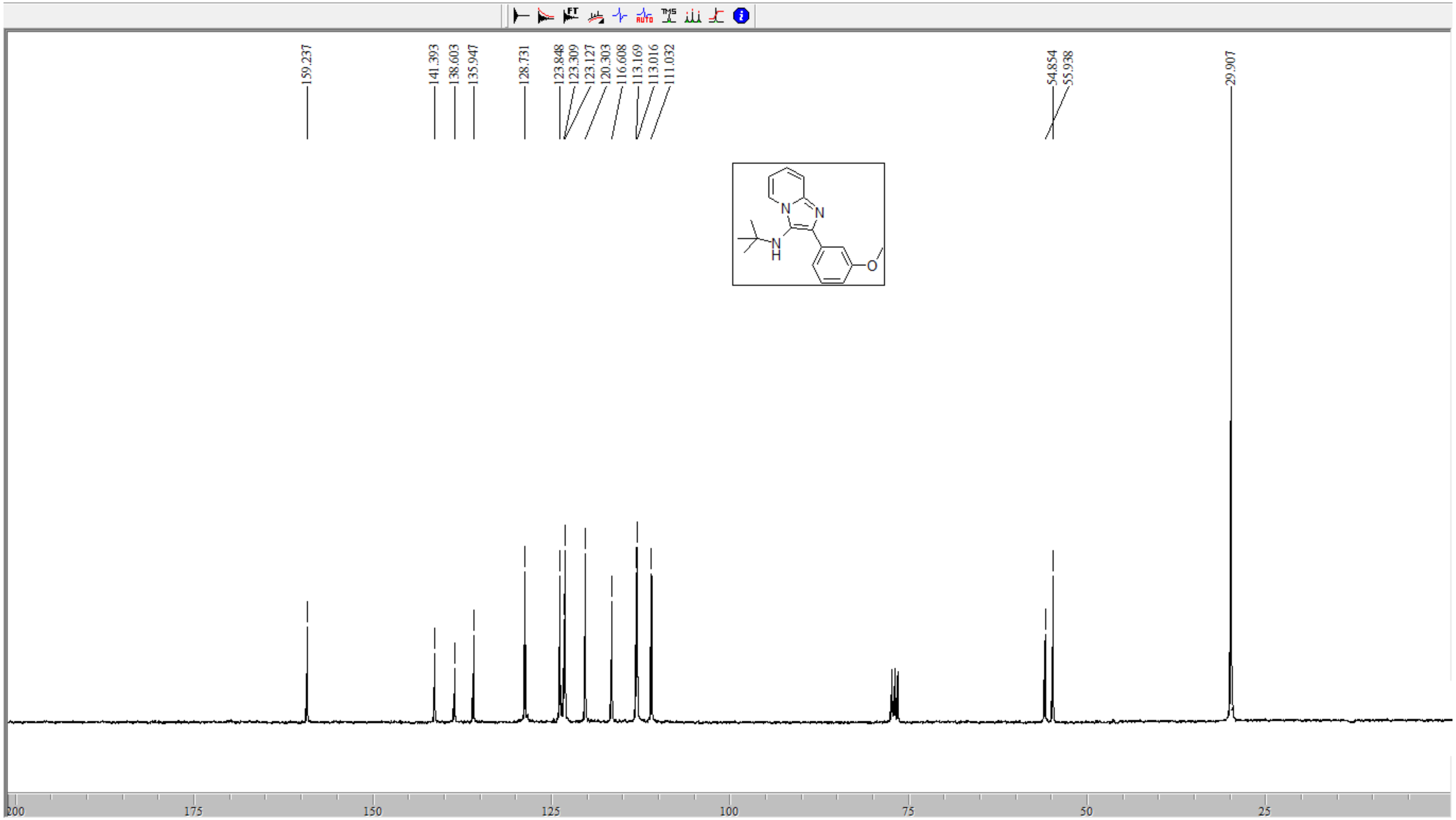

^{13}C NMR of compound **7a**

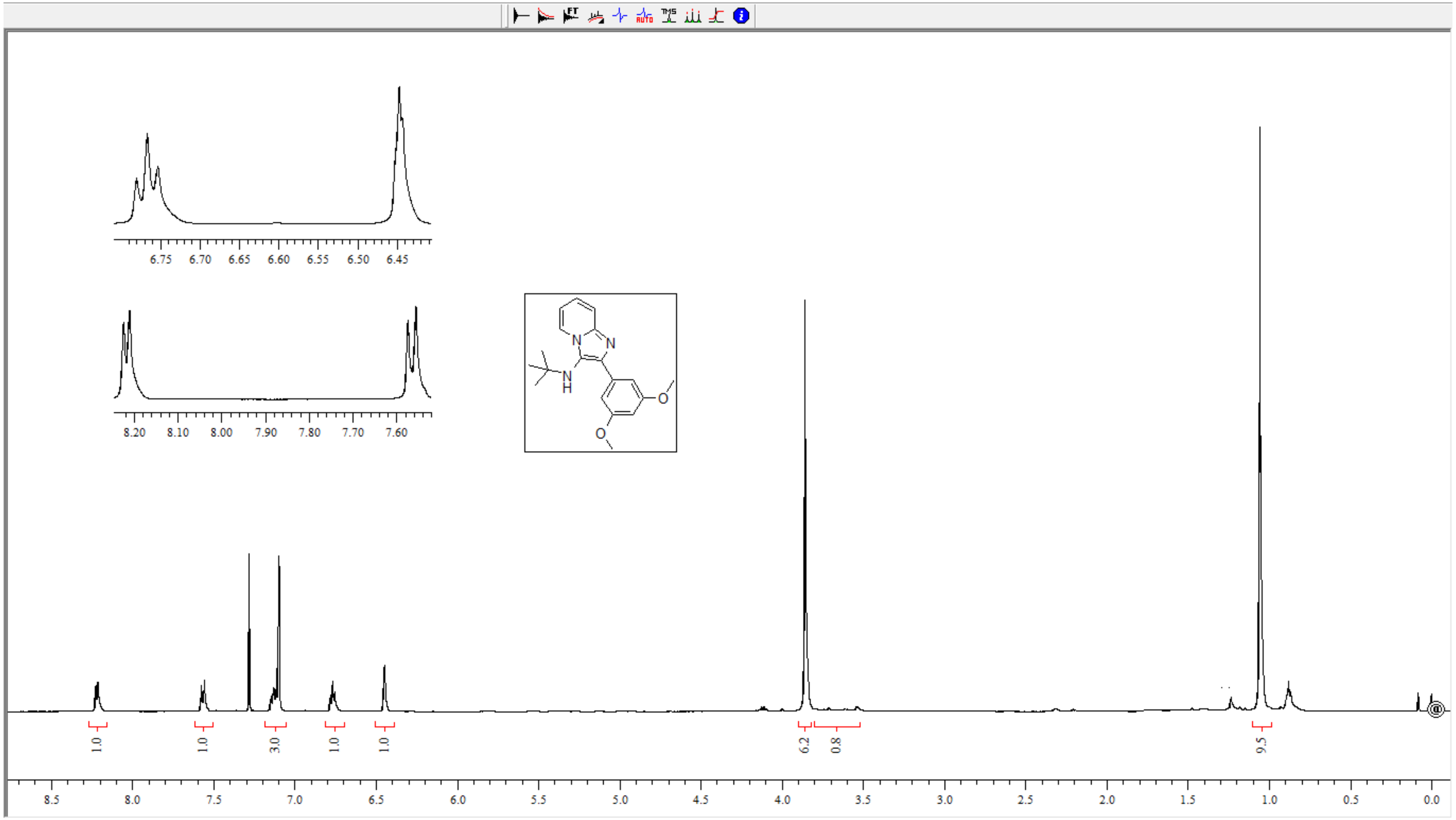

^{1}H NMR of compound **7b**

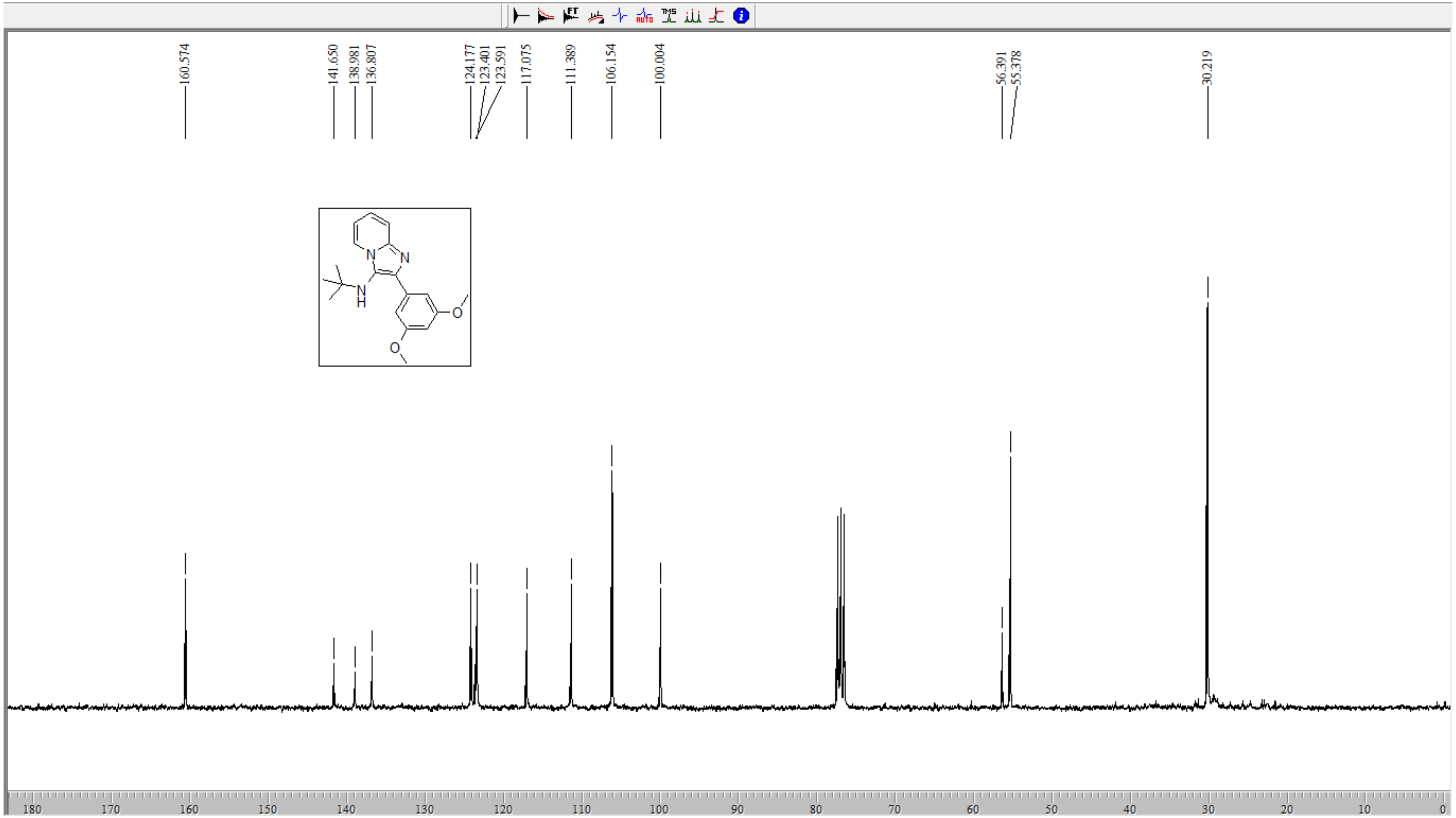

^{13}C NMR of compound **7b**

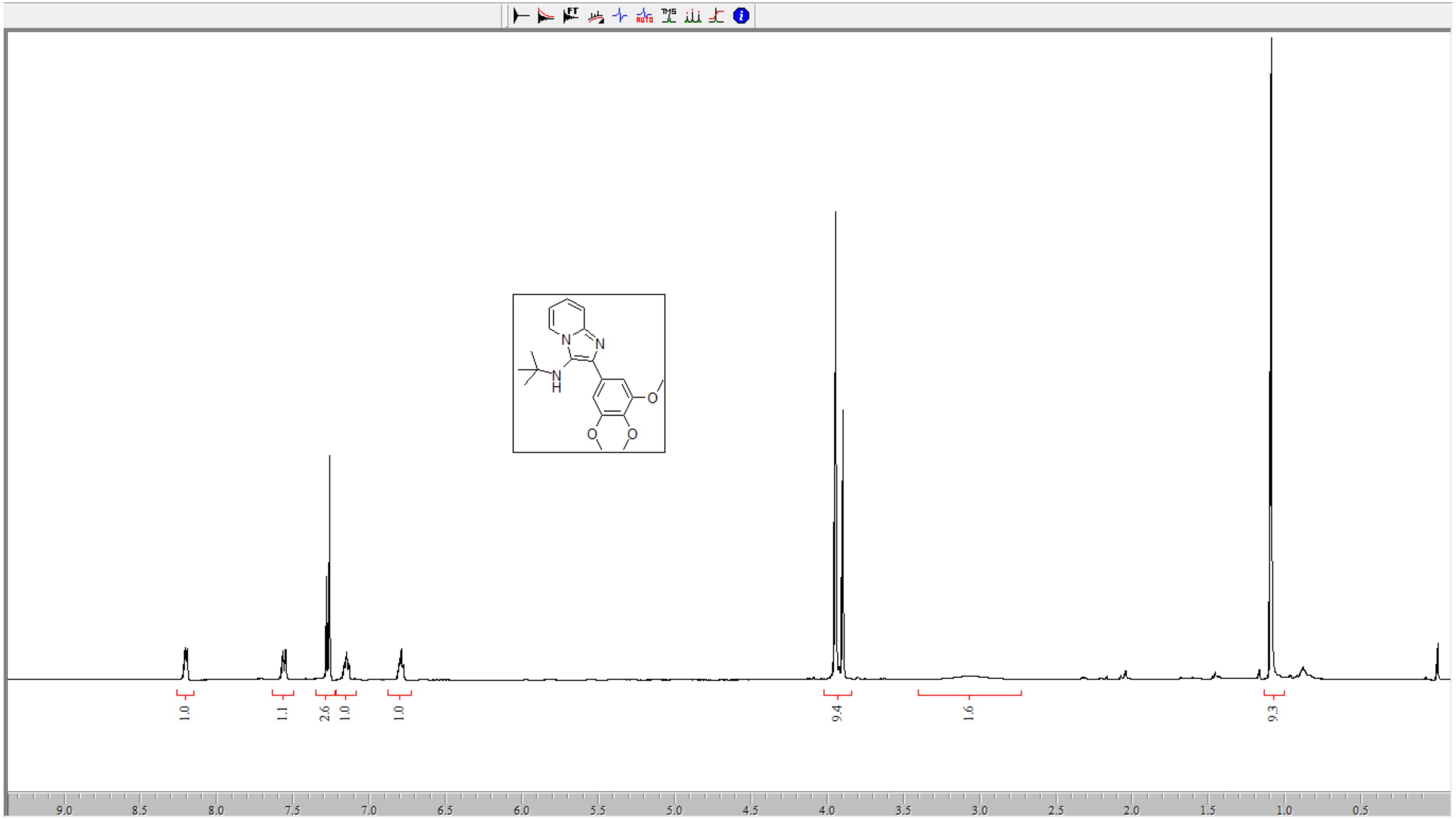

^{1}H NMR of compound **7c**

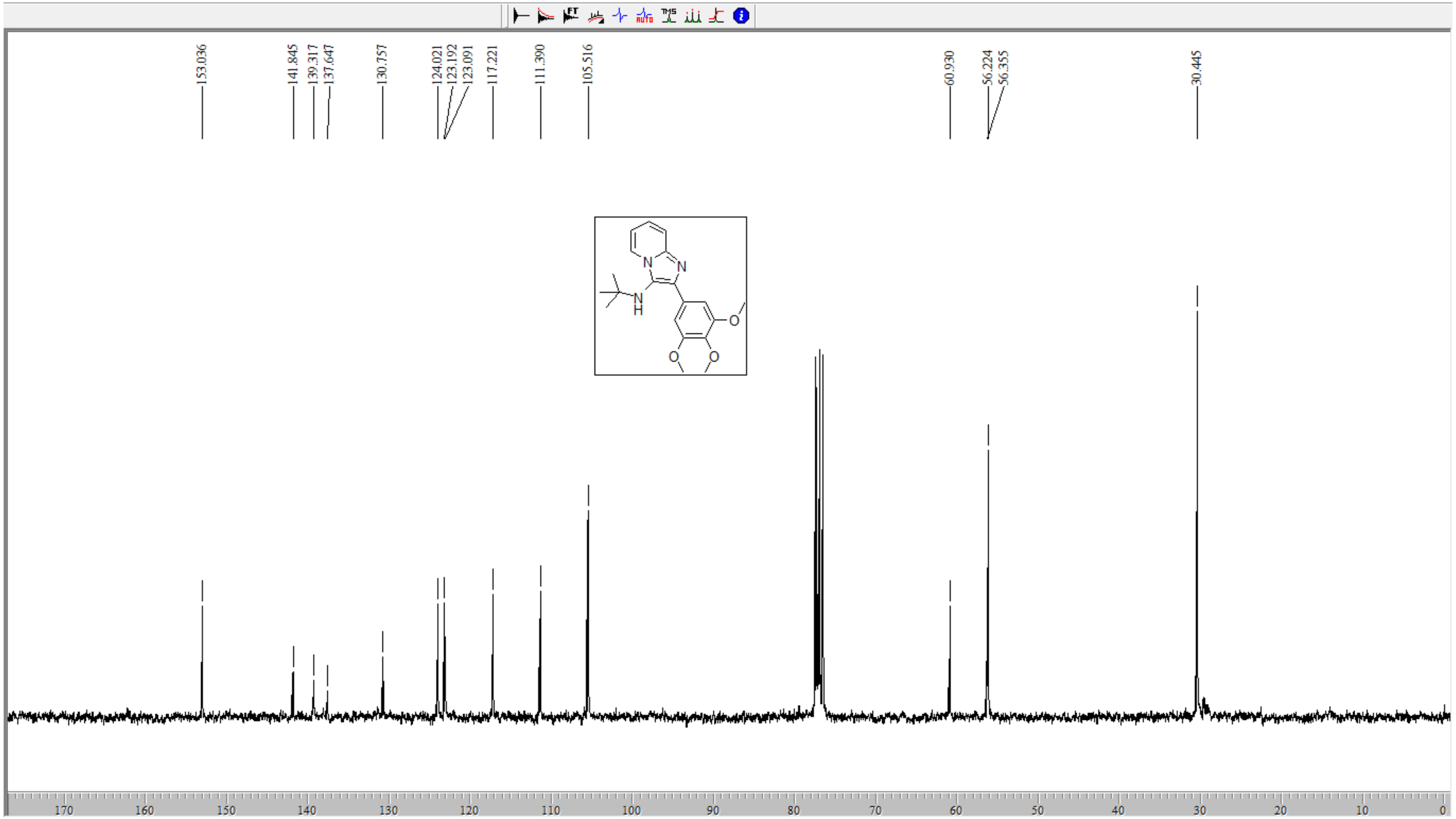

^{13}C NMR of compound **7c**

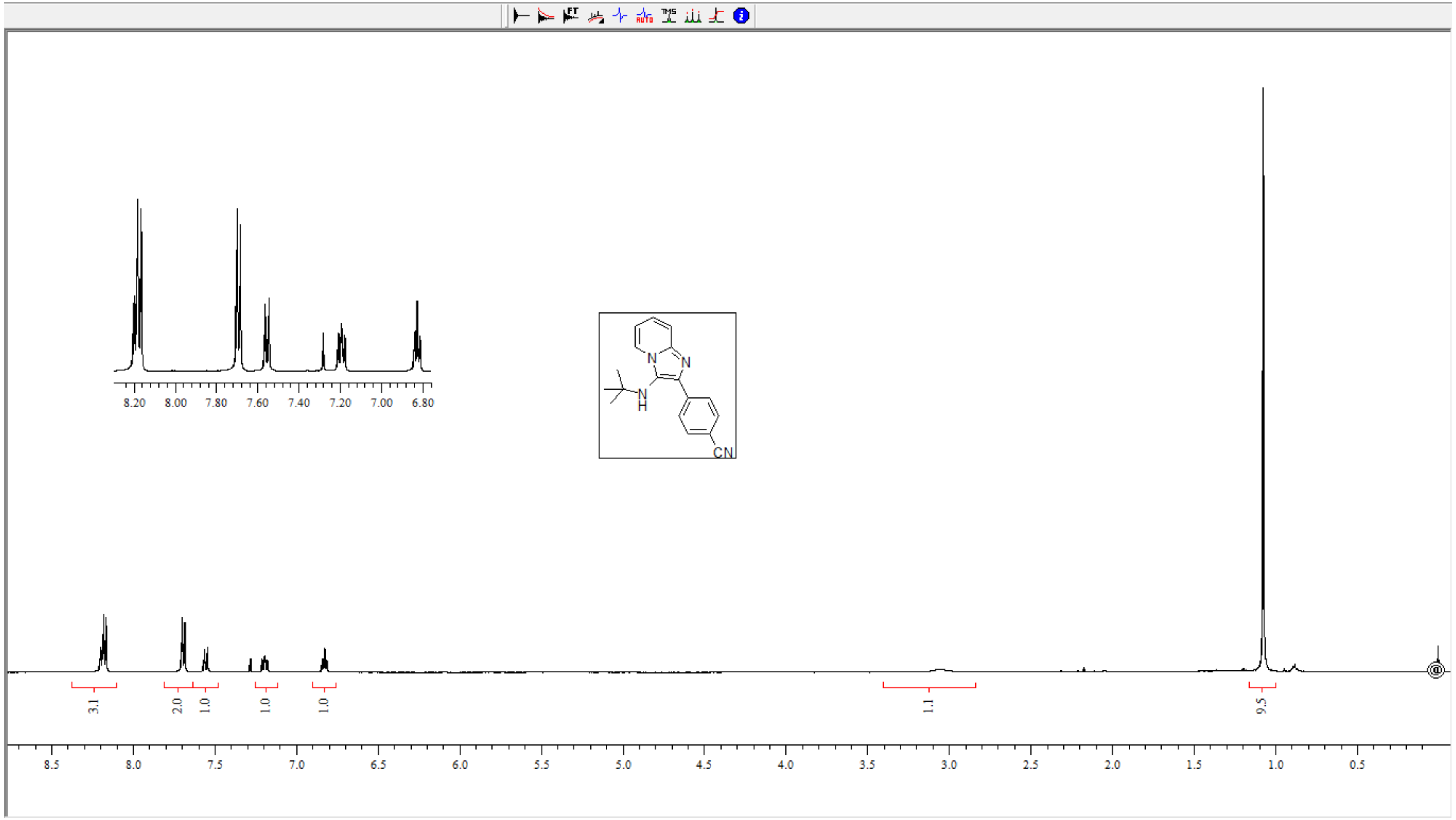

^{1}H NMR of compound **7d**

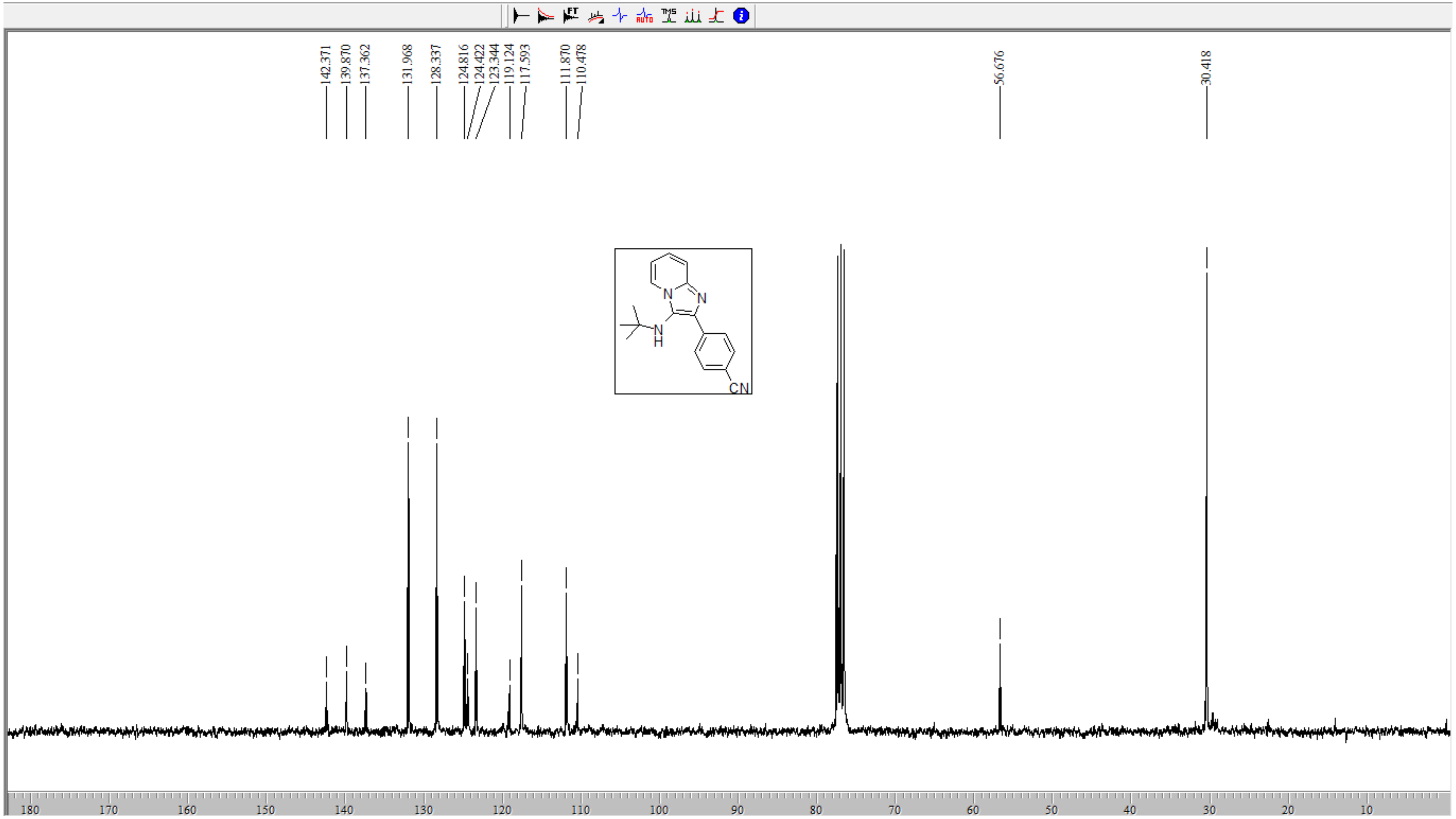

^{13}C NMR of compound **7d**
